# Identification of 34 genes conferring genetic and pharmacological risk for the comorbidity of schizophrenia and smoking behaviors

**DOI:** 10.18632/aging.102735

**Published:** 2020-02-03

**Authors:** Yunlong Ma, Jingjing Li, Yi Xu, Yan Wang, Yinghao Yao, Qiang Liu, Maiqiu Wang, Xinyi Zhao, Rongli Fan, Jiali Chen, Bin Zhang, Zhen Cai, Haijun Han, Zhongli Yang, Wenji Yuan, Yigang Zhong, Xiangning Chen, Jennie Z. Ma, Thomas J. Payne, Yizhou Xu, Yuping Ning, Wenyan Cui, Ming D. Li

**Affiliations:** 1 State Key Laboratory for Diagnosis and Treatment of Infectious Diseases, National Clinical Research Center for Infectious Diseases, Collaborative Innovation Center for Diagnosis and Treatment of Infectious Diseases, The First Affiliated Hospital, Zhejiang University School of Medicine, Hangzhou, China; 2 Department of Cardiology, Affiliated Hangzhou First People’s Hospital, Zhejiang University School of Medicine, Hangzhou, China; 3 Institute of Personalized Medicine, University of Nevada at Las Vegas, Las Vegas, NV 89154, USA; 4, Department of Public Health Sciences, University of Virginia, Charlottesville, VA 22904, USA; 5 Department of Otolaryngology and Communicative Sciences, University of Mississippi Medical Center, Jackson, MS 39216, USA; 6 The Affiliated Brain Hospital of Guangzhou Medical University, Guangzhou, China; 7 Research Center for Air Pollution and Health, Zhejiang University, Hangzhou, China

**Keywords:** schizophrenia, tobacco smoking, multi-omics, susceptibility genes, pathways, GWAS

## Abstract

The prevalence of smoking is significantly higher in persons with schizophrenia (SCZ) than in the general population. However, the biological mechanisms of the comorbidity of smoking and SCZ are largely unknown. This study aimed to reveal shared biological pathways for the two diseases by analyzing data from two genome-wide association studies with a total sample size of 153,898. With pathway-based analysis, we first discovered 18 significantly enriched pathways shared by SCZ and smoking, which were classified into five groups: postsynaptic density, cadherin binding, dendritic spine, long-term depression, and axon guidance. Then, by using an integrative analysis of genetic, epigenetic, and expression data, we found not only 34 critical genes (e.g., *PRKCZ*, *ARHGEF3*, and *CDKN1A*) but also various risk-associated SNPs in these genes, which convey susceptibility to the comorbidity of the two disorders. Finally, using both *in vivo* and *in vitro* data, we demonstrated that the expression profiles of the 34 genes were significantly altered by multiple psychotropic drugs. Together, this multi-omics study not only reveals target genes for new drugs to treat SCZ but also reveals new insights into the shared genetic vulnerabilities of SCZ and smoking behaviors.

## INTRODUCTION

Cigarette smoking is a common brain disorder that is extremely harmful to the individual and society. Smoking prevalence is much higher among individuals with mental disorders than in the general population [1]. In developed countries, the smoking rate among the general population has decreased over recent decades, whereas there has been no decrease among mental health patients. In particular, smoking shows a significant association with schizophrenia (SCZ) [2], with around 80% of schizophrenic patients being smokers [3, 4]. There are three primary hypotheses intended to elucidate the comorbidity of smoking and SCZ [5–8]. One dominant hypothesis contends that smoking is able to remedy, at least partly, the symptoms of SCZ [6]. There are two main lines of evidence for this hypothesis. One is that nicotine intake enhances the metabolizing of anti-psychotic drugs [7]; the other is that nicotine promotes the release of several neurotransmitters (e.g., dopamine, glutamate, and serotonin) and improves patient performance in memory and attention [4, 5]. The second hypothesis is that given that both diseases are highly influenced by genetics [9, 10], there might exist shared genetic components predisposing to both SCZ and smoking behaviors. Recently, many genetics-based studies have lent support to this hypothesis [11–14]. The third hypothesis is that smoking leads to the onset of SCZ in view of the fact that smoking initiation typically predates the appearance of SCZ [8]. A meta-analysis of cross-sectional and prospective studies reported that daily cigarette smoking was associated with an earlier age of onset of a psychosis [15]. Consistently, a large prospective study of Swedish registry data demonstrated that both light and heavy smoking were highly associated with a greater risk for SCZ [16]. To some extent, these hypotheses are mutually non-exclusive and may collectively contribute to the correlation of SCZ with smoking.

Increasing neuroimaging evidence supports the presence of an association between SCZ and smoking [17]. An fMRI study [18] showed that nicotine can restore deficient sensorimotor gating, which is associated with activation of the limbic regions and striatum in both SCZ patients and healthy controls. Compared with nonsmoking SCZ patients and healthy subjects, SCZ patients with concurrent nicotine addiction have reduced grey matter volumes [19]. Furthermore, it is well documented that nicotine increases the release of dopamine, acetylcholine, glutamate, norepinephrine, and serotonin, which are all implicated in the etiology of SCZ [4].

Accumulating evidence has revealed shared genetic components for SCZ and smoking [11–14]. One of the most promising findings is that variants in the *CHRNA5/A3/B4* cluster on chromosome 15q24 are associated not only with nicotine dependence (ND) [12] but also with SCZ [11]. By using experimental evidence from animal models of addiction and SCZ, Koukouli et al. [20] found that in rodents, nicotine addiction-associated polymorphisms in *CHRNA5* provoke a decrease in neuronal activity, which mirrors the hypofrontiality detected in SCZ or addictive patients. Furthermore, there were multiple susceptibility genes reported to be associated with both SCZ and smoking risk, including *DRD2* [11, 21–23], *BDNF* [24], and *COMT* [25, 26], to name a few. As we know, psychiatric disorders including SCZ are highly comorbid diseases [27–30]; thus there exists a great comorbidity on the relations between SCZ and substance addictions [31, 32]. For example, a good number of reports [33–37] have concentrated on the genetic effect of the Val158Met polymorphism in *COMT* on the comorbidity of SCZ and substance addictions. In addition, Chen et al. [13] reported that ND is positively correlated with a polygenic risk score for SCZ whereas SCZ is positively correlated with the polygenic risk score for cotinine concentration. A recent study [38] revealed a statistically significant genetic correlation between SCZ and several smoking-related phenotypes.

To make further progress in the prevention and treatment of SCZ and smoking, it is essential to identify the etiologic biological pathways and susceptibility genes underlying the comorbidity of both disorders. Earlier genetics association studies or pathway-based studies focused primarily on either SCZ or smoking [39–42]. To date, only two reports [13, 14] investigated the genetic relations between SCZ and smoking based on pathway analysis results for a limited number of candidates or significant genes. To the best our knowledge, there has been no study providing an integrative genomics analysis based on multi-omics data and biological pathways for both SCZ and smoking. Therefore, the primary objective of this study was to identify susceptibility SNPs, genes, and pathways for the comorbidity of SCZ and smoking with the use of multi-omics data from various sources (Supplementary Figure 1).

## RESULTS

### GWAS-based enrichment analysis for SCZ and smoking behaviors

**Figure 1:**
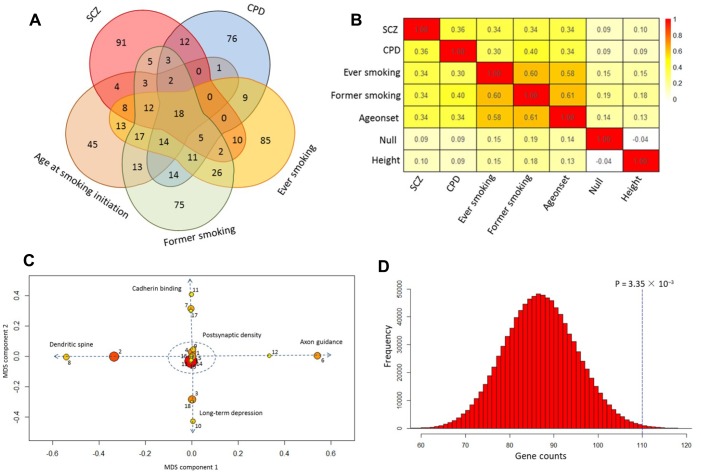
**Shared pathways of SCZ and smoking phenotypes.** (**A**) Venn diagram of significantly enriched pathways (*q* value < 0.1) for SCZ, CPD, ever smoking, former smoking, and age at smoking initiation. (**B**) Heatmap of the correlation among SCZ, CPD, ever smoking, former smoking, age at smoking initiation, null, and height based on the Z score of pathway enrichment. (**C**) Multidimensional scaling plot of 18 shared pathways for SCZ and smoking behaviors. Circular ring sizes reflect number of genes in the pathway (range 18–284). Color indicates the significance of the pathway (red marks the significant pathways with lowest P values). Arabic numerals are the pathway numbers as shown in Table 1. (**D**) Computer permutation analysis of 590 genes associated with SCZ in 84 shared pathways.

Our pathway analysis of GWAS summary statistics on SCZ and smoking phenotypes revealed 175, 172, 233, 225, and 158 significantly enriched pathways (*q* value < 0.1) for SCZ, CPD, ever smoking, former smoking, and age at smoking initiation, respectively. There were 84 pathways with a *q* value < 0.1 for SCZ that were in common with that for at least one smoking phenotype (Figure 1A and Supplementary Table 2). Of them, 18 showed significant enrichment in SCZ and all four smoking phenotypes (Table 1 and Figure 1A). For SCZ, the most significant pathway was postsynaptic density (P < 3.04 × 10^−14^), which is consistent with a previous report [43]. This postsynaptic density pathway also showed highly significant enrichment in all smoking-related behaviors, with P values from 3.09 × 10^−6^ for age at smoking initiation to 1.43 × 10^−13^ for CPD (Table 1), confirming an important role for postsynaptic density in these two disorders. In contrast, we found that the enriched pathways in smoking behaviors had a much higher overlap with SCZ than that in the traits of null and height (Figure 1B, Supplementary Figures 2 and 3). Next, by using multidimensional scaling (MDS) for shared genes, we found that the 18 common pathways were categorized into five clusters (Figure 1C and Table 1): postsynaptic density (Cluster #1), cadherin binding (Cluster #2), dendritic spine (Cluster #3), long-term depression (Cluster #4), and axon guidance (Cluster #5).

**Table 1:** Top 18 pathways shared between schizophrenia and smoking behaviors.

**Pathway Number**	**Pathway ID**	**Description**	**Category**	**SCZ P-value**	**CPD P-value**	**Ever smoking P-value**	**Former smoking P-value**	**Age at smoking initiation P-value**
1	GO:0014069	Postsynaptic density	Cluster #1	3.04 × 10^−14^	1.43× 10^−13^	1.21 × 10^−9^	1.50 × 10^−6^	3.09 × 10^−6^
2	GO:0045211	Postsynaptic membrane	Cluster #3	7.44 × 10^−13^	4.97 × 10^−11^	1.10 × 10^−10^	5.92 × 10^−9^	5.52 × 10^−6^
3	GO:0045202	Synapse	Cluster #4	3.01 × 10^−9^	2.76 × 10^−13^	6.88 × 10^−15^	1.91 × 10^−9^	4.88 × 10^−8^
4	GO:0030425	Dendrite	Cluster #1	9.46 × 10^−9^	1.34 × 10^−7^	0.0043	0.0011	0.0001
5	GO:0007268	Synaptic transmission	Cluster #1	3.22 × 10^−8^	8.03 × 10^−10^	1.85 × 10^−11^	3.22 × 10^−11^	9.84 × 10^−6^
6	GO:0007411	Axon guidance	Cluster #5	5.89 × 10^−8^	8.28 × 10^−6^	6.04 × 10^−12^	8.10 × 10^−12^	3.64 × 10^−9^
7	GO:0005001	Transmembrane receptor protein tyrosine phosphatase activity	Cluster #2	7.21 × 10^−7^	0.0010	5.50 × 10^−8^	0.0086	7.84 × 10^−5^
8	GO:0043197	Dendritic spine	Cluster #3	4.34 × 10^−6^	0.0009	0.0007	0.0011	0.0048
9	hsa05412	Arrhythmogenic right ventricular cardiomyopathy (ARVC)	Cluster #1	1.06 × 10^−5^	0.0025	4.17 × 10^−6^	1.36 × 10^−5^	0.0008
10	hsa04912	GnRH signaling pathway	Cluster #4	7.76 × 10^−5^	0.0056	8.06 × 10^−5^	0.0042	0.0001
11	GO:0045296	Cadherin binding	Cluster #2	0.00014	0.0028	0.0008	0.0004	5.83 × 10^−5^
12	GO:0007156	Homophilic cell adhesion	Cluster #5	0.00049	0.0011	1.48 × 10^−5^	2.00 × 10^−7^	1.50 × 10^−7^
13	GO:0005216	Ion channel activity	Cluster #1	0.00051	2.95 × 10^−13^	0.0001	1.29 × 10^−5^	0.0001
14	GO:0051056	Regulation of small GTPase mediated signal transduction	Cluster #1	0.00053	0.0002	1.14 × 10^−6^	4.88 × 10^−5^	0.0002
15	hsa04020	Calcium signaling pathway	Cluster #1	0.0006	0.0056	2.74 × 10^−10^	2.38 × 10^−6^	2.99 × 10^−6^
16	GO:0006813	Potassium ion transport	Cluster #1	0.00074	0.0038	2.73 × 10^−6^	0.0017	9.14 × 10^−5^
17	GO:0007626	Locomotory behavior	Cluster #2	0.0012	9.36 × 10^−14^	0.0032	3.10 × 10^−5^	0.0030
18	hsa04730	Long-term depression	Cluster #4	0.0042	7.10 × 10^−5^	0.0012	2.85 × 10^−6^	0.0009

**Figure 2:**
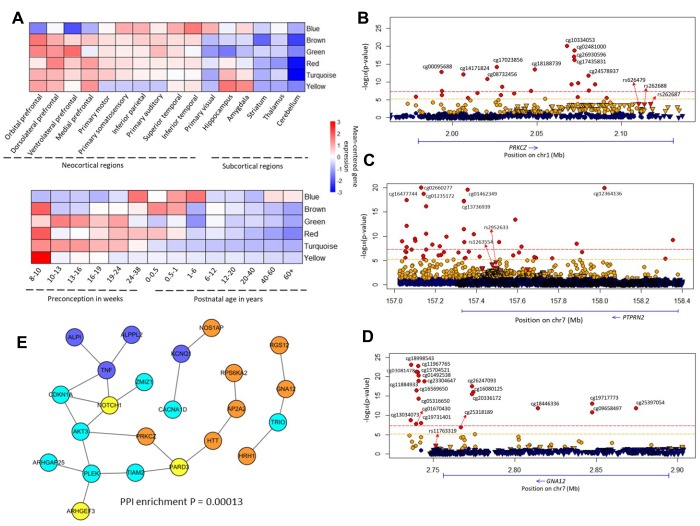
**Brain co-expression modules and common methylated genes.** (**A**) Regional and temporal patterns of gene expression mean-centered by the extent of gene expression within each module. (**B**–**D**) Regional plot of association between genetic and epigenetic variants of the *PRKCZ*, *PTPRN2*, *GNA12* loci, and SCZ, respectively. Circular symbols indicate the association of CpG loci with SCZ (red represents loci significantly associated with SCZ with P ≤ 6.07 × 10^-6^; orange indicates loci with 6.07 × 10^-6^ < P ≤ 0.05; blue marks loci with P > 0.05). Triangular symbols indicate association of SNPs with SCZ (red represents top-ranked SNPs associated with SCZ; orange indicates SNPs associated with SCZ with P ≤ 0.05; blue marks SNPs with P > 0.05). (**E**) Gene subnetwork constituted from the 34 common genes. The protein–protein interactions are according to the database of STRING (v. 10.5). We used Cytoscape software to visualize the subnetwork. The color of a node indicates the co-expression module of the genes.

**Figure 3:**
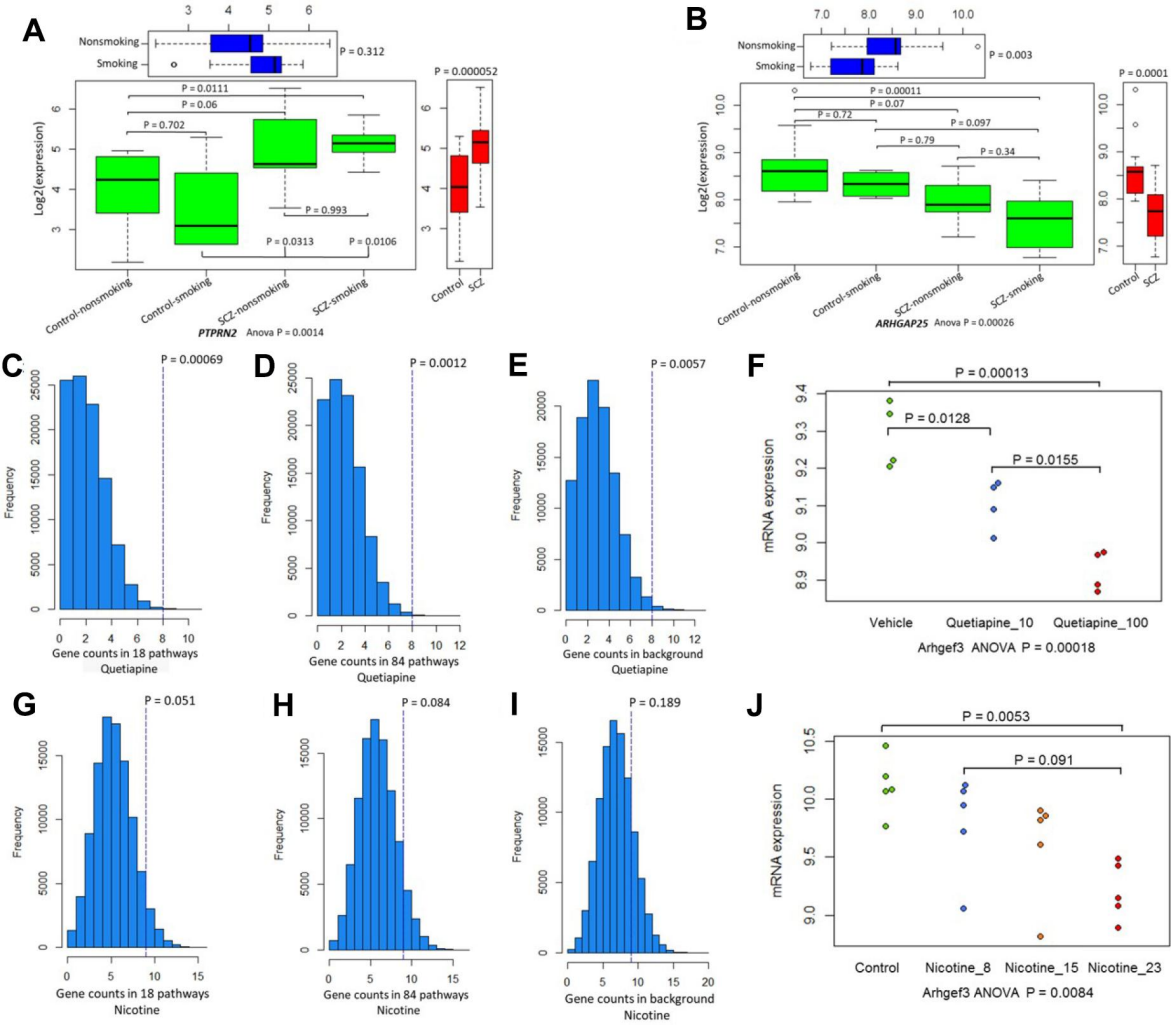
**The differential expression patterns of 34 candidate genes.** (**A**) Pattern of *PTPRN2* in SCZ patients and controls divided by smoking status. (**B**) Pattern of *ARHGAP25* in SCZ patients and controls divided by smoking status. (**C**–**E**) Permutation analysis of 34 candidate genes in 18 common pathways (N = 1,588 genes), 84 common pathways (N =3,334 genes), and background genes (N = 45,037) for quetiapine treatment. (**F**) Plot summarizing *Arhgef3* expression changes in quetiapine (at doses of 10 or 100 mg/kg)-treated mice. (**G**–**I**) Permutation analysis of 34 candidate genes in 18 common pathways (N = 1,588), 84 common pathways (N = 3,334), and background genes (N = 31,047) for nicotine treatment. (**J**) Plot summarizing *Arhgef3* expression alterations in nicotine (at doses of 8 μg, 15 μg, and 23 μg/L)-treated mice.

Following the pathway analysis of GWAS summary data on SCZ and smoking, we performed gene-based analysis on the same dataset. For SCZ, we found 590 significantly associated genes after correction for multiple testing. Of them, 236 were located in 108 previously identified loci [11]. Even though numerous genes were significantly associated with CPD and former smoking, no one gene reached significance for ever-smoking or age at smoking initiation. We found 208 genes significantly associated with SCZ that also were associated with at least one of the four smoking phenotypes (Supplementary Table 3). Of these 208 shared genes, 70 were located in 108 previously reported loci for SCZ (Supplementary Figure 4). In addition, by performing computer permutation analysis, we found that the genes identified in our gene-based analysis were significantly overrepresented in the identified common pathways (P < 0.003; Figure 1D and Supplementary Figures 5 and 6).

### Brain gene expression of common pathways

By analyzing brain expression data, we found 1,443 genes from the 84 common pathways to be coexpressed in six modules (Supplementary Figures 7–9), and the top 10 “hub” genes in each module were demonstrated to have highly intramodular and intermodular connections (Supplementary Figures 10 and 11). Interestingly, we observed two distinct and dynamic expression patterns in different brain regions and at different developmental stages (Figure 2A). For example, one module (marked in yellow) has a greater than twofold difference between brain regions, and two modules (brown and red) showed twofold temporal changes (Supplementary Table 4).

### Common methylated genes in both smoking and schizophrenia

By comparison with the previously reported findings [44], we found 149 module genes that overlapped with smoking-associated DNA-methylated genes; and 38 of these genes had at least two independent pieces of supporting evidence (Supplementary Table 5). Next, we performed a differential methylation analysis of 8,236 CpG loci in 149 genes related to SCZ and found that 822 CpG loci, mapped to 124 genes (124/149; binomial test: P < 2.2 × 10^−16^), showed significant association with SCZ (P < 6.07 × 10^−6^; Supplementary Table 5). For the 38 genes, 413 significantly methylated CpG loci were annotated in 34 of the genes, and there existed a high consistency of shared methylated genes between SCZ and smoking (34/38; binomial test: P = 6.04 × 10^−7^). Of note, 16 of these 34 candidate genes have been reported extensively to be associated with SCZ (Table 2 and Supplementary Table 6).

**Table 2:** Multiple lines of evidence support involvement of 34 genes in the comorbidity of both SCZ and smoking.

**Gene names**	**Chromosome**	**Brain coexpression modules**	**Smoking-associated methylation genes**	**SCZ-associated methylation genes**	**cis-meQTLs in brain samples**	**Genes enriched in the PPI subnetwork**	**Differential expressed genes in SCZ patients stratified by smoking: Anova P values**	**Differential expression genes treated by quetiapine or nicotine: Anova P values**	**Genes risk to SCZ in previous studies**
*TRIO*	Chr5	Turquoise	Yes	Yes	Yes	Yes	Not significant	0.031 (Quetiapine)	Yes (PMID: 21422296)
*GNA12*	Chr7	Brown	Yes	Yes	Yes	Yes	Not significant	0.049 (Quetiapine)	Yes (PMID: 22792057)
*KCNQ1*	Chr11	Blue	Yes	Yes	Yes	Yes	Not significant	0.0073 (Nicotine); 0.3 (Quetiapine)	Yes (PMID: 26971948; 28188958)
*AKT3*	Chr1	Turquoise	Yes	Yes	Yes	Yes	0.0395	0.018 (Nicotine)	Yes (PMID: 25056061; 28467426; 29173281; 23974872; 25599223)
*ARHGEF3*	Chr3	Yellow	Yes	Yes	Yes	Yes	0.00723	0.00018 (Quetiapine); 0.0084 (Nicotine)	No data
*CACNA1D*	Chr3	Turquoise	Yes	Yes	Yes	Yes	0.052	Not significant	Yes (PMID: 29214423; 26255836; 24996399)
*RGS12*	Chr4	Brown	Yes	Yes	Yes	Yes	0.0058	0.053 (Nicotine)	Yes (PMID: 25420024)
*RPS6KA2*	Chr6	Brown	Yes	Yes	Yes	Yes	Not significant	0.05 (Nicotine)	No data
*NOTCH1*	Chr9	Yellow	Yes	Yes	Yes	Yes	Not significant	0.0166 (Quetiapine)	Yes (PMID: 26232790)
*PARD3*	Chr10	Yellow	Yes	Yes	Yes	Yes	0.054	0.018 (Nicotine)	Yes (PMID: 22969987)
*AP2A2*	Chr11	Brown	Yes	Yes	No	Yes	Not significant	0.0032 (Nicotine)	Yes (PMID: 23811784)
*NOS1AP*	Chr1	Brown	Yes	Yes	Yes	Yes	Not significant	Not significant	Yes (PMID:26861996; 25542305; 20605702; 16146415; 15065015; 12116186; 19077434)
*HRH1*	Chr3	Brown	Yes	Yes	No	Yes	Not significant	Not significant	Yes (PMID:28400155; 27855565)
*HTT*	Chr4	Brown	Yes	Yes	No	Yes	Not significant	0.0085 (Quetiapine); 0.075 (Nicotine)	No data
*CDKN1A*	Chr6	Turquoise	Yes	Yes	No	Yes	Not significant	0.00085 (Quetiapine); 0.23 (Nicotine)	Yes (PMID: 23549417)
*TIAM2*	Chr6	Turquoise	Yes	Yes	Yes	Yes	0.062	Not significant	No data
*TNF*	Chr6	Blue	Yes	Yes	No	Yes	Not significant	0.06 (Nicotine)	Yes (PMID: 29499967; 29706448)
*CNTNAP2*	Chr7	Brown	Yes	Yes	No	No	0.087	0.0085 (Quetiapine)	Yes (PMID: 29610457; 25852443; 23123147)
*MAD1L1*	Chr7	Brown	Yes	Yes	Yes	No	Not significant	0.024 (Nicotine)	Yes (PMID: 25056061; 26193471; 26528791)
*PTPRN2*	Chr7	Red	Yes	Yes	Yes	No	0.00142	0.0028 (Nicotine)	No data
*CLCN6*	Chr1	Green	Yes	Yes	Yes	No	Not significant	Not significant	No data
*PRKCZ*	Chr1	Brown	Yes	Yes	Yes	Yes	Not significant	Not significant	No data
*ALPI*	Chr2	Blue	Yes	Yes	Yes	Yes	Not significant	Not significant	No data
*ALPPL2*	Chr2	Blue	Yes	Yes	Yes	Yes	Not significant	Not significant	No data
*ARHGAP25*	Chr2	Turquoise	Yes	Yes	No	Yes	0.00026	Not significant	No data
*PLEK*	Chr2	Turquoise	Yes	Yes	No	Yes	0.043	0.018 (Nicotine)	No data
*ZMIZ1*	Chr10	Turquoise	Yes	Yes	Yes	Yes	0.041	0.0128 (Quetiapine)	No data
*TBC1D14*	Chr4	Turquoise	Yes	Yes	Yes	No	0.03	Not significant	No data
*SORBS1*	Chr10	Turquoise	Yes	Yes	Yes	No	Not significant	Not significant	No data
*TGFBR3*	Chr1	Turquoise	Yes	Yes	No	No	Not significant	Not significant	No data
*CPOX*	Chr3	Brown	Yes	Yes	No	No	Not significant	Not significant	No data
*TIGIT*	Chr3	Blue	Yes	Yes	No	No	Not significant	Not significant	No data
*GPSM3*	Chr6	Blue	Yes	Yes	No	No	0.0014	Not significant	No data
*DUSP4*	Chr8	Turquoise	Yes	Yes	No	No	Not significant	Not significant	No data

Through using these 34 genes to construct a subnetwork, we found 23 of 34 genes significantly enriched in that subnetwork (PPI enrichment: P = 1.3 × 10^−4^; Figure 2E). Many of these genes had several significantly SCZ-associated CpG loci (Figure 2B–2D and Supplementary Figures 12 and 13). For example, SCZ-associated cg10334053 (P = 8.66 × 10^−21^), cg02481000 (P = 1.7 × 10^−19^), and cg18188739 (P = 3.56 × 10^−14^) were all located in *PRKCZ* (Figure 2B). Of note, cg18188739 appeared to be associated with smoking at a genome-wide significance level (P = 2.86 × 10^−8^) [45].

### Differential expression profiles of 34 candidate genes

We next examined the difference in the influence of smoking on gene expression between SCZ patients and healthy controls and found that 9 of 34 genes showed significantly different expression in SCZ subjects and controls stratified by smoking status (Table 2; Supplementary Table 7; Figure 3; Supplementary Figures 14–16). Furthermore, several genes showed distinct expression profiles in schizophrenic hiPSC-derived neurons (Supplementary Figure 17) compared with the control neurons. Consistent with previous studies [11, 39], most of these candidate genes were highly expressed in human brain regions (Supplementary Figures 18–26).

### The pharmacological effects of 34 candidate genes

Based on the DGIdb database, we found that 13 of the 34 genes (38.24%) were targeted by at least one drug. For example, *HRH1, CDKN1A,* and *TNF* are targeted by various psychotropic drugs. Also, 25 (73.53%) belong to one or more potentially “druggable” gene categories (Supplementary Figure 27). Consistently, the expression of several genes in the mouse brain was significantly modulated by quetiapine (Supplementary Figure 28) and nicotine (Supplementary Figure 29) in a dose-dependent pattern. Further, the count of significantly quetiapine- and nicotine-induced genes among these 34 genes were prominently higher than that of genes in 18 (Figure 3C, 3G) and 84 common pathways (Figure 3D, 3H) or all background genes (Figure 3E, 3I). For example, the expression of *Arhgef3* was downregulated by both quetiapine (Figure 3F: ANOVA P = 0.00018) and nicotine (Figure 3J: ANOVA P = 0.0084). In addition, we found that many genes showed different expression profiles in the mouse striatum at four time points of treatment with 18 major psychotropic drugs (Supplementary Figures 30–32).

### Cis-regulatory effects of SNPs in 34 candidate genes

**Figure 4:**
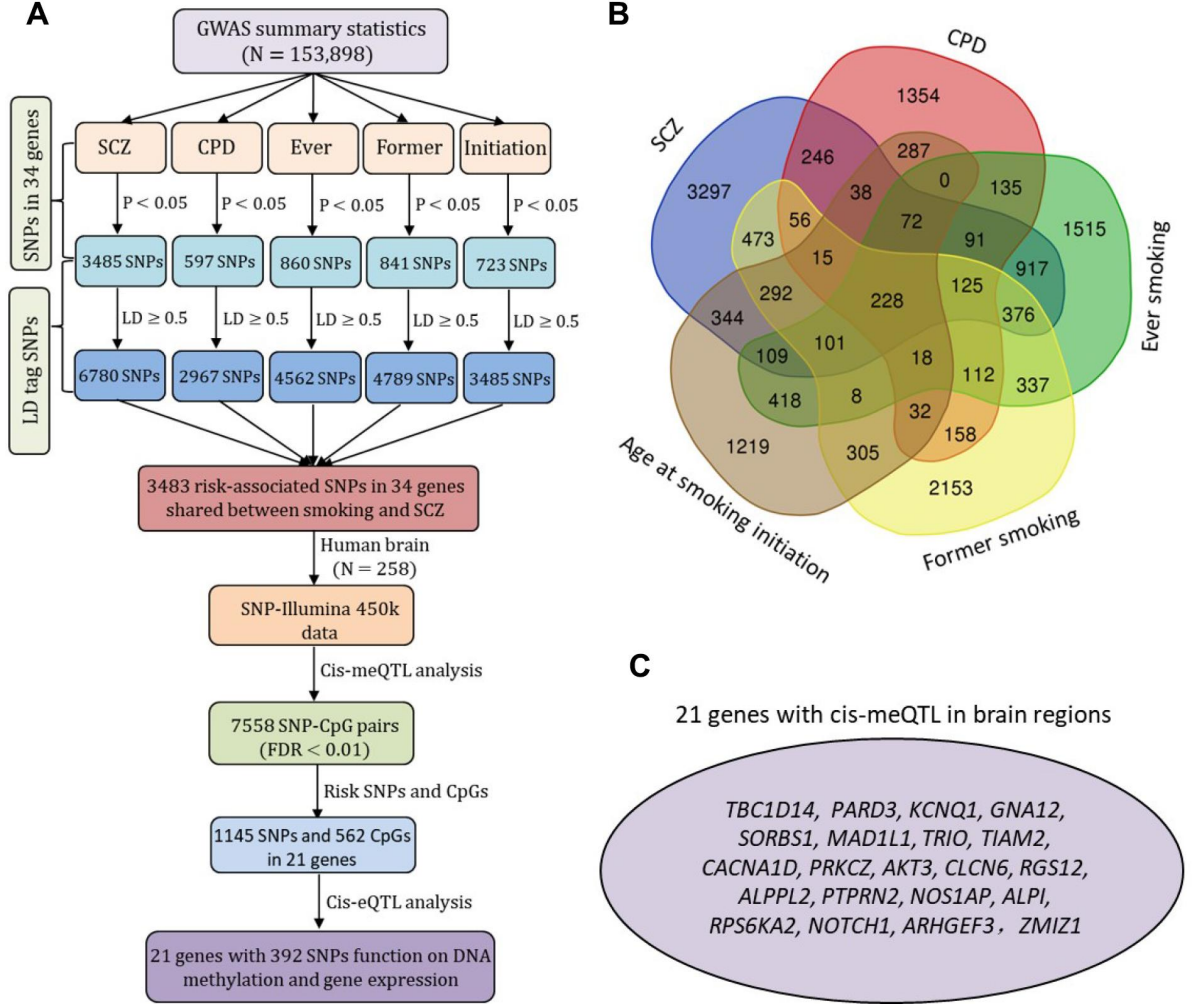
**The *cis*-acting regulatory effects of risk-associated SNPs in 34 common genes on DNA methylation and gene expression.** (**A**) Schematic of risk-associated SNPs (P < 0.05) compiled from two large-scale meta-GWASs on SCZ (N = 79,845) and smoking behaviors (N = 74,053) and 3,483 risk-associated SNPs in 34 genes shared by SCZ and smoking for *cis*-meQTL and *cis*-eQTL analysis. The SNPs in strong LD with risk-associated SNPs (LD cutoff r^2^ ≥ 0.5) were generated according to the 1000 Genome European Phase 3 panel as reference. (**B**) Venn diagram of risk-associated SNPs (P < 0.05) for SCZ, CPD, ever smoking, former smoking, and age at smoking initiation. (**C**) Plot shows the 21 promising genes with SNPs had *cis*-regulatory roles in both DNA methylation and gene expression.

Based on two large GWAS datasets, there were 3,483 risk-suggested SNPs shared by SCZ and at least one smoking phenotype (Figure 4A and Supplementary Figure 33), and 228 of these were common to SCZ and all four smoking phenotypes (Figure 4B). A great number of SNPs among these identified 3,483 risk SNPs were located within different types of regulatory elements in brain tissues and neuroblastoma cell lines (Supplementary Figures 34 and 35). We performed a *cis*-meQTL analysis in human brain samples, which showed 7,558 significant SNP–CpG methylation pairs with 1,145 SNPs in 21 genes (FDR < 0.01; Figure 4A and Supplementary Figure 36). A number of the 392 variants in the 21 genes had *cis*-regulatory roles in both DNA methylation and gene expression (Figure 4A, 4C).

## DISCUSSION

Cigarette smoking is highly concurrent with SCZ [2, 18, 46]. Individuals with mental disorders such as SCZ are at higher risk for developing smoking-related diseases, which include cardiovascular and respiratory diseases and various cancers [5]. Based on a national cohort study including about 6 million Swedish adults, Crump et al. [47] found a significant portion of the morbidity and premature death in persons with SCZ was ascribable to ischemic heart disease and cancers. Considering that the co-occurrence of SCZ and smoking has greatly impacted public health, it is important to understand the pathogenesis of the comorbidity of the two diseases. Previous studies concentrated largely on the investigation of the genetic mechanisms of either SCZ [11] or smoking [12]. Similarly, studies employing pathway-based enrichment analysis so far have focused mainly on either SCZ or smoking [39–42].

Recently, multiple large-scale GWAS studies [27–30, 38] consistently revealed that there exists a considerable genetic correlation between SCZ and smoking-related traits. However, susceptibility variants, genes, and biological pathways for the comorbidity remain largely unknown. In the present study, by conducting an integrative genomics analysis of large-scale GWAS data with multi-omics data, we intended to identify the risk SNPs, genes, and pathways implicated in the etiology of these two comorbid diseases.

We first conducted pathway-based enrichment analysis of GWAS summary data. For this hypothesis-free genome-wide approach, we utilized four commonly used resources (i.e., KEGG, GO, BioCarta, and Reactome) to determine the number of genes included in the GWAS pathway enrichment analyses [40], which could overcome the bias of previous studies for arbitrary gene selection [13, 14]. This analysis revealed 18 significantly enriched pathways that were shared by SCZ and all four smoking phenotypes. These pathways were clustered into postsynaptic density, cadherin binding, dendritic spine, long-term depression, and axon guidance; all of them have been implicated in various psychiatric disorders [11, 13, 39, 43, 48–51]. Compared with the negative controls, the degrees of correlation between pathways across SCZ and smoking phenotypes were much higher, indicating that overlap between SCZ and smoking was nonrandom at any pathway level. These findings strongly indicate that the common pathways identified for both SCZ and smoking behaviors were attributable to the shared genetic vulnerability.

Given that leveraging multi-omics datasets is a better way to understand the molecular mechanism of complex diseases, we here provide robust evidence to explain the underlying mechanism of the comorbidity between SCZ and smoking from genetic, epigenetic, and expression points of view. Based on the gene co-expression data, we found a considerable number of genes in common pathways that showed differential co-expression patterns in different developmental time points and brain regions, providing supportive evidence that shared genetic risk for SCZ and smoking phenotypes has a vital influence on neurodevelopmentally regulated pathways. Similar to a previous study [43], by integrating gene co-expression data with pathway-based analyses on large-scale GWAS, we could identify even greater specificity of candidate genes conferring risk for the comorbidity.

Previous studies [11, 44, 52–54] have shown that aberrant methylated DNAs (DNAm) were involved in the etiology of SCZ and smoking. For example, our previous study, based on a sophisticated data mining of published papers [44], showed that 320 genes exhibited robust methylation evidence of involvement in the etiology of smoking. In the current study, we highlighted 34 genes with significant methylation evidence that were associated with both SCZ and smoking. More interestingly, we found that 23 of these 34 genes were located in a molecular subnetwork, which is consistent with the previous notion that function-related genes may collectively contribute the risk of the pathology of complex diseases [44]. Notably, 16 of the 34 methylated genes have often been reported to be associated with SCZ and smoking. For example, AKT3 is a critical molecule underlying psychiatric-related behaviors, including SCZ [11, 55], cognitive function [56], and smoking behaviors [44]. Multiple lines of evidence from genetic association studies [11, 57, 58] have indicated that *MAD1L1* is significantly associated with SCZ. Based on network-assisted investigation of combined causal signals from GWAS studies [59], *CNA12* showed a significant association with SCZ. *PRKCZ* and *PTPRN2* were reported to be SCZ-associated differential methylation regions [54]. The *KCNQ1* gene belongs to the potassium channel gene family and plays a vital role in signal transduction within the central nervous system. It may contribute to the shared risk of diminished processing speed, diminished white matter integrity, and increased risk of SCZ [60, 61]. In addition, we found that a large proportion of the 34 genes were targeted by various psychotropic drugs, and the expression patterns of these drug-responsive genes were significantly regulated in mouse brain by treatment with 18 major psychotropic drugs in a dose- or time-dependent pattern. Thus, these genes likely represent targets for pharmacotherapeutic intervention in SCZ and smoking behaviors.

Genetic variants influencing DNAm and mRNA expression can modulate gene transcript levels and thereby exert risk effects on diseases, as evidenced by meQTL and eQTL analyses of top GWAS risk-associated SNPs for various human phenotypes, including smoking [54, 62, 63] and SCZ [64, 65]. Thus, we first explored the vital influence of regulatory genomic elements (i.e., *cis*-meQTLs and *cis*-eQTLs) in neurodevelopmental processes of the comorbidity of smoking and SCZ. In human brain samples, we identified 21 of 34 candidate genes with a great number of SNP–CpG pairs. Interestingly, there was a large proportion, 39.4%, of these SNPs that showed allele-specific gene expression. By downloading SNPs from the GWAS Catalog (on 03 May 2019), some of the identified SNPs were found to be associated with psychiatric disorders in GWAS studies. For example, rs1107592 (P = 2.0 × 10^−6^) and rs802568 (P = 2.0 × 10^−7^) were suggested to be associated with bipolar disorder and SCZ [66]. Rs1107592 (P = 5.0 × 10^−6^), rs4721295 (P = 6.0 × 10^−10^), and rs12666575 (P = 2.0 × 10^−9^) were significantly associated with SCZ and other psychiatric disorders, such as autism spectrum, bipolar, and major depressive disorders [67–69]. Rs1403174 (P = 3.0 × 10^−10^) showed significant association with age at smoking initiation [70]. These findings were consistent with earlier reports where it was discovered that the majority of non-coding risk-associated SNPs for brain disorders influence gene expression via DNAm [71, 72].

Some limitations of the current study warrant comment. First, those SCZ- and smoking-related genes identified were prioritized from GWAS. In view of the inherent defects of the GWAS approach, some of these genes might not be truly associated with both of the disorders. Second, a great number of genes have not been characterized or mapped to computationally predicted or manually curated pathways. Thus, the contributions of these genes could not be delineated in pathway enrichment analysis. Third, although we have provided very strong evidence from genetic association, gene expression, and DNA methylation to support the 18 common pathways as being linked to both smoking and SCZ, we could not determine to what extent each specific pathway contributes to smoking or SCZ, nor could we quantify the relative influences of each pathway for the two disorders.

In sum, by employing a comprehensive bioinformatics analysis of genomic and pharmacogenomics data at the DNA, methylation, or expression levels, we first identified 18 biological pathways that are significantly associated with both SCZ and smoking phenotypes. Subsequently, we discovered 34 novel and promising susceptibility genes and variants within these genes with robust genetic, epigenetic, expression, and pharmacological evidence for both SCZ and smoking. Our findings lend considerable weight to the hypothesis that shared genetic vulnerabilities create a propensity for the comorbidity of SCZ and smoking. By pinpointing which and how SNPs in these candidate genes affect these disorders, this study provides novel insights into the biological mechanism and a solid foundation for understanding the shared disease biology of SCZ and smoking behaviors.

## MATERIALS AND METHODS

### Multi-omics datasets used in current study

In current investigation, we performed a series of multi-omics data analyses, which include large-scale meta-GWAS, BrainSpan exon array data on brain development and aging, methylation data, expression data, and pharmacogenomics data. The following is a brief summary of datasets used in our analysis. For details on these datasets, please infer to Supplementary Table 1.

1. GWAS data on SCZ: This dataset was obtained from a published GWAS [11] on SCZ from the Psychiatric Genomics Consortium (PGC), of which the data from case-control samples (N = 79,845) were used for both gene and pathway analysis.
2. GWAS data on smoking: This dataset was from a published meta-GWAS on smoking behaviors (including smoking status, quantity smoked in ever-regular smokers, smoking cessation, and age at initiation) by the Tobacco and Genetics Consortium (TAG), which contained the genotype data for a total sample size of 74,053 [12].
3. GWAS data on height and null: To demonstrate that the identified pathways were attributable to shared biology between SCZ and smoking, we performed the GWAS enrichment analysis on two other independent data sets as negative controls. One of them was a GWAS of human height with a sample size of 183,727 [73], and the other was a null GWAS based on randomly distributed phenotypes that we constructed from a real GWAS with a sample size of 3,960 [74].
4. Expression data on brain development and aging: The BrainSpan exon array data related to brain development and aging were downloaded from the NCBI’s Gene Expression Omnibus (GEO; Accession No. GSE25219), with a sample size of 1,340.
5. Methylation data on smoking and SCZ: Three datasets were used for this part of the analysis, with the first one from our previous study on smoking and consisting of 18,677 samples [44], the second one on 847 SCZ blood samples from NCBI GEO (Accession No. GSE84727) [75], and the third one on brain samples (N=258) from NCBI GEO (Accession No. GSE74193) [76].
6. Expression data on smoking and SCZ: Two expression datasets were used here, with the first one based on olfactory epithelium tissues (N = 31) and the second one on induced pluripotent human stem cells (hiPSCs; N = 8). Both were downloaded from NCBI GEO (Accession Nos. GSE73129 and GSE25673).
7. Pharmacogenomics data: To reveal potential druggable genes with therapeutic effects, we downloaded the psychotropic drug-treated gene expression data from NCBI’s GEO with Accession Nos. of GSE45229, GES50254, GSE48954, GSE15774, and GSE48951. We first explored the dosage influence of quetiapine and nicotine on gene expression changes in mouse striatum, then concentrated on the time-course (1, 2, 4, and 8 hours) of gene expression alterations in mouse striatum that were induced by 18 major psychotropic drugs.

### Pathway- and gene-based analysis

We combined gene-set data from four sources: KEGG [77], GO [78], BioCarta [79], and Reactome [80], which were downloaded from their respective sources on or before May 12, 2017. Because of concerns that the gene set with > 300 genes or < 10 genes is considered to be either less specific and computationally inefficient or over-dispersed [40], we confined our analysis to those pathways with 10–300 genes.

We used the GSA-SNP program [81] to perform GWAS-based enrichment analysis. The SNPs of interest were assigned to genes if they lie within 20 kb upstream or downstream of the gene, and each SNP was assigned to only one gene. When multiple SNPs were mapped to the same gene, the GSA-SNP chose the most significantly associated SNP. Additionally, we used the MAGMA (https://ctg.cncr.nl/software/magma) for gene-based analysis of GWAS summary data. Because LD could result in a number of genes rather than one being counted as significant when genes are physically close in one region, we used the LD-pruned method to calculate the LD for the published GWAS data with the 1000 Genome European Phase 3 panel as the reference.

### Computer permutation analysis

There were 3,334 genes (named Gene set 1) from 84 common pathways with a *q* value < 0.1 from our pathway enrichment analysis. To determine whether these genes were indeed significantly overrepresented with identified genes from our gene-based analysis, we conducted permutation analysis by randomly selecting 3,334 genes in the 84 common pathways from the total genes (N = 17,385) in all 2,532 pathways for 10^6^ times. We then calculated how many times the counts of genes overlapped with the genes from gene-based analysis that were larger than the observed number among 10^6^ trials. The probability of the observation was treated as the *P*-value, with a P value < 0.05 being considered significant.

### Co-expression network analysis

We then conducted further analysis for 3,334 identified genes in Gene Set 1 to determine how these pathways were related to brain development and aging with the BrainSpan exon array data. RNA expression profiles for 1,340 samples were analyzed by using weighted gene co-expression network analysis (WGCNA), an R package used for clustering genes into modules according to co-expression data. The 10 most highly connected genes within each module were displayed in the network plot by using Cytoscape (v. 3.5.1) (http://www.cytoscape.org/).

### Smoking- and SCZ-associated differentially methylated loci and regions

To determine whether the identified genes from co-expression modules were involved in the smoking- and SCZ-associated methylation process, we first selected a list of 1,429 smoking-associated methylated genes from our previous study [44], and then examined whether these smoking-associated methylation module genes were significantly methylated in SCZ patients [75]. Bonferroni correction was used to determine significant association. An exact binomial analysis was applied to examine whether there existed a significant excess of consistence of observed methylated genes between smoking and SCZ more often than was expected by chance.

### Candidate gene expression profiles in SCZ and smoking

We performed an ANOVA analysis to explore the differential expression profiles of the 34 genes in olfactory epithelium tissues among SCZ patients and controls grouped by smoking status. Turkey HSD test was used for multiple comparisons. Considering that human induced pluripotent stem cells (hiPSCs) provide a novel strategy for defining characteristics of schizophrenic neurons, we further used the first cell-based human model of SCZ by directly reprogramming fibroblasts from schizophrenic patients into hiPSCs and subsequently differentiating these disorder-specific hiPSCs into neurons *in vitro* to explore the different expression profiles of 34 genes between controls and schizophrenic patients.

### Candidate genes in response to various psychotropic drugs

To identify potential druggable targets, we searched for these identified genes in the Drug-Gene Interaction database (DGIdb) (v. 3.0; http://www.dgidb.org/). Firstly, we searched the 20 databases for drug–gene interactions with FDA-approved pharmaceutical compounds according to 51 known interaction types with 34 common genes for both SCZ and smoking phenotypes. Secondly, we searched 10 databases for the potential drug ability for gene targets to reveal genes that might form targets for novel therapies in addition to existing medicines. To explore whether these genes had therapeutic effects, we applied the dosage treatment of quetiapine and nicotine, and the time-course (1, 2, 4, and 8 hours) treatment of 18 major psychotropic drugs on gene expression changes in mouse striatum. Furthermore, using the computer permutation analysis of 10^5^ times, we determined whether these 34 candidate genes were more prone to drug-induced action than other 1,588 genes in 18 common pathways, 3,334 genes in 84 common pathways, as well as more than 30,000 background genes.

### Cis-meQTLs/eQTLs of SNPs within 34 genes

To explore the relations between genotype and methylation status, we conducted cis-meQTL analysis of SNPs within 34 candidate genes. Based on the two large GWAS used in our pathway-based analysis, we collected suggested SNPs with a P value of < 0.05 in 34 candidate genes for SCZ and smoking behaviors. Considering that multiple lines of evidence have suggested that identified tag SNPs were more likely to be in LD with casual variants [82], we generated a list of SNPs that were in strong LD (LD cutoff r^2^≥0.5) with each tag SNP using the 1000 Genome European Phase 3 panel for reference genotyping.

By employing these identified SNPs, we downloaded *cis*-meQTL data from human brain samples (N = 258) [76] and used the Matrix eQTL (v. 2.1.1) R package [83] to examine the associations between SNPs and methylation loci with linear regression under an additive model. We restricted methylation loci to 20 kb upstream and downstream of each SNP. The intervals for nearby SNPs were combined if they overlapped. There were 7,426,085 common genotyped and imputed SNPs from the 1,000 Genomes reference panel and 477,636 qualified CpGs used for *cis*-meQTL analysis with a maximum distance of 20 kb between each SNP and CpG analyzed, resulting in 47,675,913 tests. A total of 4,107,214 significant SNP-CpG methylation associations at FDR < 0.01 were identified from this dataset. To further explore the *cis*-regulatory effects of SNPs on expression of the 34 candidate genes, we performed a *cis*-acting eQTL analysis in human tissues by using a web-based tool of GTEX PROTAL (https://gtexportal.org/home/).

## Supplementary Material

Supplementary References

Supplementary Figures

Supplementary Table 1

Supplementary Table 2

Supplementary Table 3

Supplementary Tables 4, 6, 7

Supplementary Table 5
