# Supplementary material for: Identification of 34 genes conferring genetic and pharmacological risk for the comorbidity of schizophrenia and smoking behaviors
Source: Aging (Albany NY). 2020 Feb 3;12(3):2169–225. doi: 10.18632/aging.102735 (PMC7041787; doi:10.18632/aging.102735)
Supplement: Supplementary References [file aging-12-102735-s005..pdf]

SUPPLEMENTARY REFERENCES

1. Schizophrenia Working Group of the Psychiatric Genomics Consortium. Biological insights from 108 schizophrenia-associated genetic loci. Nature. 2014; 511:421–27. https://doi.org/10.1038/nature13595 PMID:25056061

2. Tobacco and Genetics Consortium. Genome-wide meta-analyses identify multiple loci associated with smoking behavior. Nat Genet. 2010; 42:441–47. https://doi.org/10.1038/ng.571 PMID:20418890

3. Lango Allen H, Estrada K, Lettre G, Berndt SI, Weedon MN, Rivadeneira F, Willer CJ, Jackson AU, Vedantam S, Raychaudhuri S, Ferreira T, Wood AR, Weyant RJ, et al. Hundreds of variants clustered in genomic loci and biological pathways affect human height. Nature. 2010; 467:832–38. https://doi.org/10.1038/nature09410 PMID:20881960

4. Landi MT, Chatterjee N, Yu K, Goldin LR, Goldstein AM, Rotunno M, Mirabello L, Jacobs K, Wheeler W, Yeager M, Bergen AW, Li Q, Consonni D, et al. A genome-wide association study of lung cancer identifies a region of chromosome 5p15 associated with risk for adenocarcinoma. Am J Hum Genet. 2009; 85:679–91. https://doi.org/10.1016/j.ajhg.2009.09.012 PMID:19836008

5. Ma Y, Li MD. Establishment of a Strong Link Between Smoking and Cancer Pathogenesis through DNA Methylation Analysis. Sci Rep. 2017; 7:1811. https://doi.org/10.1038/s41598-017-01856-4 PMID:28500316

6. Hannon E, Dempster E, Viana J, Burrage J, Smith AR, Macdonald R, St Clair D, Mustard C, Breen G, Therman S, Kaprio J, Toulopoulou T, Hulshoff Pol HE, et al. An integrated genetic-epigenetic analysis of schizophrenia: evidence for co-localization of genetic associations and differential DNA methylation. Genome Biol. 2016; 17:176. https://doi.org/10.1186/s13059-016-1041-x PMID:27572077

7. Jaffe AE, Gao Y, Deep-Soboslay A, Tao R, Hyde TM, Weinberger DR, Kleinman JE. Mapping DNA methylation across development, genotype and schizophrenia in the human frontal cortex. Nat Neurosci. 2016; 19:40–47. https://doi.org/10.1038/nn.4181 PMID:26619358

8. Horiuchi Y, Kondo MA, Okada K, Takayanagi Y, Tanaka T, Ho T, Varvaris M, Tajinda K, Hiyama H, Ni K, Colantuoni C, Schretlen D, Cascella NG, et al. Molecular signatures associated with cognitive deficits in schizophrenia: a study of biopsied olfactory neural epithelium. Transl Psychiatry. 2016; 6:e915. https://doi.org/10.1038/tp.2016.154 PMID:27727244

9. Horiuchi Y, Kano S, Ishizuka K, Cascella NG, Ishii S, Talbot CC Jr, Jaffe AE, Okano H, Pevsner J, Colantuoni C, Sawa A. Olfactory cells via nasal biopsy reflect the developing brain in gene expression profiles: utility and limitation of the surrogate tissues in research for brain disorders. Neurosci Res. 2013; 77:247–50. https://doi.org/10.1016/j.neures.2013.09.010 PMID:24120685

10. Cascella NG, Takaki M, Lin S, Sawa A. Neurodevelopmental involvement in schizophrenia: the olfactory epithelium as an alternative model for research. J Neurochem. 2007; 102:587–94. https://doi.org/10.1111/j.1471-4159.2007.04628.x PMID:17488280

11. Brennand KJ, Simone A, Jou J, Gelboin-Burkhart C, Tran N, Sangar S, Li Y, Mu Y, Chen G, Yu D, McCarthy S, Sebat J, Gage FH. Modelling schizophrenia using human induced pluripotent stem cells. Nature. 2011; 473:221–25. https://doi.org/10.1038/nature09915 PMID:21490598

12. Kondo MA, Tajinda K, Colantuoni C, Hiyama H, Seshadri S, Huang B, Pou S, Furukori K, Hookway C, Jaaro-Peled H, Kano SI, Matsuoka N, Harada K, et al. Unique pharmacological actions of atypical neuroleptic quetiapine: possible role in cell cycle/fate control. Transl Psychiatry. 2013; 3:e243. https://doi.org/10.1038/tp.2013.19 PMID:23549417

13. Kogel U, Schlage WK, Martin F, Xiang Y, Ansari S, Leroy P, Vanscheeuwijck P, Gebel S, Buettner A, Wyss C, Esposito M, Hoeng J, Peitsch MC. A 28-day rat inhalation study with an integrated molecular toxicology endpoint demonstrates reduced exposure effects for a prototypic modified risk tobacco product compared with conventional cigarettes. Food Chem Toxicol. 2014; 68:204–17. https://doi.org/10.1016/j.fct.2014.02.034 PMID:24632068

14. Howell KR, Floyd K, Law AJ. PKBγ/AKT3 loss-of-function causes learning and memory deficits and deregulation of AKT/mTORC2 signaling: relevance for schizophrenia. PLoS One. 2017; 12:e0175993. https://doi.org/10.1371/journal.pone.0175993 PMID:28467426

15. Levenga J, Wong H, Milstead RA, Keller BN, LaPlante LE, Hoeffer CA. AKT isoforms have distinct hippocampal expression and roles in synaptic plasticity. eLife. 2017; 6:6. https://doi.org/10.7554/eLife.30640 PMID:29173281

16. Ripke S, O'Dushlaine C, Chambert K, Moran JL, Kähler AK, Akterin S, Bergen SE, Collins AL, Crowley JJ, Fromer M, Kim Y, Lee SH, Magnusson PK, et al, and Multicenter

Genetic Studies of Schizophrenia Consortium, and Psychosis Endophenotypes International Consortium, and Wellcome Trust Case Control Consortium 2. Genome-wide association analysis identifies 13 new risk loci for schizophrenia. Nat Genet. 2013; 45:1150–59. https://doi.org/10.1038/ng.2742 PMID:23974872

17. Network and Pathway Analysis Subgroup of Psychiatric Genomics Consortium. Psychiatric genome-wide association study analyses implicate neuronal, immune and histone pathways. Nat Neurosci. 2015; 18:199–209. https://doi.org/10.1038/nn.3922 PMID:25599223

18. Sharp SI, Hu Y, Weymer JF, Rizig M, McQuillin A, Hunt SP, Gurling HM. The effect of clozapine on mRNA expression for genes encoding G protein-coupled receptors and the protein components of clathrin-mediated endocytosis. Psychiatr Genet. 2013; 23:153–62. https://doi.org/10.1097/YPG.0b013e32835fe51d PMID:23811784

19. Genis-Mendoza A, Gallegos-Silva I, Tovilla-Zarate CA, López-Narvaez L, González-Castro TB, Hernández-Díaz Y, López-Casamichana M, Nicolini H, Morales-Mulia S. Comparative Analysis of Gene Expression Profiles Involved in Calcium Signaling Pathways Using the NLVH Animal Model of Schizophrenia. J Mol Neurosci. 2018; 64:111–16. https://doi.org/10.1007/s12031-017-1013-y PMID:29214423

20. Guan F, Li L, Qiao C, Chen G, Yan T, Li T, Zhang T, Liu X. Evaluation of genetic susceptibility of common variants in CACNA1D with schizophrenia in Han Chinese. Sci Rep. 2015; 5:12935. https://doi.org/10.1038/srep12935 PMID:26255836

21. Berger SM, Bartsch D. The role of L-type voltage-gated calcium channels Cav1.2 and Cav1.3 in normal and pathological brain function. Cell Tissue Res. 2014; 357:463–76. https://doi.org/10.1007/s00441-014-1936-3 PMID:24996399

22. Gao R, Piguel NH, Melendez-Zaidi AE, Martin-de-Saavedra MD, Yoon S, Forrest MP, Myczek K, Zhang G, Russell TA, Csernansky JG, Surmeier DJ, Penzes P. CNTNAP2 stabilizes interneuron dendritic arbors through CASK. Mol Psychiatry. 2018; 23:1832–50. https://doi.org/10.1038/s41380-018-0027-3 PMID:29610457

23. Poot M. Connecting the CNTNAP2 Networks with Neurodevelopmental Disorders. Mol Syndromol. 2015; 6:7–22. https://doi.org/10.1159/000371594 PMID:25852443

24. Ji W, Li T, Pan Y, Tao H, Ju K, Wen Z, Fu Y, An Z, Zhao Q, Wang T, He L, Feng G, Yi Q, Shi Y. CNTNAP2 is significantly associated with schizophrenia and major depression in the Han Chinese population. Psychiatry Res. 2013; 207:225–28. https://doi.org/10.1016/j.psychres.2012.09.024 PMID:23123147

25. Jia P, Wang L, Fanous AH, Pato CN, Edwards TL, Zhao Z, and International Schizophrenia Consortium. Network-assisted investigation of combined causal signals from genome-wide association studies in schizophrenia. PLOS Comput Biol. 2012; 8:e1002587. https://doi.org/10.1371/journal.pcbi.1002587 PMID:22792057

26. Solismaa A, Kampman O, Lyytikäinen LP, Seppälä N, Viikki M, Mononen N, Lehtimäki T, Leinonen E. Histaminergic gene polymorphisms associated with sedation in clozapine-treated patients. Eur Neuropsychopharmacol. 2017; 27:442–49. https://doi.org/10.1016/j.euroneuro.2017.03.009 PMID:28400155

27. Tiwari AK, Zhang D, Pouget JG, Zai CC, Chowdhury NI, Brandl EJ, Qin L, Freeman N, Lieberman JA, Meltzer HY, Kennedy JL, Müller DJ. Impact of histamine receptors H1 and H3 polymorphisms on antipsychotic-induced weight gain. World J Biol Psychiatry. 2018; 19:S97–S105. https://doi.org/10.1080/15622975.2016.1262061 PMID:27855565

28. Rannals MD, Hamersky GR, Page SC, Campbell MN, Briley A, Gallo RA, Phan BN, Hyde TM, Kleinman JE, Shin JH, Jaffe AE, Weinberger DR, Maher BJ. Psychiatric Risk Gene Transcription Factor 4 Regulates Intrinsic Excitability of Prefrontal Neurons via Repression of SCN10a and KCNQ1. Neuron. 2016; 90:43–55. https://doi.org/10.1016/j.neuron.2016.02.021 PMID:26971948

29. Bruce HA, Kochunov P, Paciga SA, Hyde CL, Chen X, Xie Z, Zhang B, Xi HS, O'Donnell P, Whelan C, Schubert CR, Bellon A, Ament SA, et al. Potassium channel gene associations with joint processing speed and white matter impairments in schizophrenia. Genes Brain Behav. 2017; 16:515–21. https://doi.org/10.1111/gbb.12372 PMID:28188958

30. Chang S, Fang K, Zhang K, Wang J, and Network-Based Analysis of Schizophrenia Genome-Wide Association Data to Detect the Joint Functional Association Signals. Network-Based Analysis of Schizophrenia Genome-Wide Association Data to Detect the Joint Functional Association Signals. PLoS One. 2015; 10:e0133404. https://doi.org/10.1371/journal.pone.0133404 PMID:26193471

31. Su L, Shen T, Huang G, Long J, Fan J, Ling W, Jiang J. Genetic association of GWAS-supported MAD1L1 gene

polymorphism rs12666575 with schizophrenia susceptibility in a Chinese population. Neurosci Lett. 2016; 610:98–103.
https://doi.org/10.1016/j.neulet.2015.10.061
PMID:26528791

32. Candemir E, Kollert L, Weißflog L, Geis M, Müller A, Post AM, O'Leary A, Harro J, Reif A, Freudenberg F. Interaction of NOS1AP with the NOS-I PDZ domain: implications for schizophrenia-related alterations in dendritic morphology. Eur Neuropsychopharmacol. 2016; 26:741–55. https://doi.org/10.1016/j.euroneuro.2016.01.008 PMID:26861996

33. Carrel D, Hernandez K, Kwon M, Mau C, Trivedi MP, Brzustowicz LM, Firestein BL. Nitric oxide synthase 1 adaptor protein, a protein implicated in schizophrenia, controls radial migration of cortical neurons. Biol Psychiatry. 2015; 77:969–78. https://doi.org/10.1016/j.biopsych.2014.10.016 PMID:25542305

34. Hadzimichalis NM, Previtera ML, Moreau MP, Li B, Lee GH, Dulencin AM, Matteson PG, Buyske S, Millonig JH, Brzustowicz LM, Firestein BL. NOS1AP protein levels are altered in BA46 and cerebellum of patients with schizophrenia. Schizophr Res. 2010; 124:248–50. https://doi.org/10.1016/j.schres.2010.05.009 PMID:20605702

35. Xu B, Wratten N, Charych EI, Buyske S, Firestein BL, Brzustowicz LM. Increased expression in dorsolateral prefrontal cortex of CAPON in schizophrenia and bipolar disorder. PLoS Med. 2005; 2:e263. https://doi.org/10.1371/journal.pmed.0020263 PMID:16146415

36. Brzustowicz LM, Simone J, Mohseni P, Hayter JE, Hodgkinson KA, Chow EW, Bassett AS. Linkage disequilibrium mapping of schizophrenia susceptibility to the CAPON region of chromosome 1q22. Am J Hum Genet. 2004; 74:1057–63. https://doi.org/10.1086/420774 PMID:15065015

37. Rosa A, Fañanás L, Cuesta MJ, Peralta V, Sham P. 1q21-q22 locus is associated with susceptibility to the reality-distortion syndrome of schizophrenia spectrum disorders. Am J Med Genet. 2002; 114:516–18. https://doi.org/10.1002/ajmg.10526 PMID:12116186

38. Kremeyer B, García J, Kymäläinen H, Wratten N, Restrepo G, Palacio C, Miranda AL, López C, Restrepo M, Bedoya G, Brzustowicz LM, Ospina-Duque J, Arbeláez MP, Ruiz-Linares A. Evidence for a role of the NOS1AP (CAPON) gene in schizophrenia and its clinical dimensions: an association study in a South American population isolate. Hum Hered. 2009; 67:163–73. https://doi.org/10.1159/000181154 PMID:19077434

39. Wang HN, Liu GH, Zhang RG, Xue F, Wu D, Chen YC, Peng Y, Peng ZW, Tan QR. Quetiapine Ameliorates Schizophrenia-Like Behaviors and Protects Myelin Integrity in Cuprizone Intoxicated Mice: The Involvement of Notch Signaling Pathway. Int J Neuropsychopharmacol. 2015; 19:pyv088. https://doi.org/10.1093/ijnp/pyv088 PMID:26232790

40. Kim SK, Lee JY, Park HJ, Kim JW, Chung JH. Association study between polymorphisms of the PARD3 gene and schizophrenia. Exp Ther Med. 2012; 3:881–85. https://doi.org/10.3892/etm.2012.496 PMID:22969987

41. Guipponi M, Santoni FA, Setola V, Gehrig C, Rotharmel M, Cuenca M, Guillin O, Dikeos D, Georgantopoulos G, Papadimitriou G, Curtis L, Méary A, Schürhoff F, et al. Exome sequencing in 53 sporadic cases of schizophrenia identifies 18 putative candidate genes. PLoS One. 2014; 9:e112745. https://doi.org/10.1371/journal.pone.0112745 PMID:25420024

42. Goldsmith DR, Haroon E, Miller AH, Strauss GP, Buckley PF, Miller BJ. TNF-α and IL-6 are associated with the deficit syndrome and negative symptoms in patients with chronic schizophrenia. Schizophr Res. 2018; 199:281–84. https://doi.org/10.1016/j.schres.2018.02.048 PMID:29499967

43. Mostaid MS, Pantelis C, Everall IP, Bousman CA. Decreased peripheral TNF alpha (TNF-α) mRNA expression in patients with treatment-resistant schizophrenia. Schizophr Res. 2018; 202:387–88. https://doi.org/10.1016/j.schres.2018.04.032 PMID:29706448

44. Chen SY, Huang PH, Cheng HJ. Disrupted-in-Schizophrenia 1-mediated axon guidance involves TRIO-RAC-PAK small GTPase pathway signaling. Proc Natl Acad Sci USA. 2011; 108:5861–66. https://doi.org/10.1073/pnas.1018128108 PMID:21422296
