# Supplementary material for: Identification of 34 genes conferring genetic and pharmacological risk for the comorbidity of schizophrenia and smoking behaviors
Source: Aging (Albany NY). 2020 Feb 3;12(3):2169–225. doi: 10.18632/aging.102735 (PMC7041787; doi:10.18632/aging.102735)
Supplement: Supplementary Figures [file aging-12-102735-s007..pdf]

SUPPLEMENTARY FIGURES

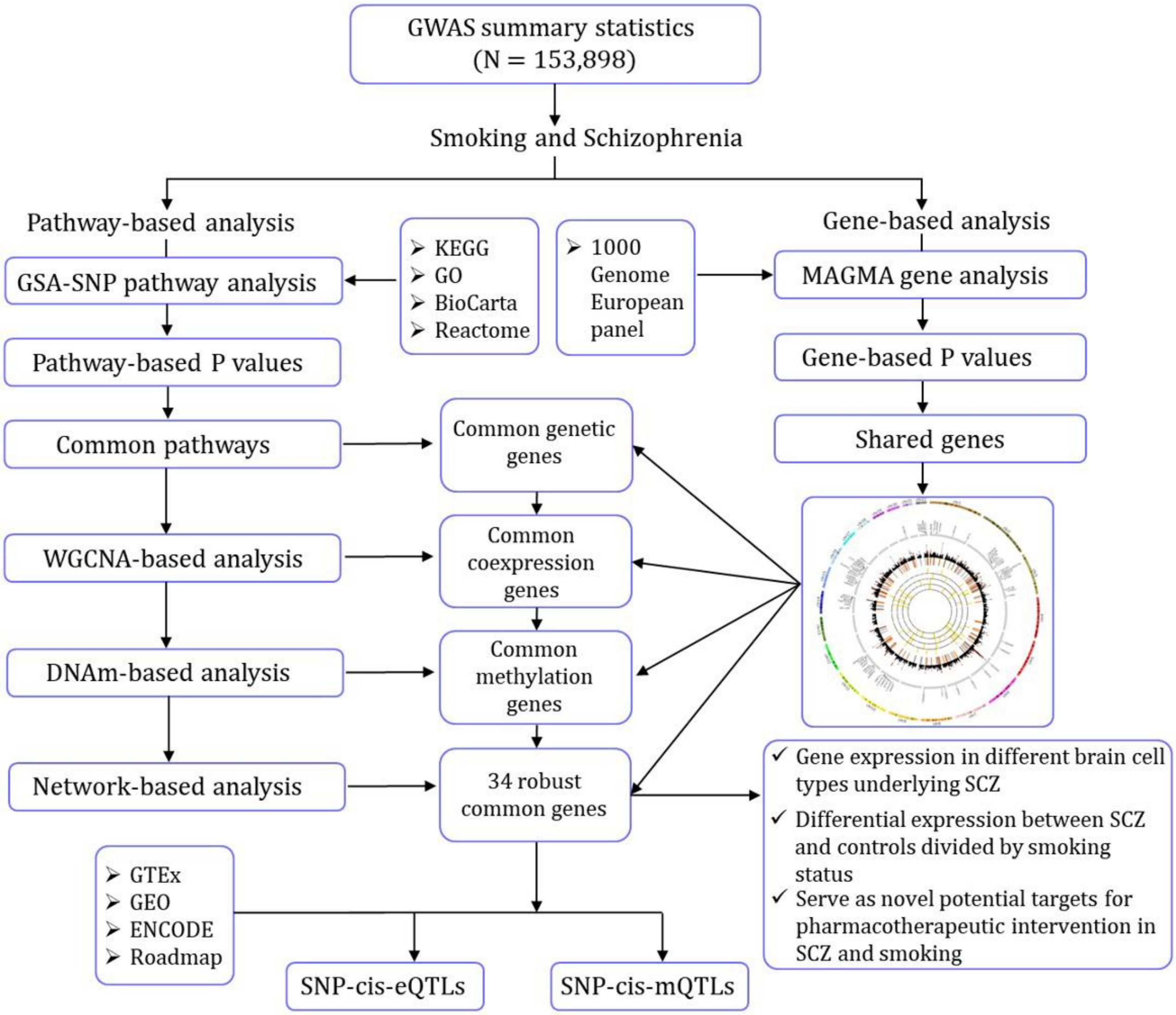

Supplementary Figure 1. Overview of statistical approaches for integrative pathway- and gene-based analysis of GWAS summary statistics. Using GSA-SNP and MAGMA software, pathway- and gene-based analyses were carried out. A number of novel genes were identified from MAGMA analysis. There was a total of 208 shared genes between SCZ and smoking behaviors. According to pathway-based enrichment analysis, 18 common pathways were identified. We performed a simulation analysis and found these 208 genes to be significantly enriched in common pathways identified from GSA-SNP pathway analysis. Further, to explore the underlying biological mechanism of the comorbidity of SCZ and smoking, we then integrated multi-dimensional omics datasets, including human brain and blood transcriptome data, on smoking and SCZ with well-established tools for interrogating such datasets. Finally, we identified 34 candidate genes conferring risk of the comorbidity of SCZ and smoking and revealed the regulatory mechanism of SNP-methylation expression of the comorbidity.

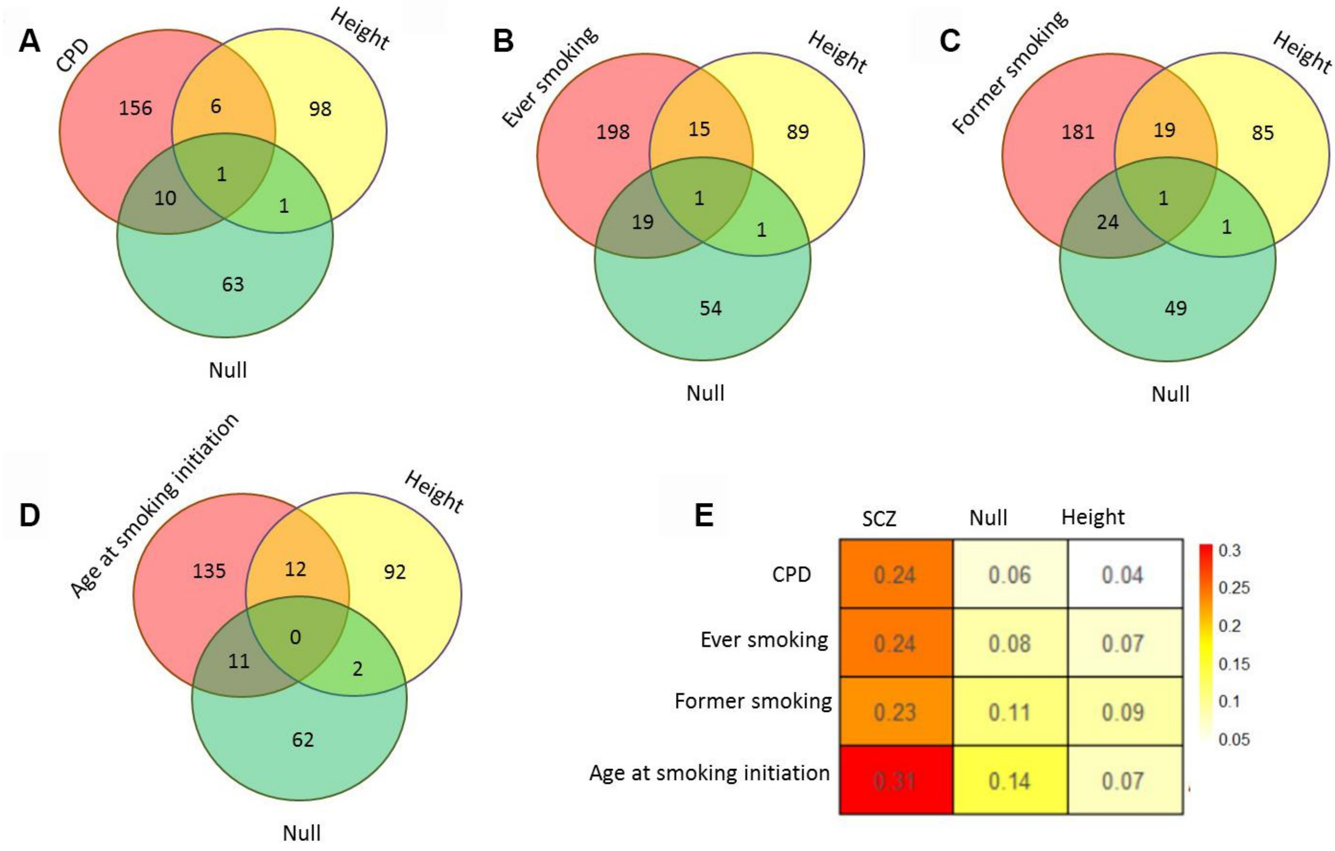

Supplementary Figure 2. Overlapped pathways among smoking behaviors, null, and height. (**A**) Overlapped pathways among CPD, null, and height; (**B**) Overlapped pathways among ever-smoking, null, and height; (**C**) Overlapped pathways among former smoking, null, and height; (**D**) Overlapped pathways among age at smoking initiation, null, and height; (**E**) Heatmap of the proportion of significantly enriched pathways in CPD, ever smoking, former smoking, and age at smoking initiation with SCZ, null, and height. Heatmap plots were generated using the *pheatmap* package in R.

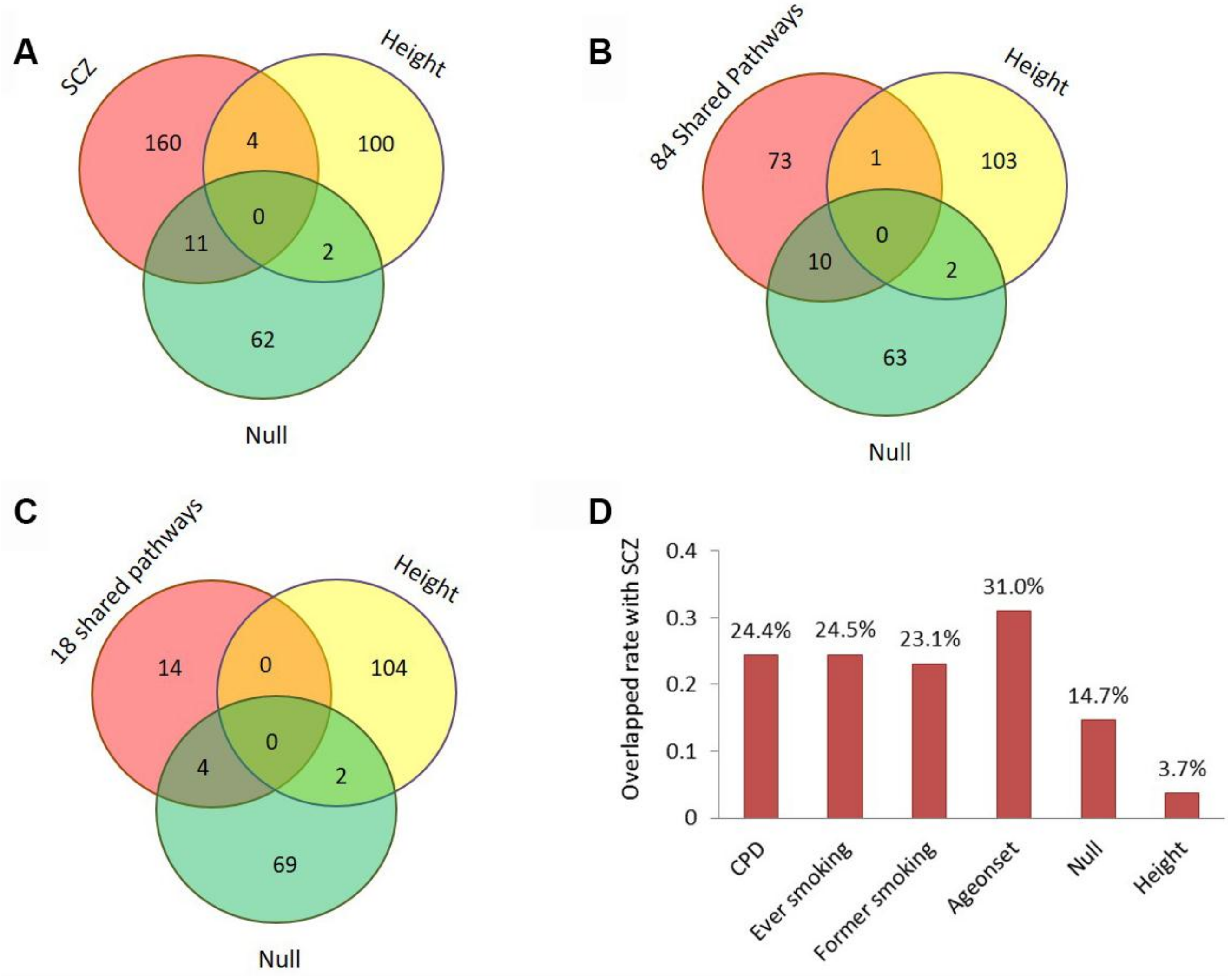

Supplementary Figure 3. Overlapped pathways among SCZ and smoking behaviors, null, and height. (**A**) Venn diagram of significantly enriched pathways for SCZ with the pathways for null and height. (**B**) Venn diagram of significantly enriched pathways among 84 shared pathways between SCZ and at least one of four smoking phenotypes with the pathways for null and height. (**C**) Venn diagram of significantly enriched pathways among 18 pathways shared by SCZ and all four smoking phenotypes with the pathways for null and height. (**D**) The proportion of significantly enriched pathways in CPD, ever smoking, former smoking, age at smoking initiation, null, and height with SCZ.

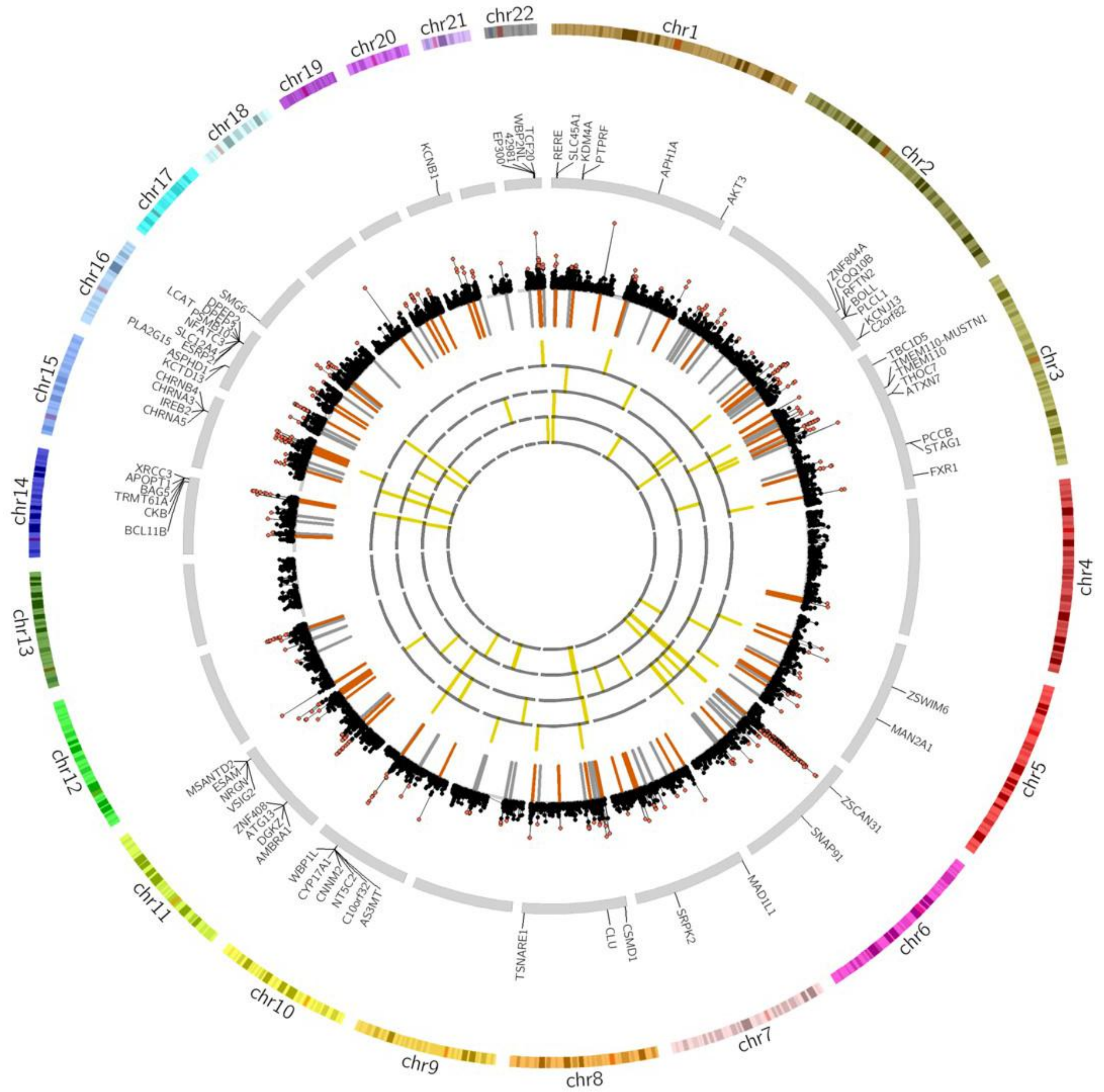

Supplementary Figure 4. Circus plot of results of gene-based analysis of SCZ and smoking phenotypes. The outer ring shows the 22 autosomal human chromosomes. Gene symbols marked in the second ring are the 70 of 208 common genes located in the previously reported 108 loci for SCZ. The third ring demonstrates the results of gene-based analysis of SCZ and 590 genes significantly associated with it ($P < 2.73 \times 10^{-6}$), which are marked with orange points. The orange bars represent 236 genes located in 108 previously identified SCZ-related loci. The grey bars mark novel genes associated with SCZ in the current study. The fourth through the seventh rings mark CPD, ever smoking, former smoking, and smoking initiation, respectively. The yellow bars indicate the distribution of the 208 genes shared by SCZ and smoking.

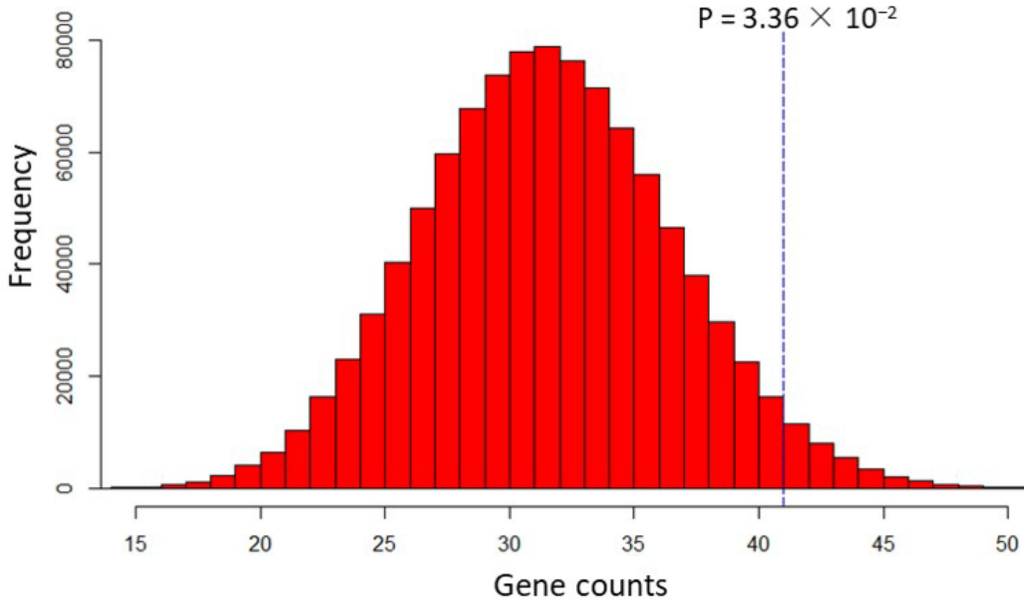

Supplementary Figure 5. Computer permutation analysis of 208 genes associated with SCZ and smoking behaviors for genes in 84 shared pathways.

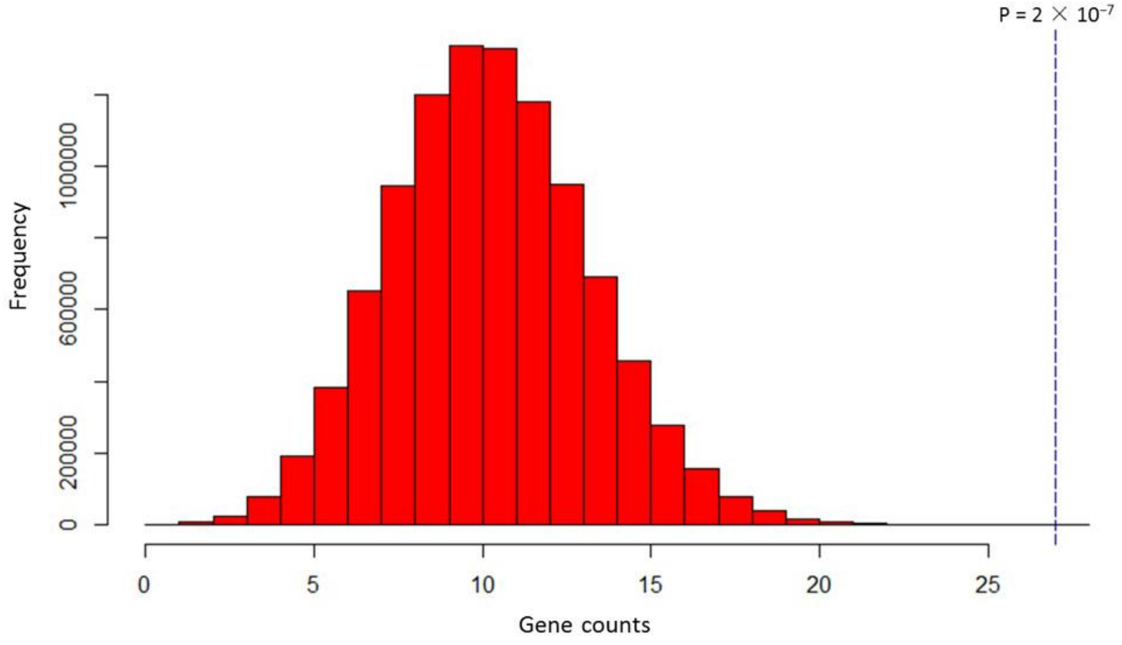

Supplementary Figure 6. Computer permutation analysis of 70 genes associated with SCZ and smoking behaviors for genes in 84 shared pathways.

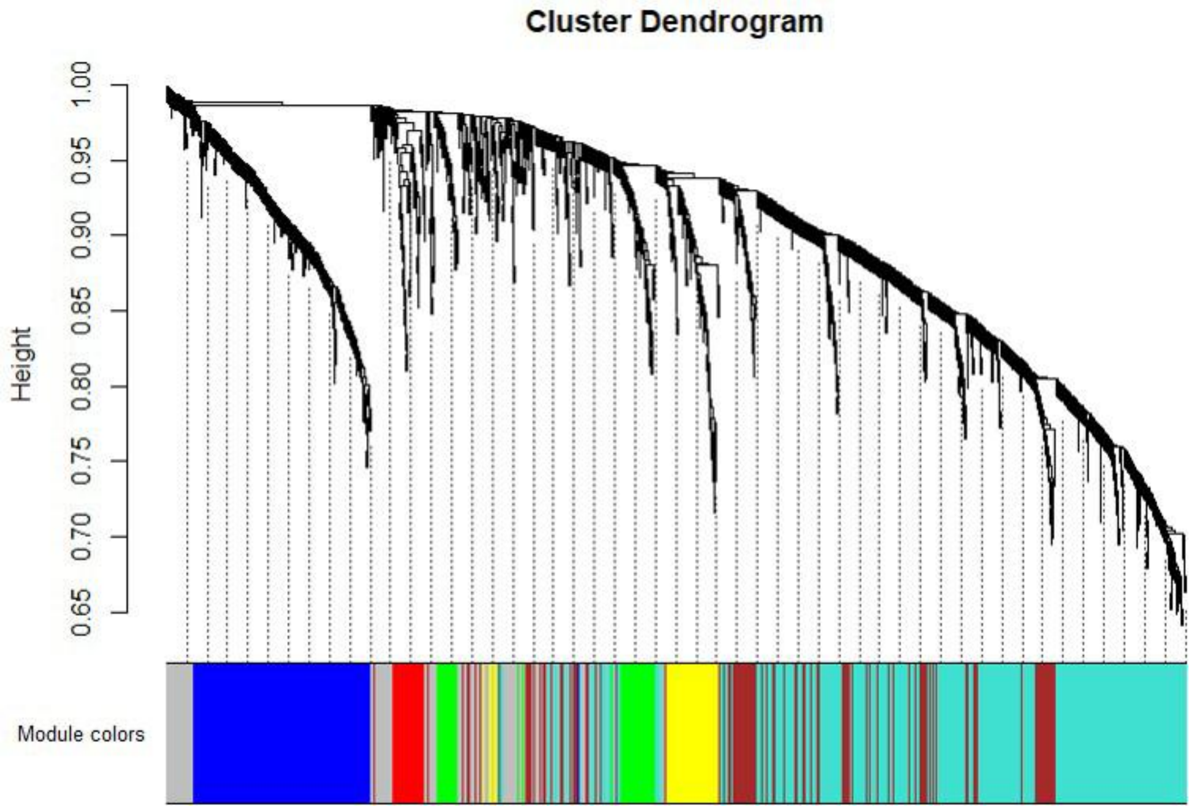

Supplementary Figure 7. WGCNA module-based analysis for genes in all shared pathways with *q* value < 0.1. The hierarchical clustering dendrogram is for all 1,644 expressed and highly connected genes. Each line represents an individual gene. Genes were clustered on the basis of a dissimilarity measure (1-TOM). The branches are related to modules of highly interconnected gene groups. Below the dendrogram of each gene group is a color to indicate the six module colors (i.e., blue, brown, green, red, turquoise, and yellow). Genes within grey boxes were not assigned to a module.

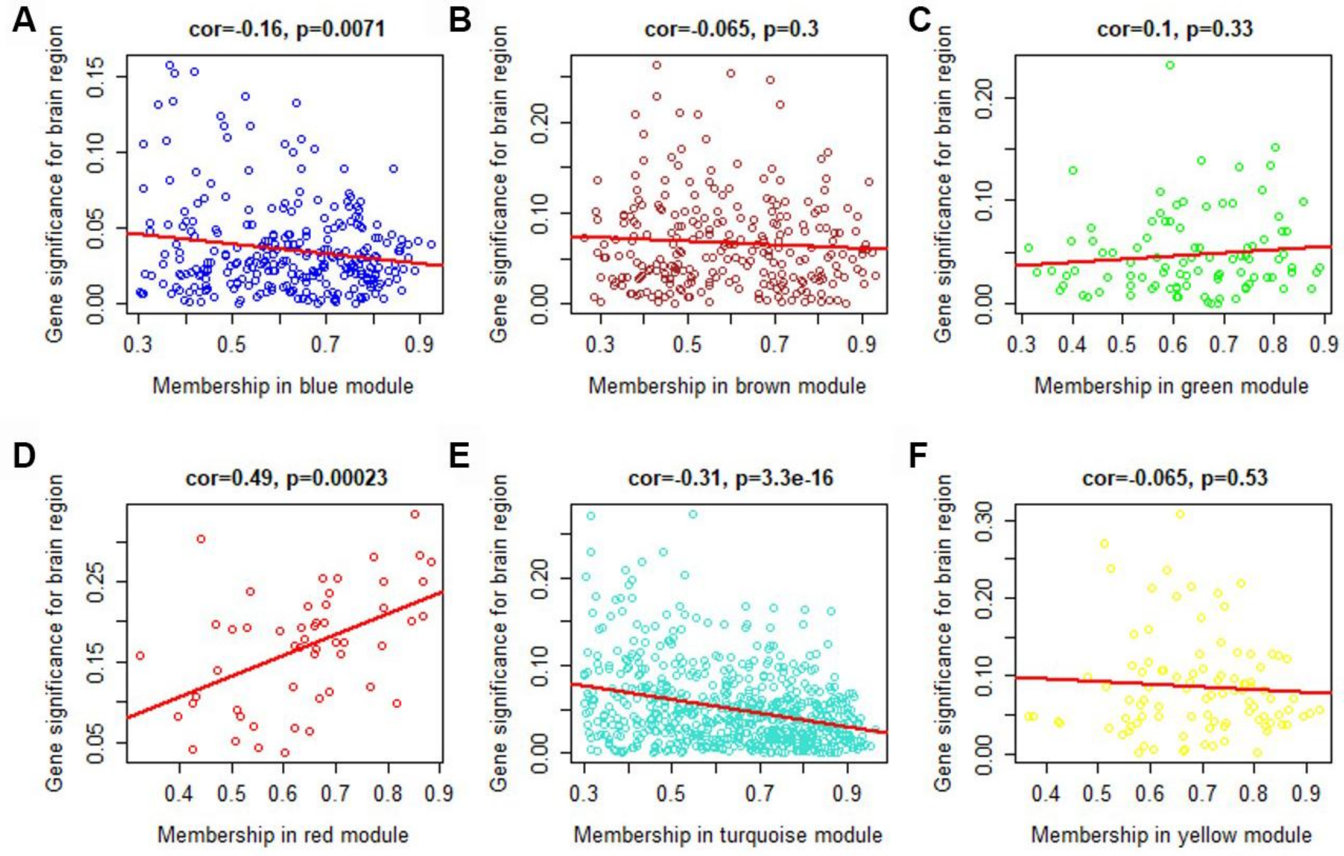

Supplementary Figure 8. Scatterplot of gene significance for brain regions (y axis) vs. module membership (x axis) in different modules. (**A**) For blue module; (**B**) for brown module; (**C**) for green module; (**D**) for red module; (**E**) for turquoise module; (**F**) for yellow module.

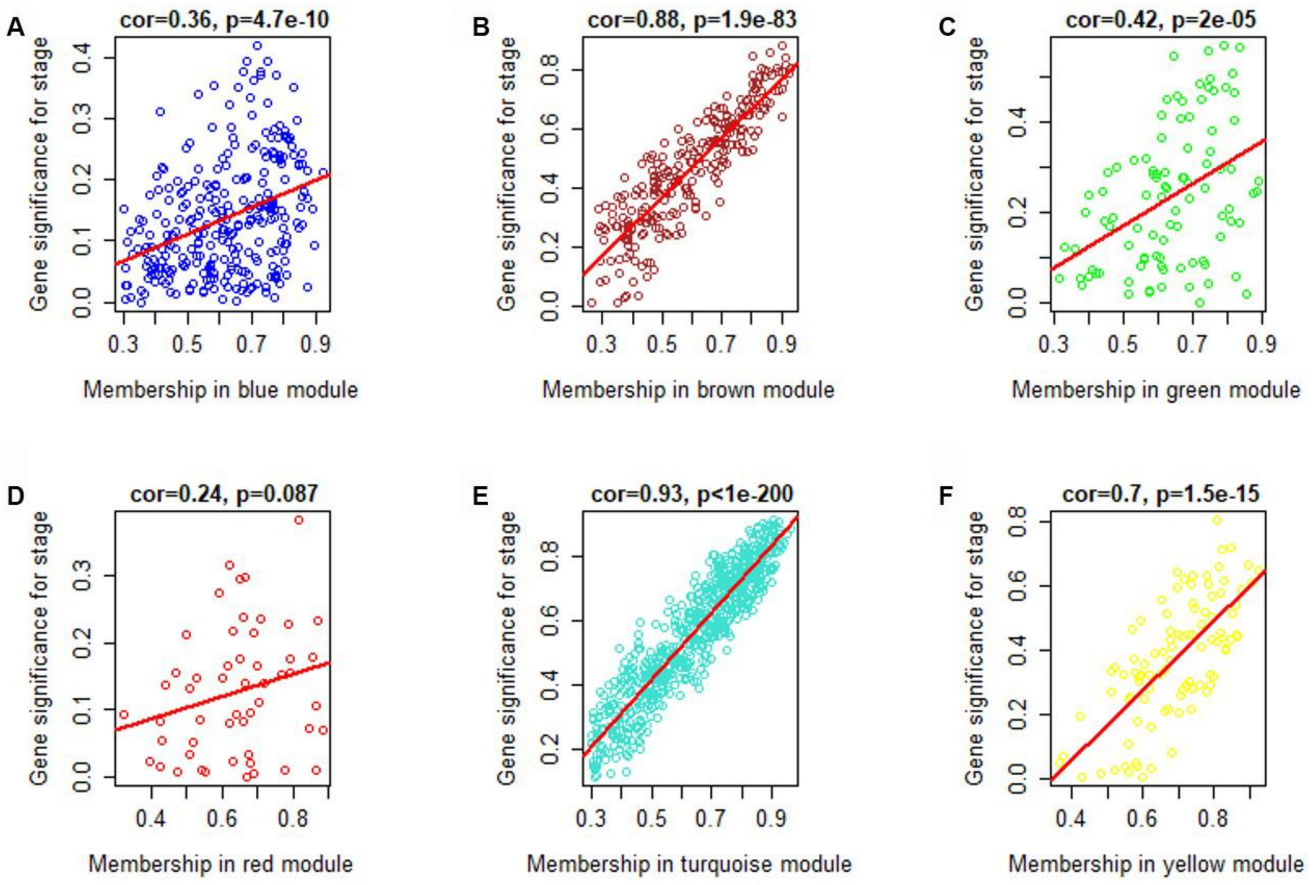

Supplementary Figure 9. Scatterplot of gene significance for developmental time points (stages; y axis) vs. module membership (x axis) in different modules. (**A**) For blue module; (**B**) for brown module; (**C**) for green module; (**D**) for red module; (**E**) for turquoise module; (**F**) for yellow module. For the most significant modules (i.e., blue, brown, green, turquoise, and yellow), genes with high module membership often also show high gene significance. Gene significance and module membership have a highly significant correlation, indicating that hub genes of these modules also predispose to high correlation with weight.

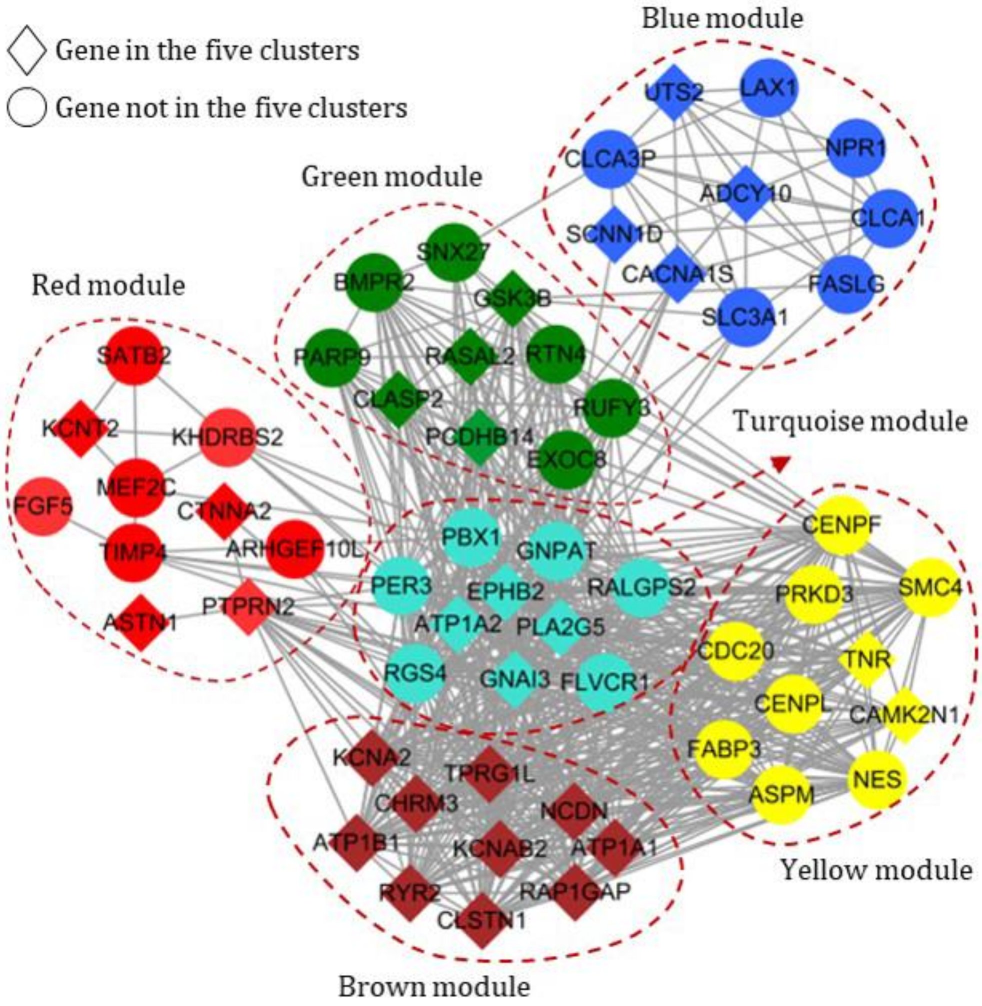

Supplementary Figure 10. Network plot of 10 hub genes from each module showing clustering across different brain regions and developmental time points. The nodes (genes) are colored by module, while the edges reflect positive correlations across brain regions and developmental epochs. The rectangular nodes represent genes clustered in the 18 shared pathways, and the circular nodes mark genes not so clustered.

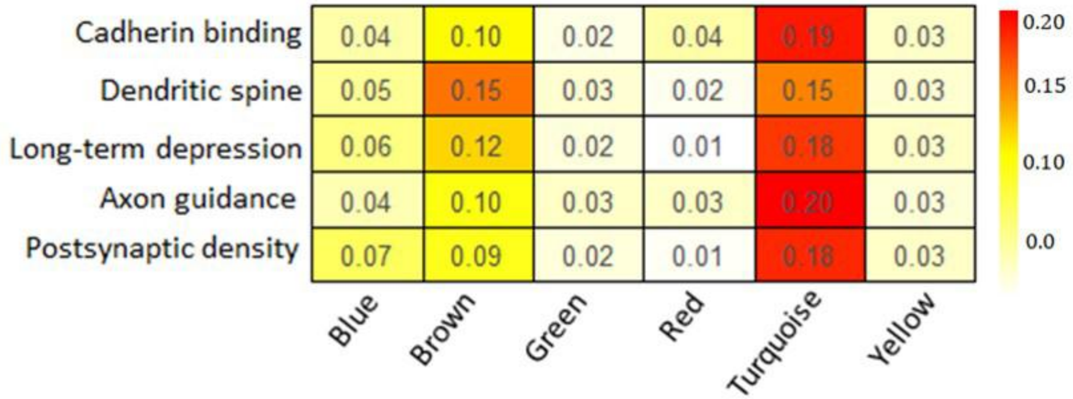

Supplementary Figure 11. Enrichment of the five clusters of 18 shared pathways in six co-expression modules (red = cluster with the highest proportion in a module). More than 15% of the genes in the five clusters among 18 shared pathways were in the turquoise module.

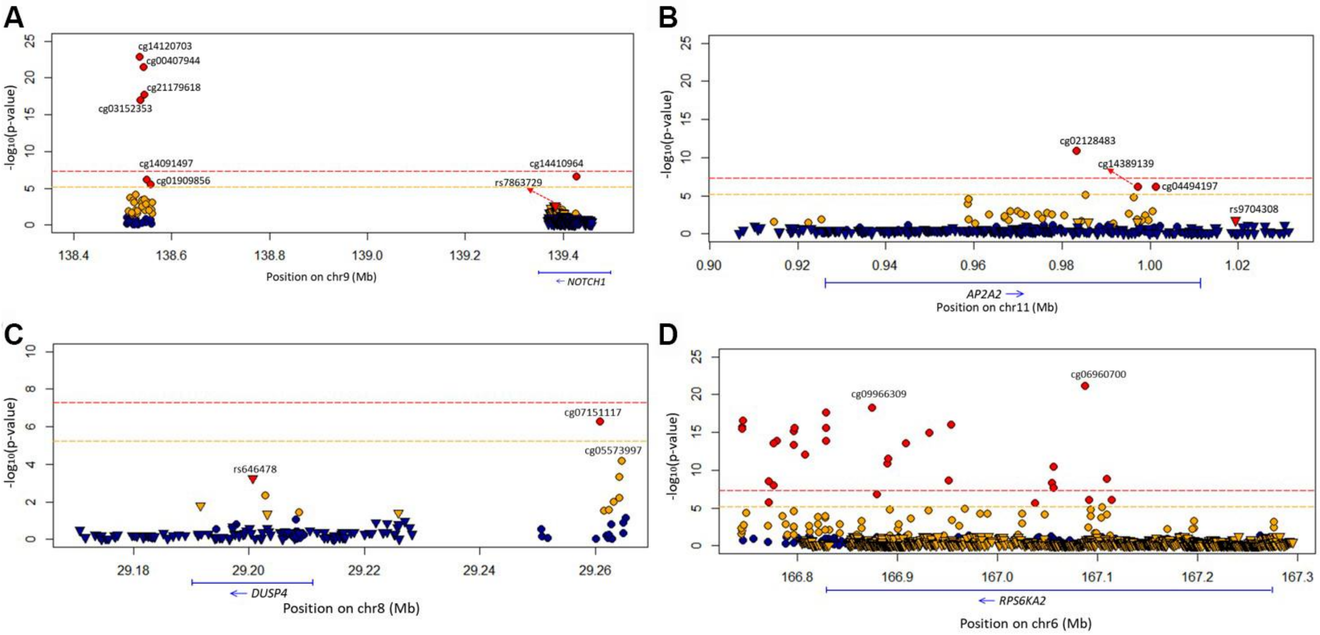

Supplementary Figure 12. Regional plot of association between genetic and epigenetic variation at the four genomic loci and SCZ. (**A**) For *NOTCH1*; (**B**) for *AP2A2*; (**C**) for *DUSP4*; (**D**) for *RPS6KA2*. Circular symbols indicate the association of CpG loci with SCZ (red represents these loci significantly associated with SCZ with $P \leq 6.07 \times 10^{-6}$; orange represents these loci with $6.07 \times 10^{-6} < P \leq 0.05$; blue represents these loci with $P > 0.05$). Triangular symbols indicate the association of SNPs with SCZ (red represents the top-ranked SNPs associated with SCZ; orange represents these SNPs associated with SCZ with $P \leq 0.05$; blue represents these SNPs with $P > 0.05$).

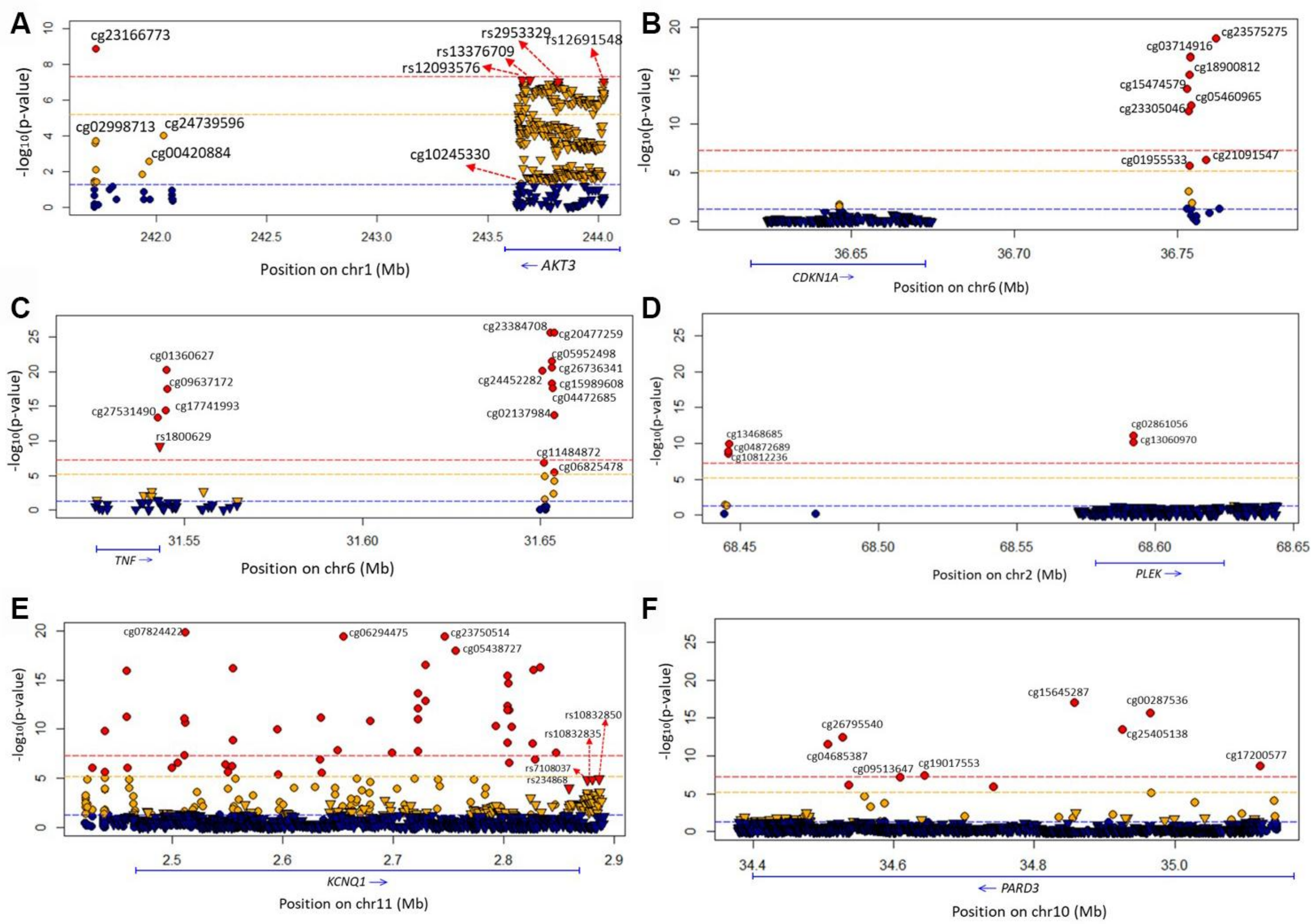

Supplementary Figure 13. Regional plot of association between genetic and epigenetic variation at the six genomic loci and SCZ. (**A**) For *AKT3*; (**B**) for *CDKN1A*; (**C**) for *TNF*; (**D**) for *PLEK*; (**E**) for *KCNQ1*; (**F**) for *PARD3*. Circular symbols indicate the association of CpG loci with SCZ (red represents these loci significantly associated with SCZ with $P \leq 6.07 \times 10^{-6}$; orange represents these loci with $6.07 \times 10^{-6} < P \leq 0.05$; blue represents these loci with $P > 0.05$). Triangular symbols indicate the association of SNPs with SCZ (red represents the top-ranked SNPs associated with SCZ; orange represents these SNPs associated with SCZ with $P \leq 0.05$; blue represents these SNPs with $P > 0.05$).

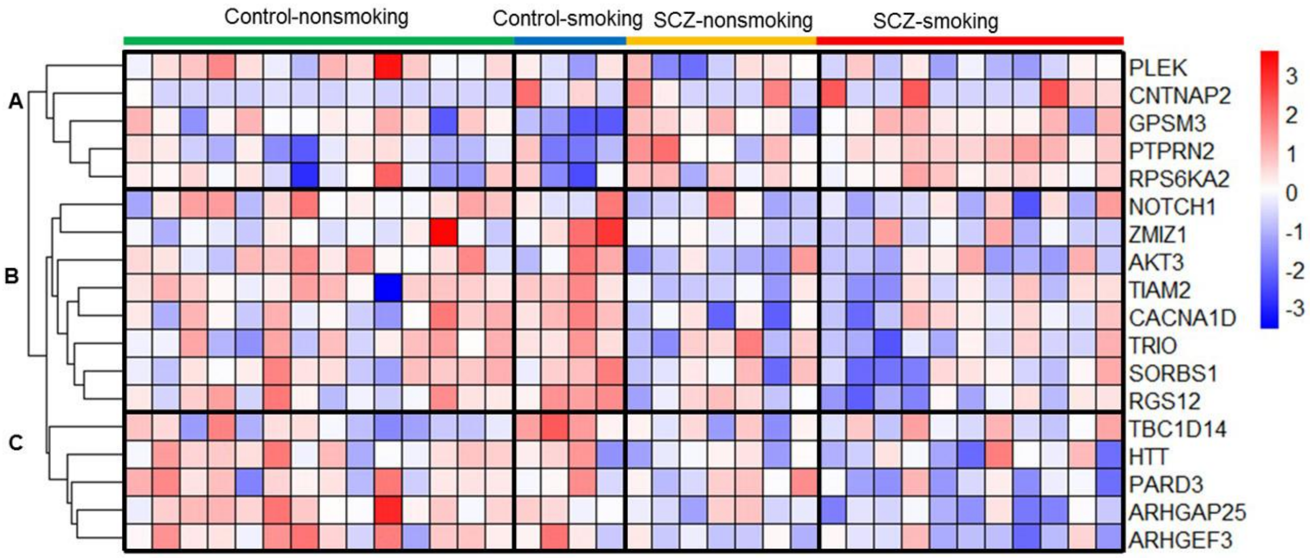

Supplementary Figure 14. Heatmap of significant genes from ANOVA by comparing the difference between SCZ patients and controls divided by smoking status. Colored rectangles represent transcript abundance indicated above the gene labeled on the right. The intensity of the color is proportional to the standardized values between -3 and 3 for each gene, as indicated on the bar on the right of the heat map plot. Clustering was performed using Euclidean distance according to the scale on the left. Major gene transcription patterns are arbitrarily described as A, B, and C. Heatmap plots were generated using the *pheatmap* package in R.

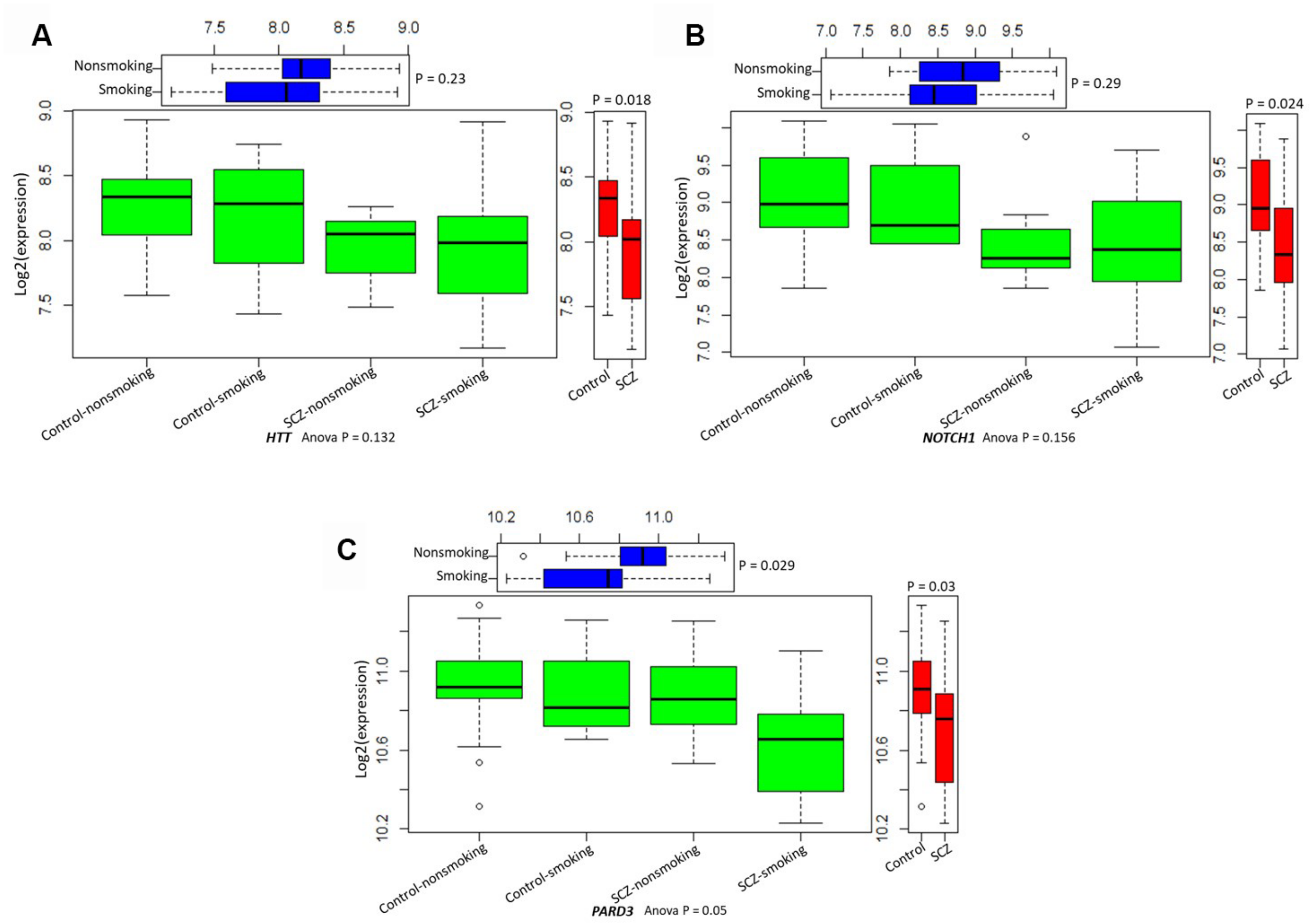

Supplementary Figure 15. Boxplots of 3 genes' expression patterns in SCZ patients and controls divided by smoking status. (**A**) For *HTT*; (**C**) for *NOTCH1*; (**D**) for *PARD3*.

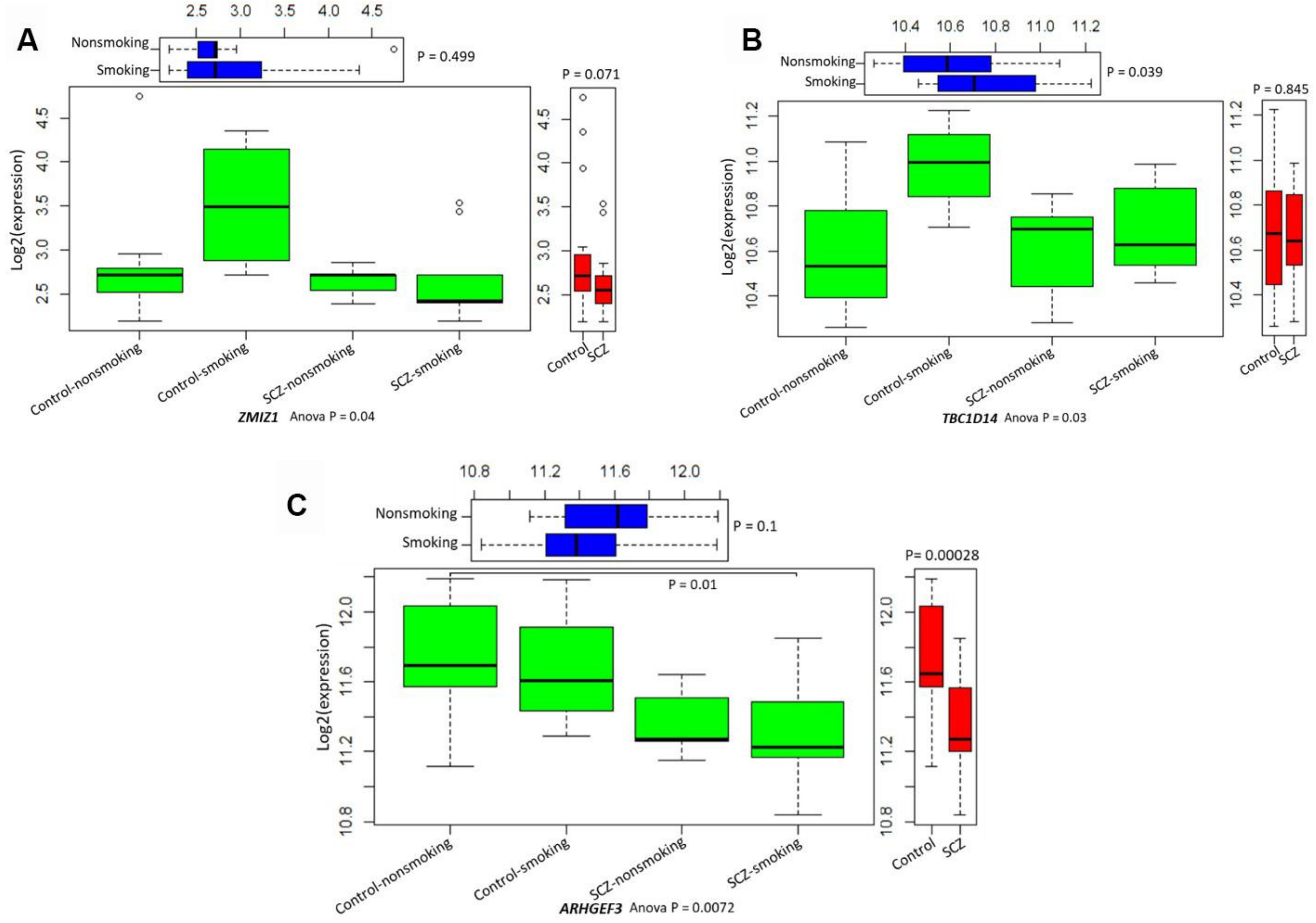

Supplementary Figure 16. Boxplots of 3 genes' expression patterns in SCZ patients and controls divided by smoking status. (**A**) For *ZMIZ1*; (**B**) for *TBC1D14*; (**C**) for *ARHGEF3*.

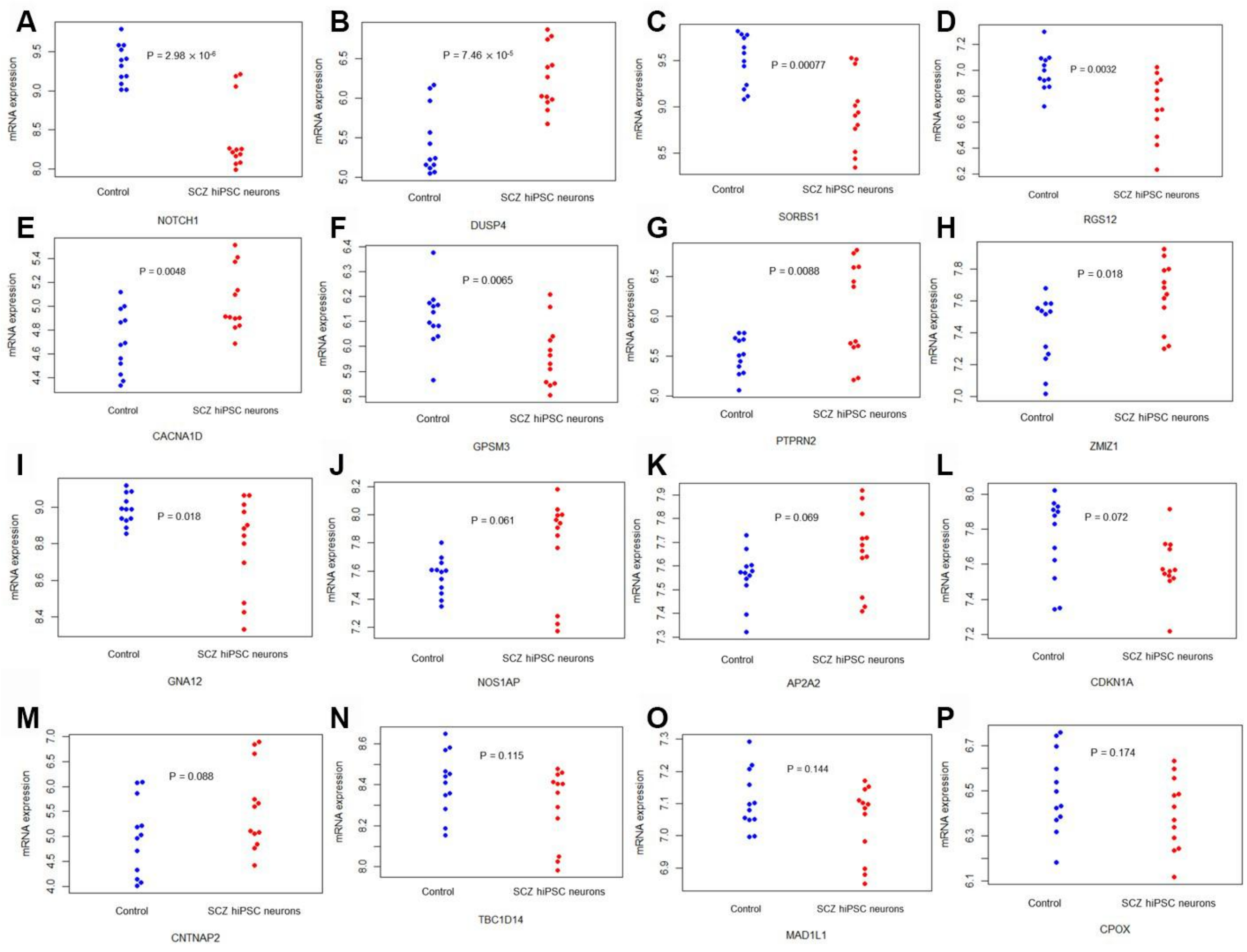

Supplementary Figure 17. Plots summarizing significant gene expression alterations in schizophrenic hiPSC-derived neurons. Student's *t*-test was used for comparing the difference between SCZ hiPSC neurons and controls. Gene expression data from the GEO dataset (Accession No. GSE25673). (**A**) For *NOTCH1*; (**B**) for *DUSP4*; (**C**) for *SORBS1*; (**D**) for *RGS12*; (**E**) for *CACNA1D*; (**F**) for *GPSM3*; (**G**) for *PTPRN2*; (**H**) for *ZMIZ1*; (**I**) for *GNA12*; (**J**) for *NOS1AP*; (**K**) for *AP2A2*; (**L**) for *CDKN1A*; (**M**) for *CNTNAP2*; (**N**) for *TBC1D14*; (**O**) for *MAD1L1*; (**P**) for *CPOX*. These plots were generated using the *beeswarm* package in R.

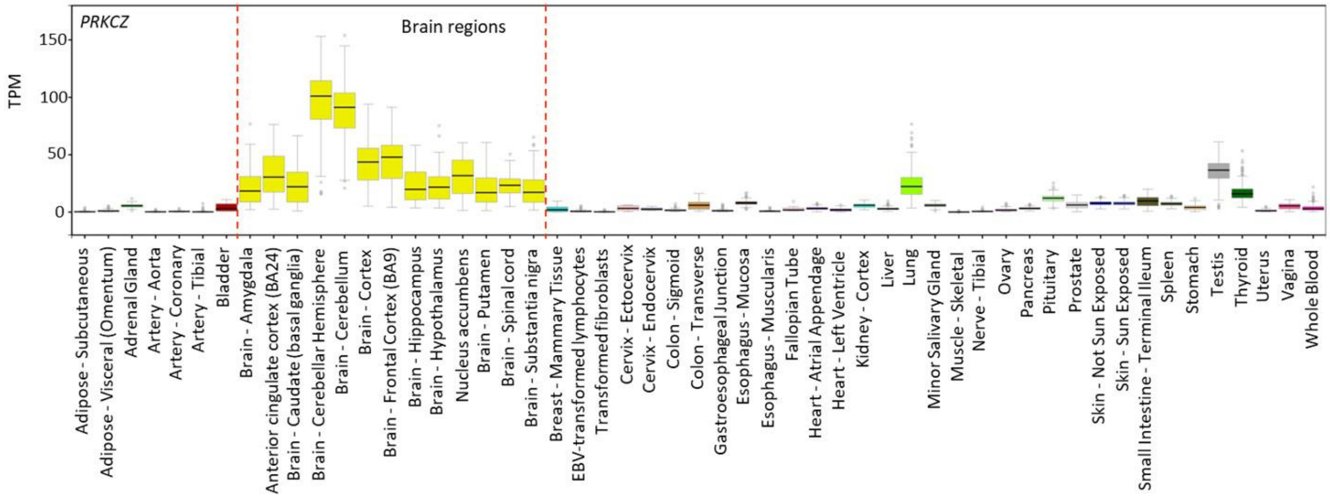

Supplementary Figure 18. An example of *PRKCZ* expression in 53 human tissues, which were obtained from GTEx analysis release V7 (dbGaP Accession No. phs000424.v7.p2). Expression values are shown in transcripts per million (TPM), calculated from a gene model with isoforms collapsed into a single gene. Box plots describe median and 25th and 75th percentiles; points are displayed as outliers if they are above or below 1.5 times the interquartile range.

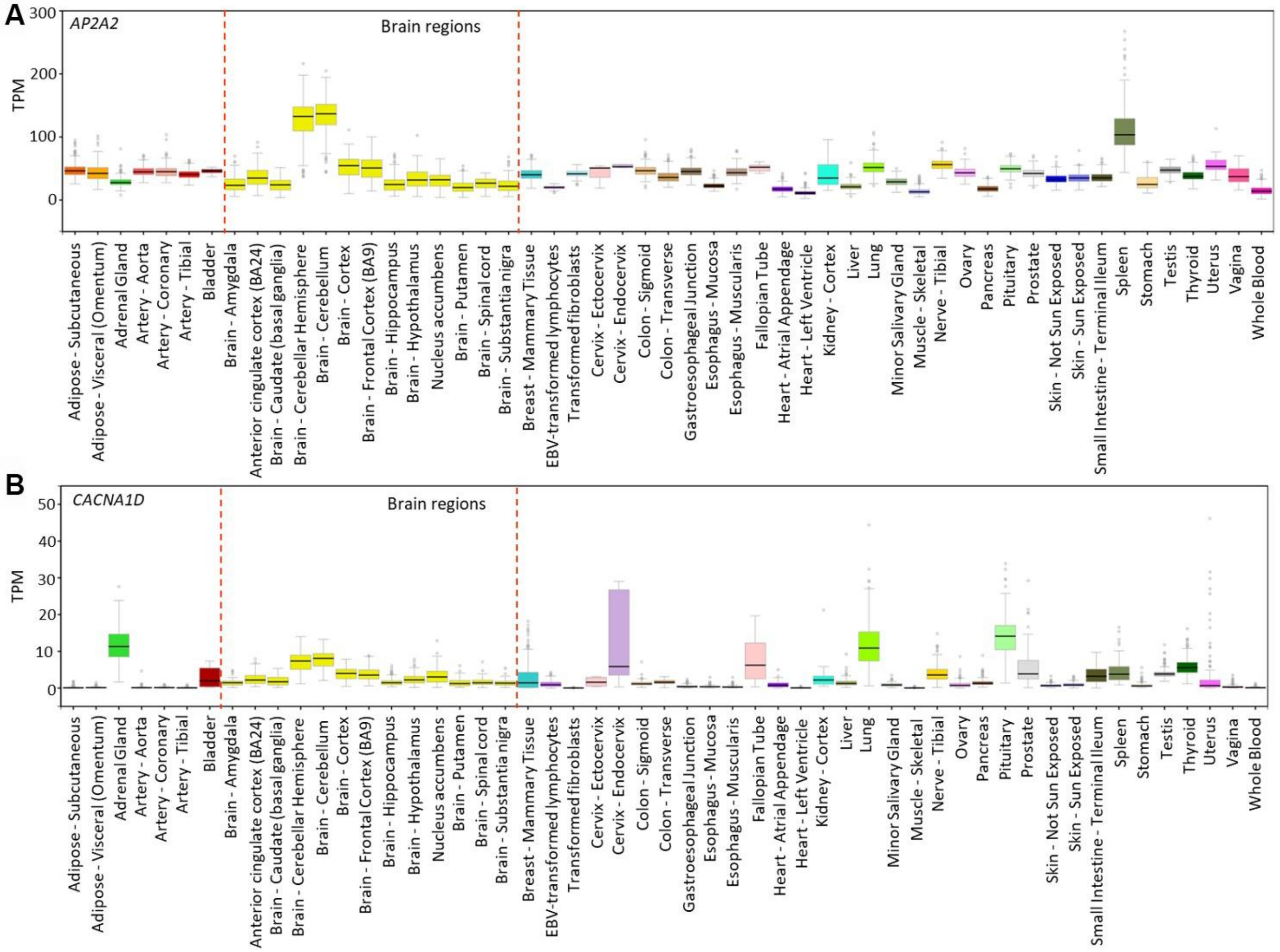

Supplementary Figure 19. Expression of (**A**) *AP2A2* and (**B**) *CACNA1D* in 53 human tissues. The expression data were obtained from GTEx analysis release V7 (dbGaP Accession No. phs000424.v7.p2). Expression values are shown in transcripts per million (TPM), calculated from a gene model with isoforms collapsed to a single gene. Box plots are shown as median and 25th and 75th percentiles; points are displayed as outliers if they are above or below 1.5 times the interquartile range.

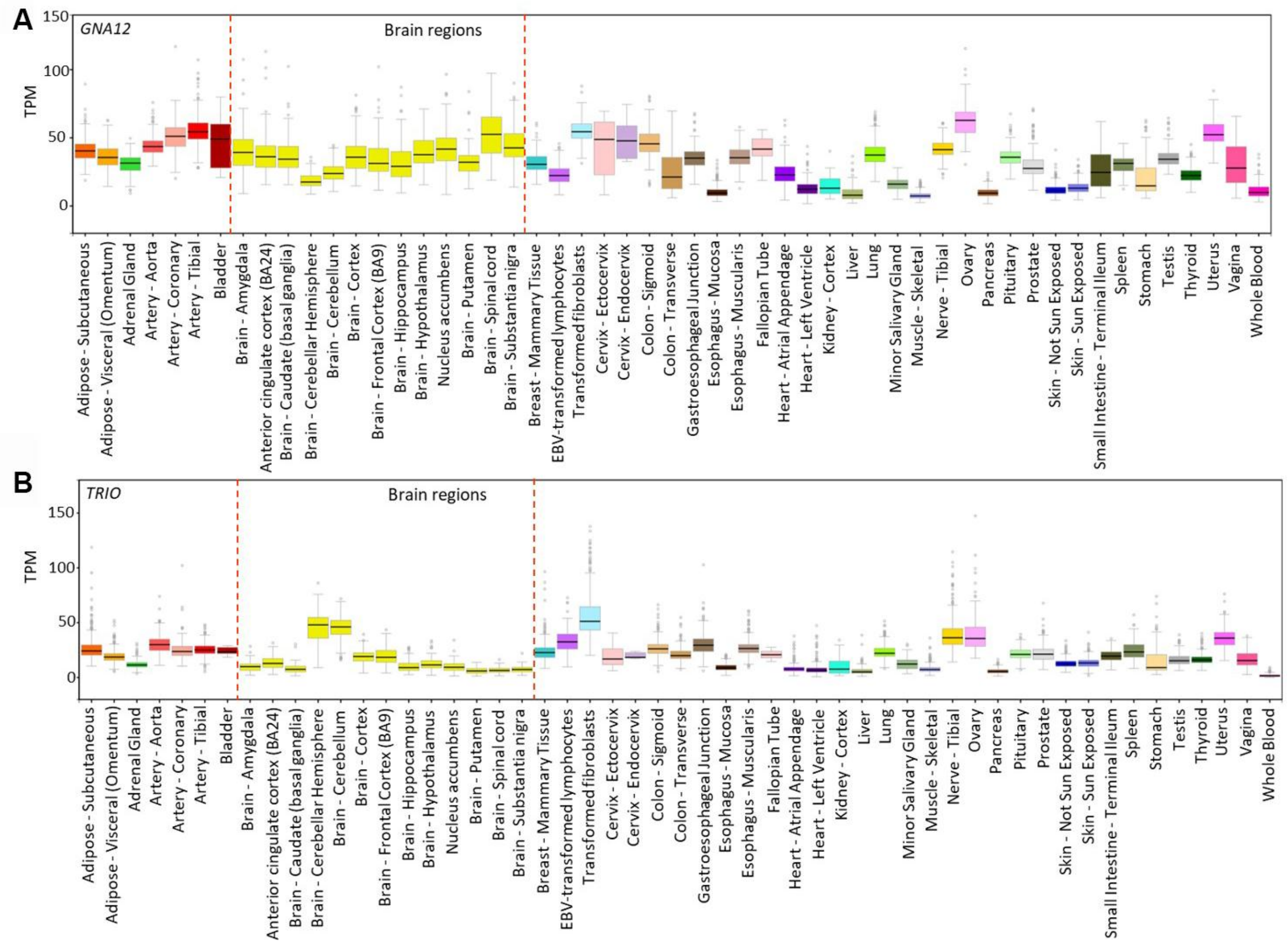

Supplementary Figure 20. Expression of (**A**) *GNA12* and (**B**) *TRIO* in 53 human tissues. The expression data were obtained from GTEx analysis release V7 (dbGaP Accession No. phs000424.v7.p2). Expression values are shown in transcripts per million (TPM), calculated from a gene model with isoforms collapsed to a single gene. Box plots are shown as median and 25th and 75th percentiles; points are displayed as outliers if they are above or below 1.5 times the interquartile range.

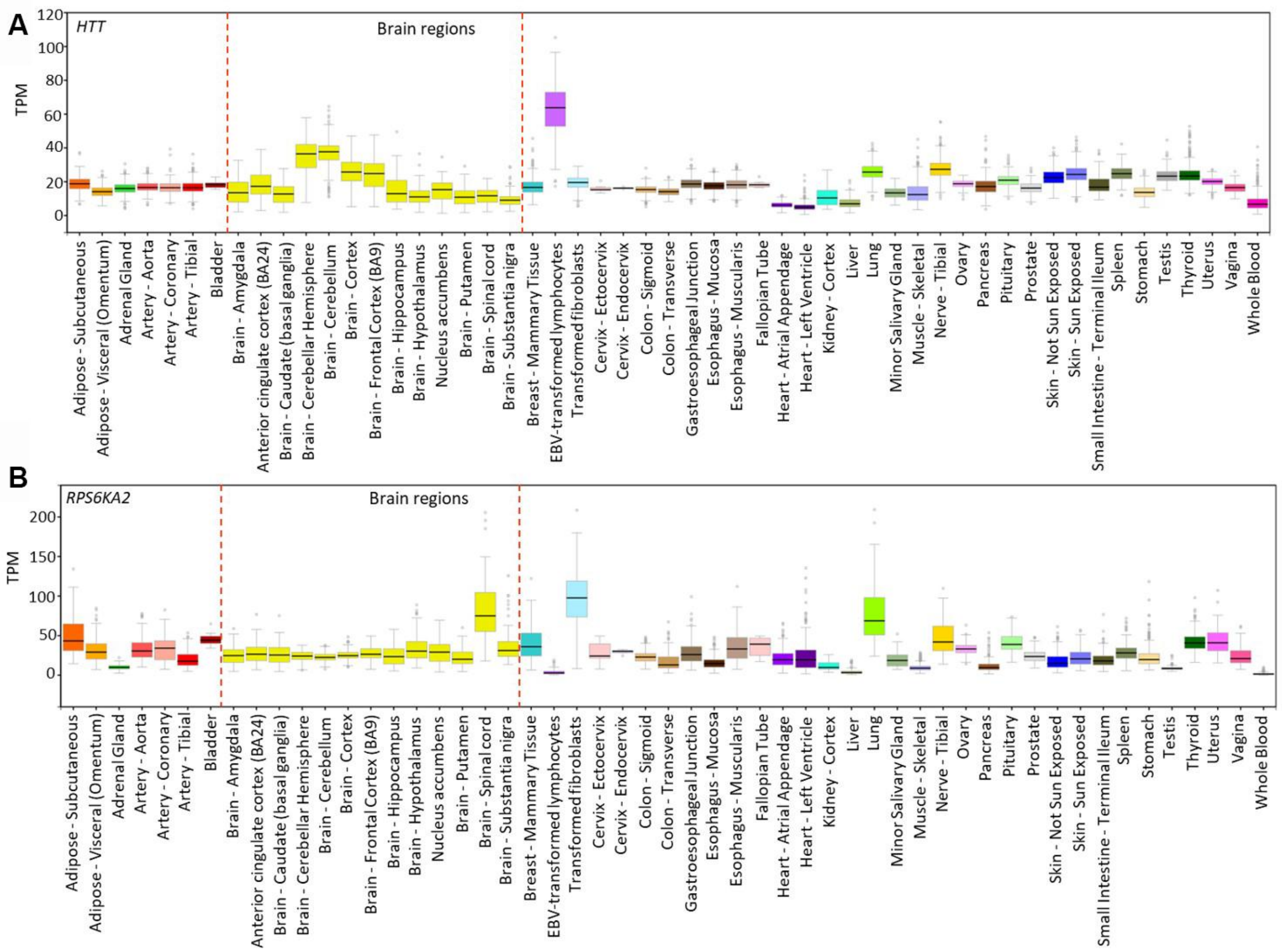

Supplementary Figure 21. Expression of (**A**) *HTT* and (**B**) *RPS6KA2* in 53 human tissues. The expression data were obtained from GTEx analysis release V7 (dbGaP Accession No. phs000424.v7.p2). Expression values are shown in transcripts per million (TPM), calculated from a gene model with isoforms collapsed to a single gene. Box plots are shown as median and 25th and 75th percentiles; points are displayed as outliers if they are above or below 1.5 times the interquartile range.

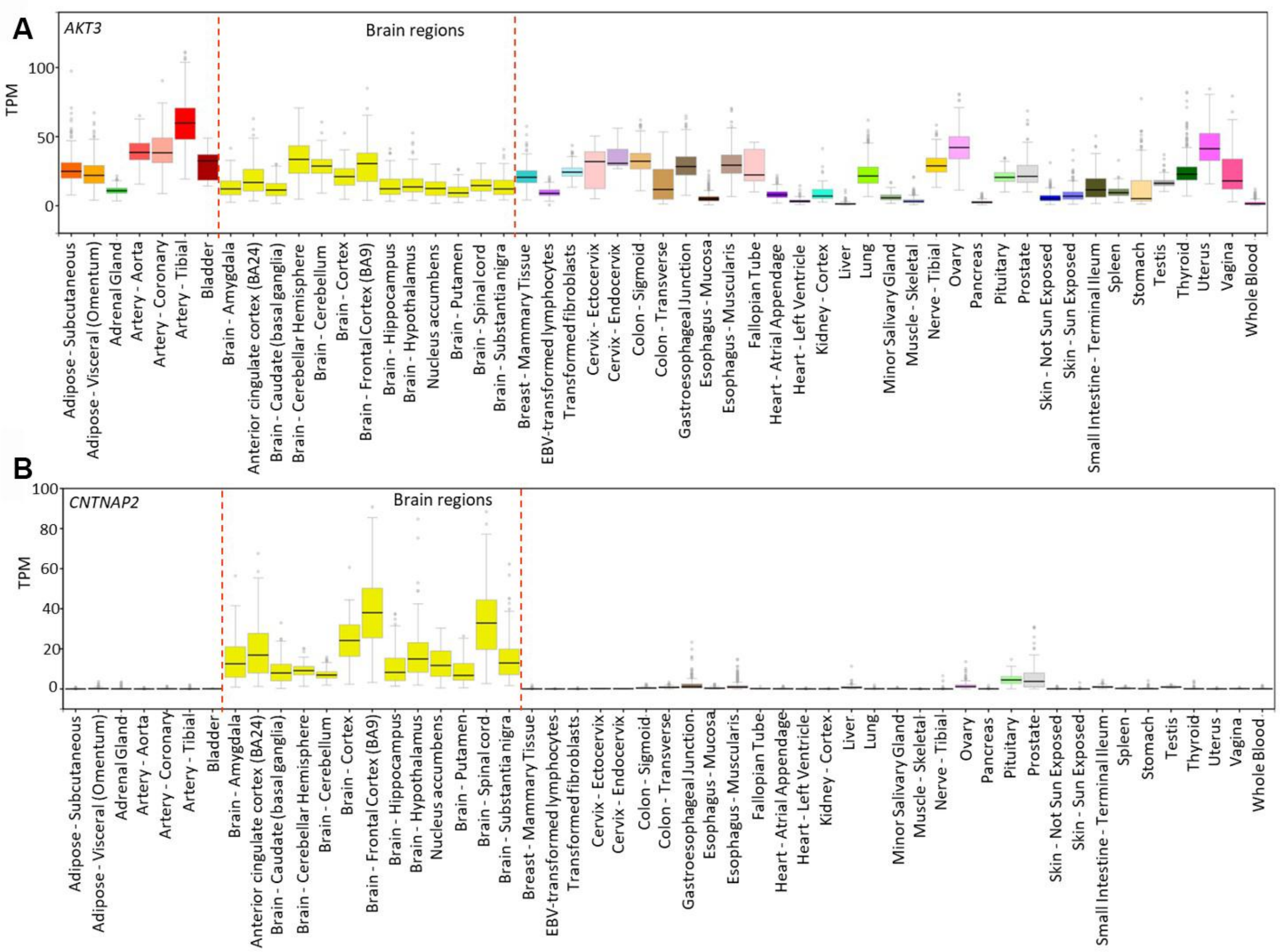

Supplementary Figure 22. Expression of (**A**) *AKT3* and (**B**) *CNTNAP2* in 53 human tissues. The expression data were obtained from GTEx analysis release V7 (dbGaP Accession No. phs000424.v7.p2). Expression values are shown in transcripts per million (TPM), calculated from a gene model with isoforms collapsed to a single gene. Box plots are shown as median and 25th and 75th percentiles; points are displayed as outliers if they are above or below 1.5 times the interquartile range.

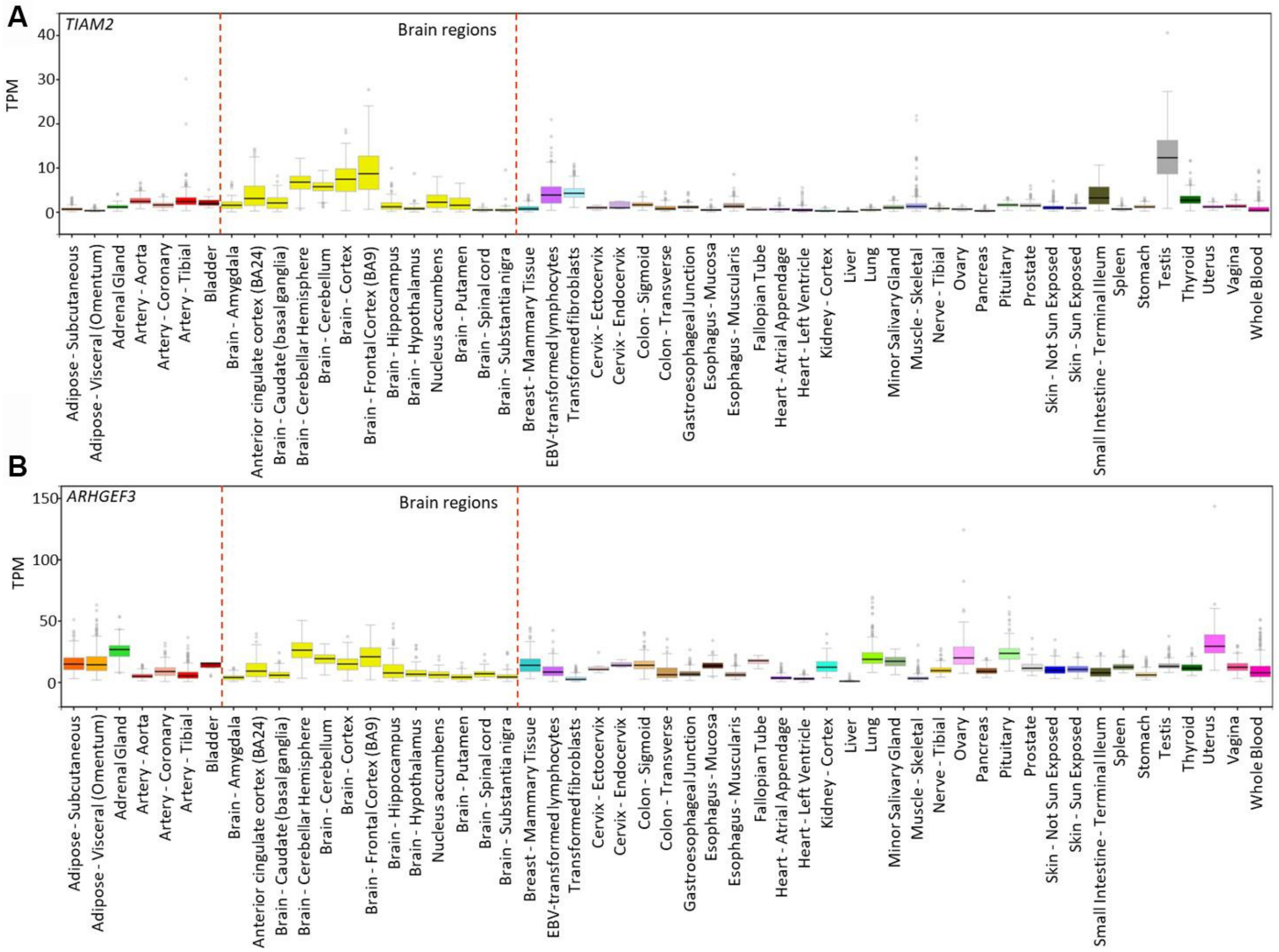

Supplementary Figure 23. Expression of (**A**) *TIAM2* and (**B**) *ARHGEF3* in 53 human tissues. The expression data were obtained from GTEx analysis release V7 (dbGaP Accession phs000424.v7.p2). Expression values are shown in transcripts per million (TPM), calculated from a gene model with isoforms collapsed to a single gene. Box plots are shown as median and 25th and 75th percentiles; points are displayed as outliers if they are above or below 1.5 times the interquartile range.

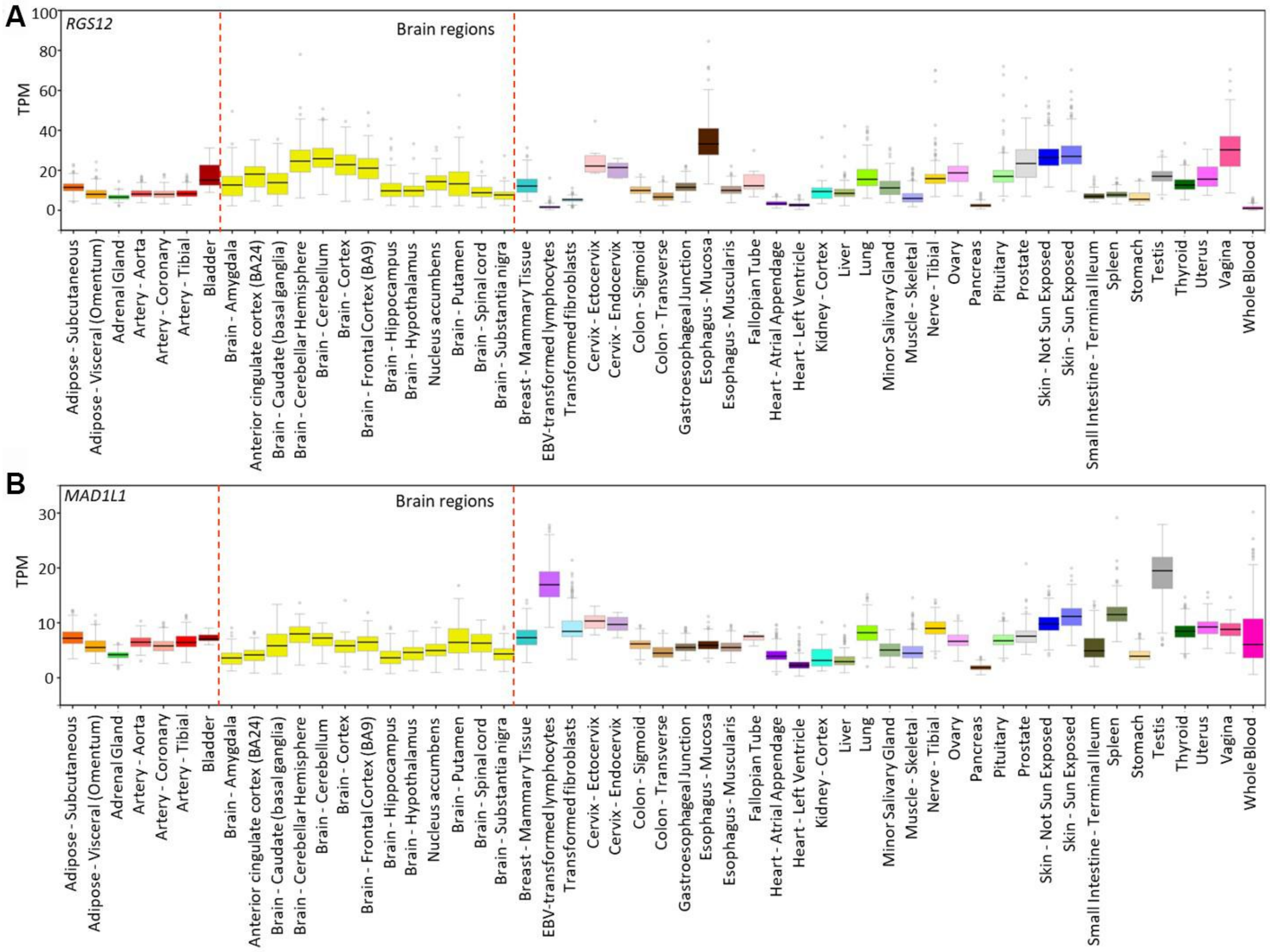

Supplementary Figure 24. Expression of (**A**) *RGS12* and (**B**) *MAD1L1* in 53 human tissues. The expression data were obtained from GTEx analysis release V7 (dbGaP Accession No. phs000424.v7.p2). Expression values are shown in transcripts per million (TPM), calculated from a gene model with isoforms collapsed to a single gene. Box plots are shown as median and 25th and 75th percentiles; points are displayed as outliers if they are above or below 1.5 times the interquartile range.

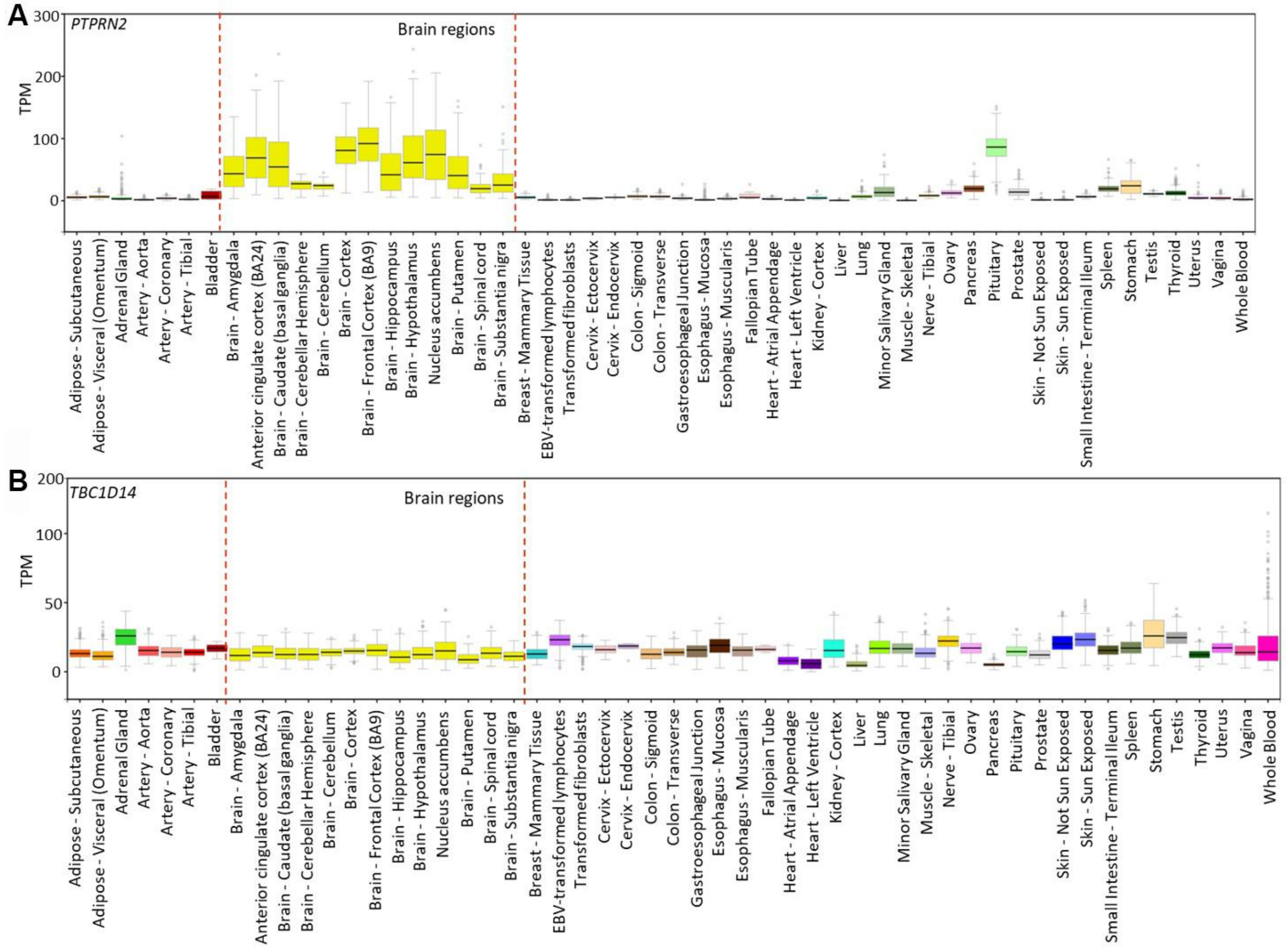

Supplementary Figure 25. Expression of (**A**) *PTPRN2* and (**B**) *TBC1D14* in 53 human tissues. The expression data were obtained from GTEx analysis release V7 (dbGaP Accession No. phs000424.v7.p2). Expression values are shown in transcripts per million (TPM), calculated from a gene model with isoforms collapsed to a single gene. Box plots are shown as median and 25th and 75th percentiles; points are displayed as outliers if they are above or below 1.5 times the interquartile range.

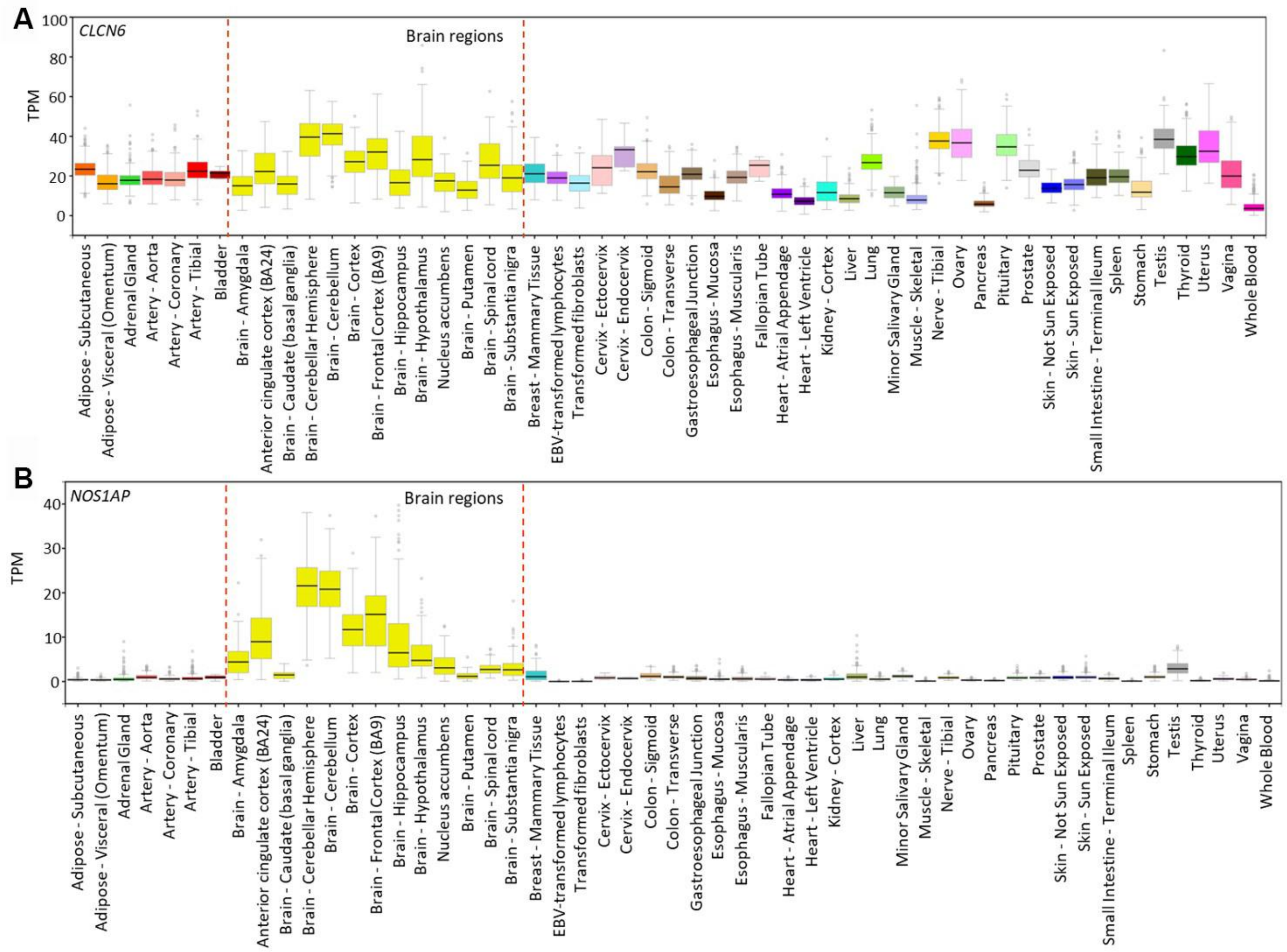

Supplementary Figure 26. Expression of (**A**) *CLCN6* and (**B**) *PRKCZ* in 53 human tissues. The expression data were obtained from GTEx analysis release V7 (dbGaP Accession phs000424.v7.p2). Expression values are shown in transcripts per million (TPM), calculated from a gene model with isoforms collapsed to a single gene. Box plots are shown as median and 25th and 75th percentiles; points are displayed as outliers if they are above or below 1.5 times the interquartile range.

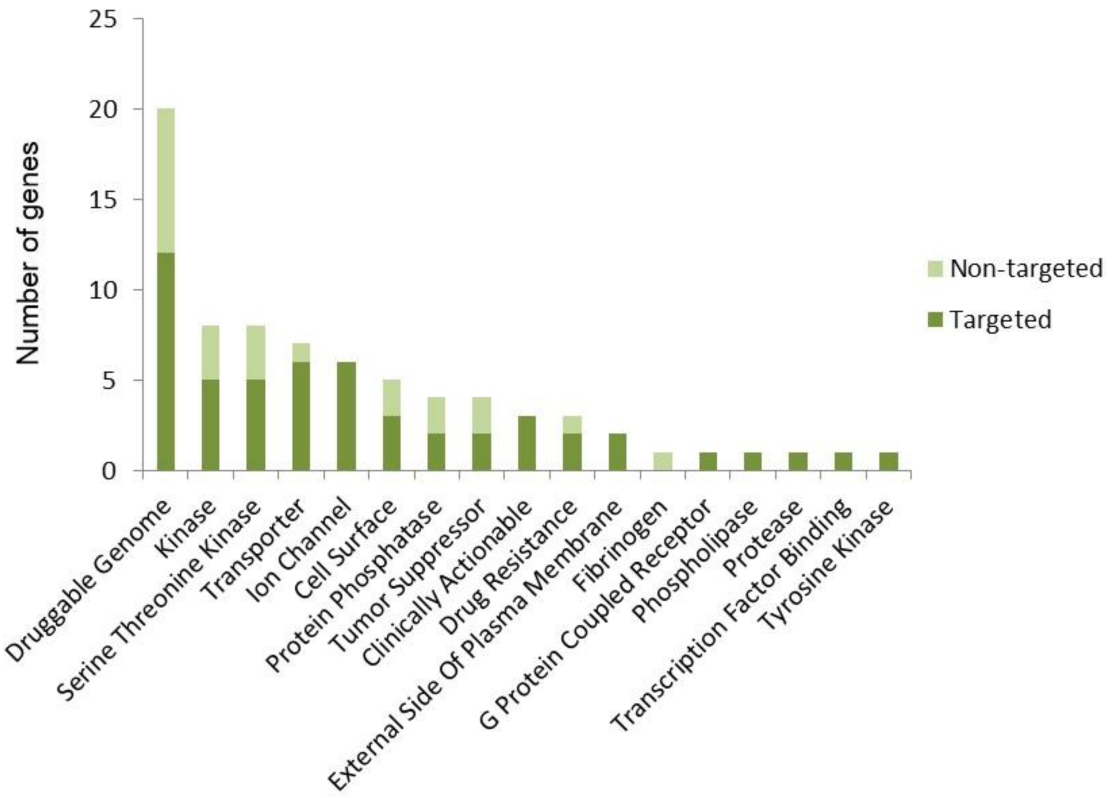

Supplementary Figure 27. Summary of candidate genes in potentially druggable categories. The numbers of these genes in potentially druggable categories, and the numbers of genes in these categories that are targeted by a known drug.

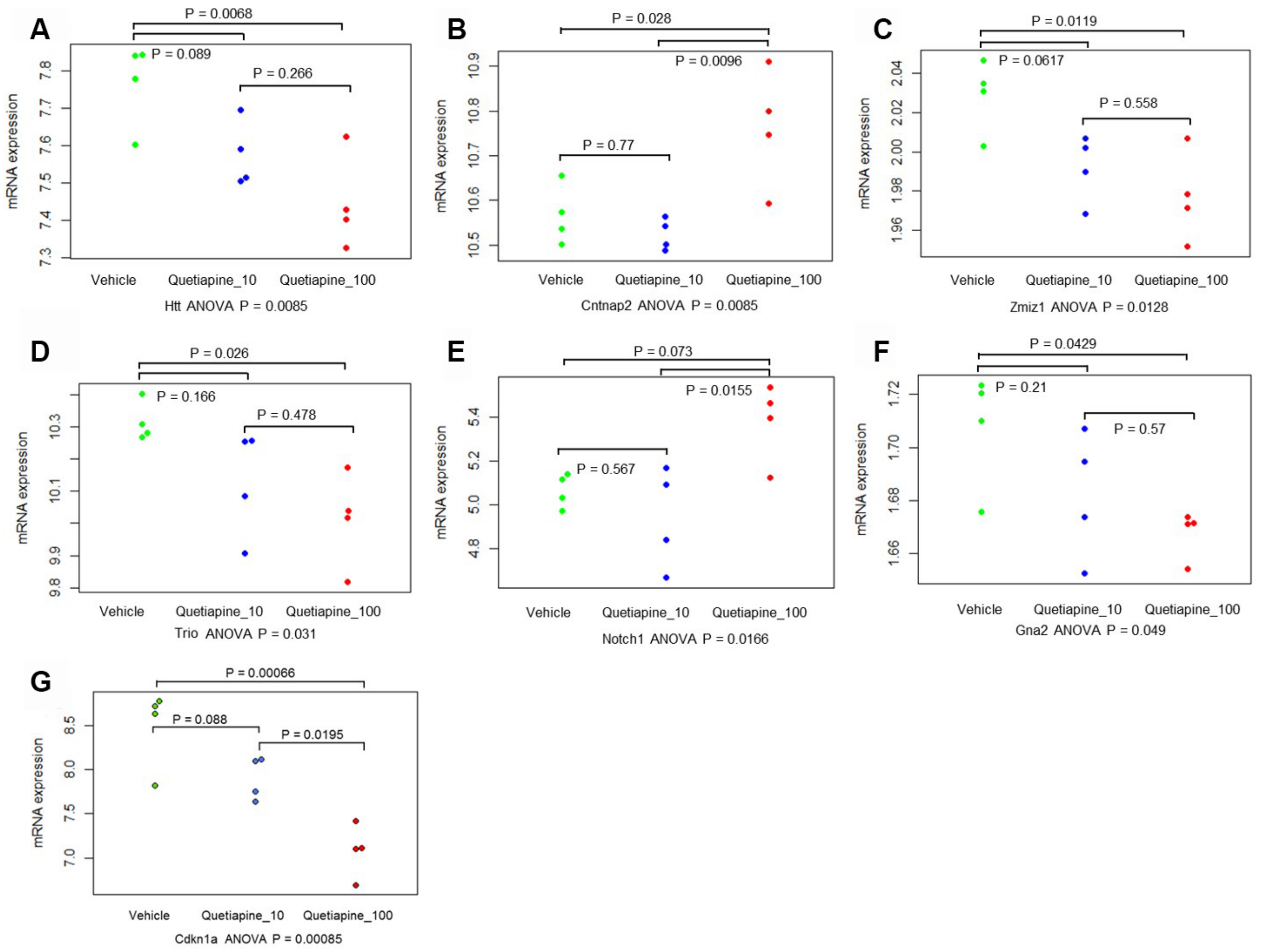

Supplementary Figure 28. Plots summarizing significant gene expression alterations in quetiapine (at doses of 10 or 100 mg/kg)-treated mice. One-way ANOVA was applied, and Tukey-Kramer HSD test was used for multiple comparisons. Gene expression data from the GEO dataset (Accession No. GSE45229). (**A**) For *Htt*; (**B**) for *Cntnap2*; (**C**) for *Zmiz1*; (**D**) for *Trio*; (**E**) for *Notch1*; (**F**) for *Gna2*. These plots were generated using the *beeswarm* package in R.

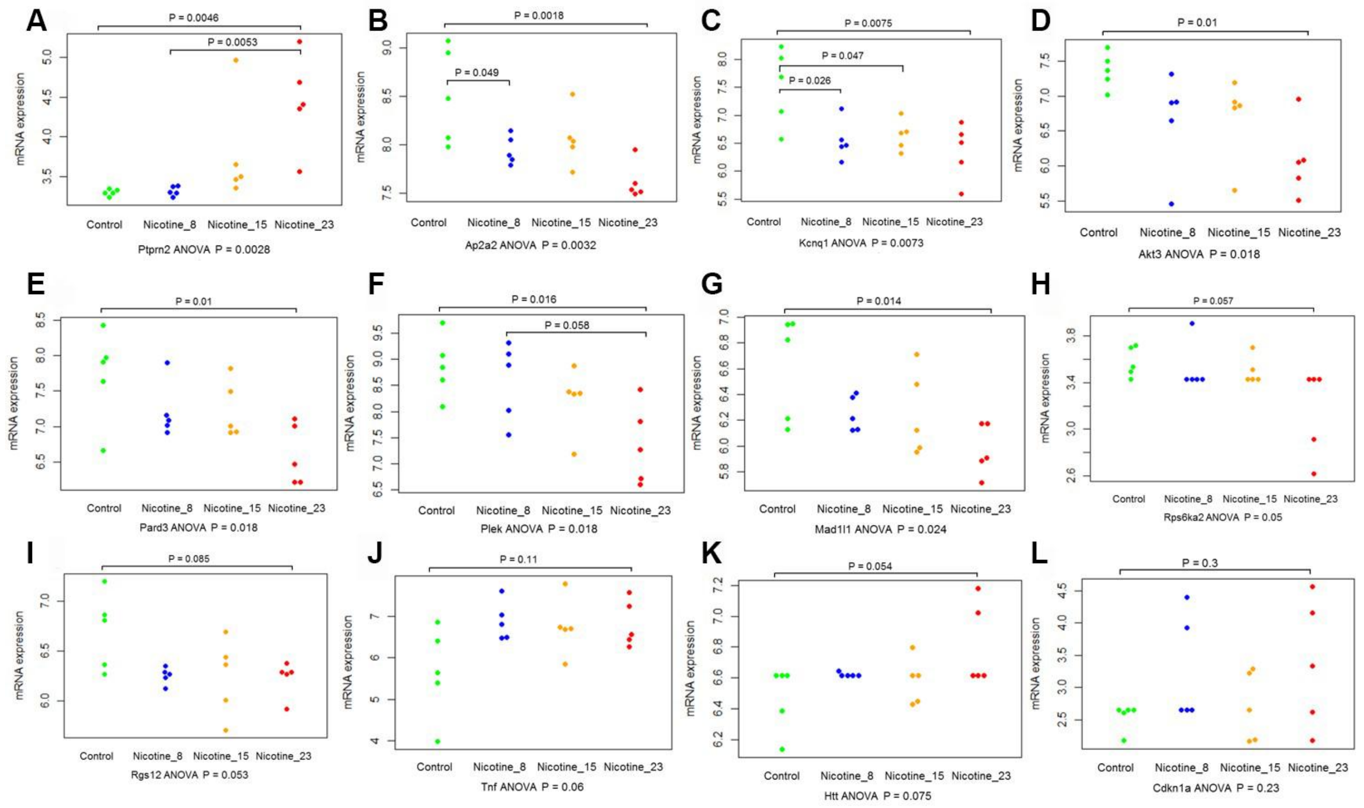

Supplementary Figure 29. Plots summarizing significant gene expression alterations in nicotine (at doses of 8 µg nicotine/l, 15 µg nicotine/l, and 23 µg nicotine/l)-treated mice. One-way ANOVA was applied, and Tukey-Kramer HSD test was used for multiple comparisons. Gene expression data from the GEO dataset (Accession No. GES50254). (**A**) For *Ptprn2*; (**B**) for *Ap2a2*; (**C**) for *Kcnq1*; (**D**) for *Akt3*; (**E**) for *Pard3*; (**F**) for *Plek*; (**G**) for *Mad1l1*; (**H**) for *Rps6ka2*; (**I**) for *Rgs12*; (**J**) for *Tnf*; (**K**) for *Htt*; (**L**) for *Cdkn1a*. These plots were generated using the *beeswarm* package in R.

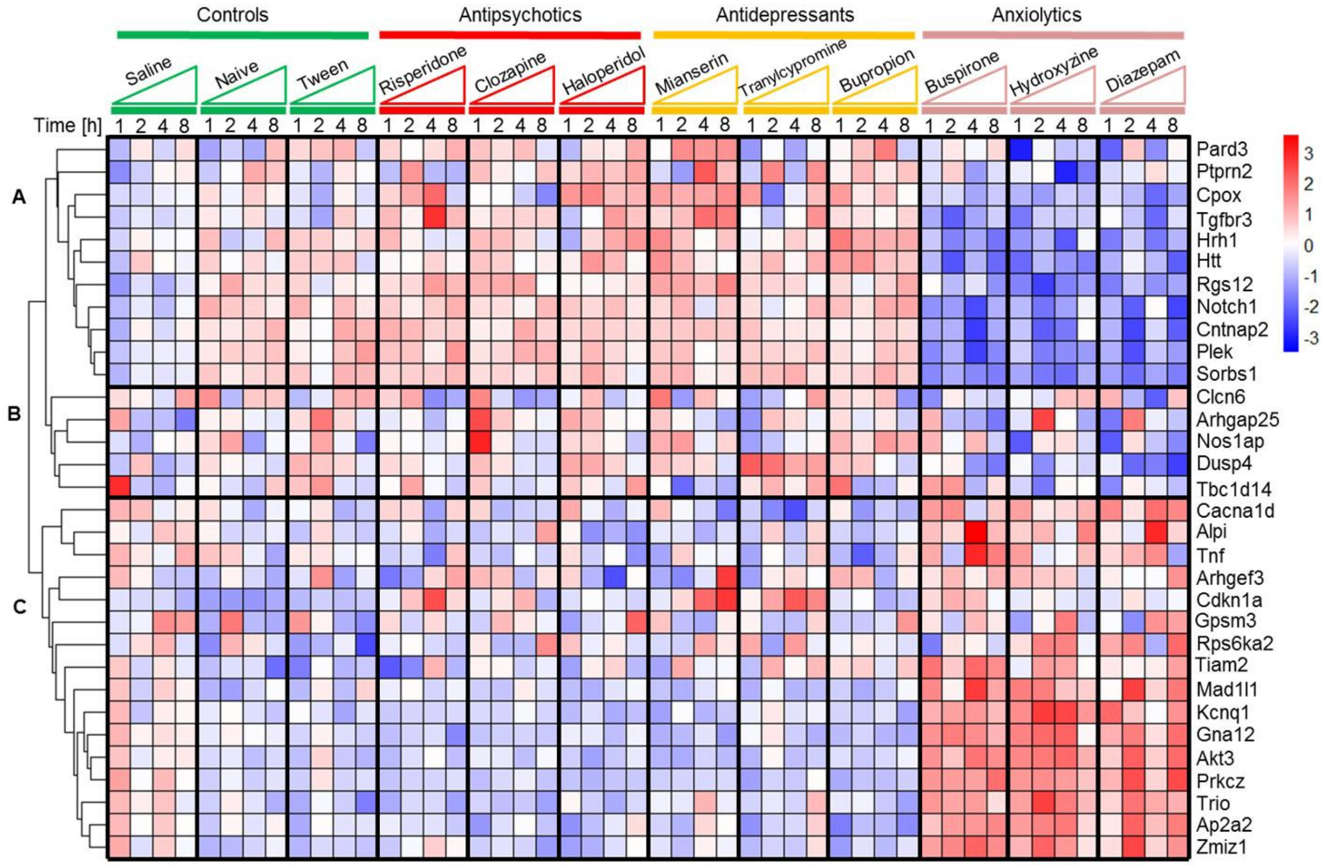

Supplementary Figure 30. Heatmap of the identified candidate genes treated by psychotropic drugs. Colored rectangles represent transcript abundance indicated above the gene labeled on the right. The intensity of the color is proportional to the standardized values between -3 and 3 for each gene, as indicated on the bar on the right of the heat map plot. Clustering was performed using Euclidean distance according to the scale on the left. Major gene transcription patterns are arbitrarily described as (**A**–**C**) Gene expression data from the GEO dataset (Accession No. GES50254). Three antidepressants (bupropion 20 mg/kg, tranylcypromine 20 mg/kg, mianserin 20 mg/kg, i.p.), three anxiolytics (diazepam 5 mg/kg, buspirone 10 mg/kg, hydroxyzine 10 mg/kg, i.p.), and three antipsychotics (clozapine 3 mg/kg, risperidone 0.5 mg/kg, haloperidol 1 mg/kg) were selected for the comparison. To analyze dynamics of early, intermediate, and relatively late changes of mRNA abundance, the experiment was performed at four time points (1, 2, 4, and 8h after drug administration). To exclude influence of drug injection and circadian rhythm on gene expression profile, control groups of saline or Tween (1% Tween 80)-treated and naïve animals were prepared for each time point. Heatmap plots were generated using the *pheatmap* package in R.

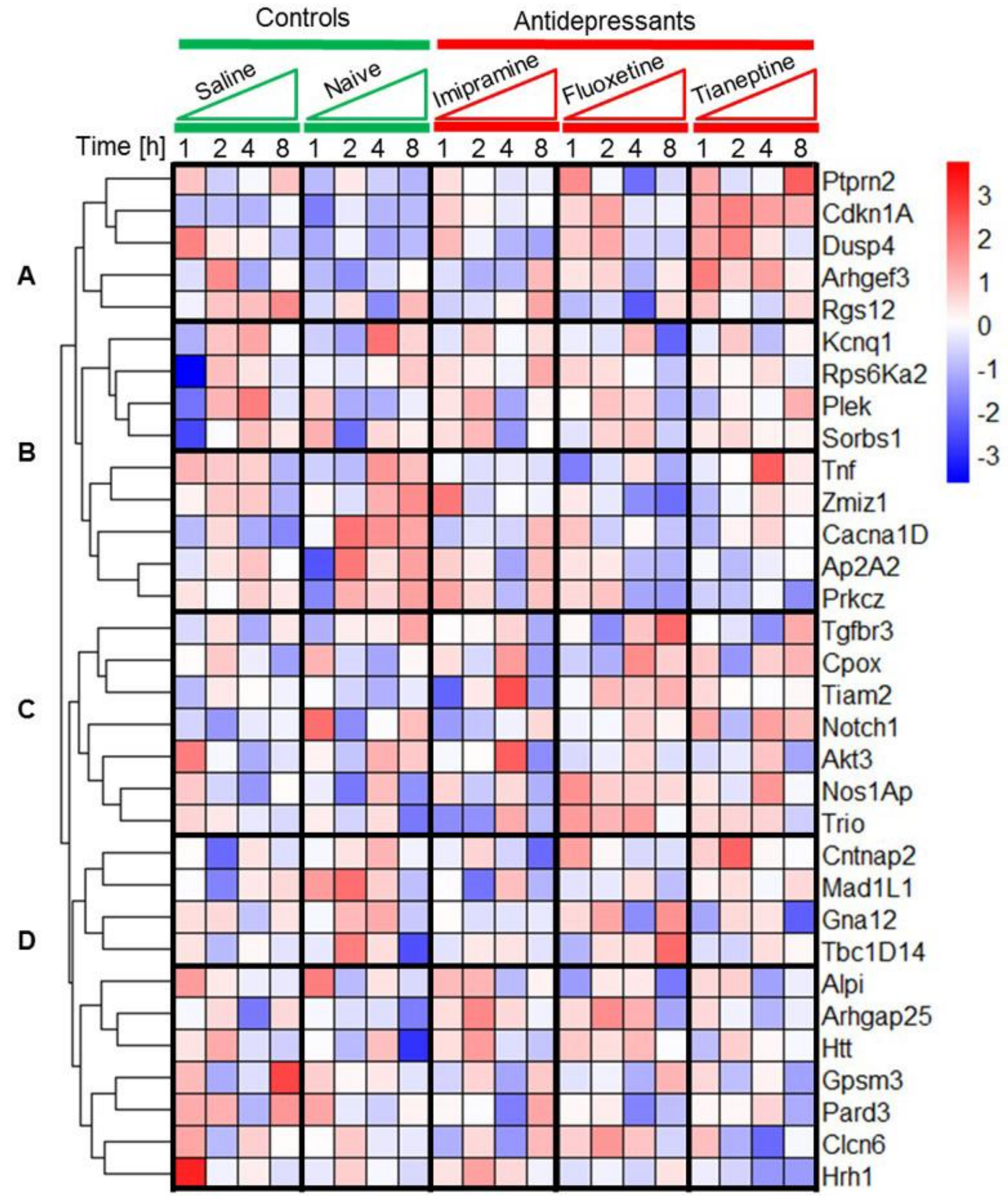

Supplementary Figure 31. Heatmap of the identified candidate genes treated by psychotropic drugs. Colored rectangles represent transcript abundance indicated above the gene labeled on the right. The intensity of the color is proportional to the standardized values between -3 and 3 for each gene, as indicated on the bar on the right of the heat map plot. Clustering was performed using Euclidean distance according to the scale on the left. Major gene transcription patterns are arbitrarily described as (**A**–**D**) Gene expression data from the GEO dataset (Accession No. GSE48951). Three antidepressants (imipramine 10 mg/kg, fluoxetine 20 mg/kg, and tianeptine 20 mg/kg, i.p.) were selected for the comparison. To analyze dynamics of early, intermediate, and relatively late changes of mRNA abundance, the experiment was performed at four time points (1, 2, 4, and 8h after drug administration). To exclude influence of drug injection and circadian rhythm on gene expression profile, control groups of saline or Tween (1% Tween 80)-treated and naïve animals were prepared for each time point. Heatmap plots were generated using the *pheatmap* package in R.

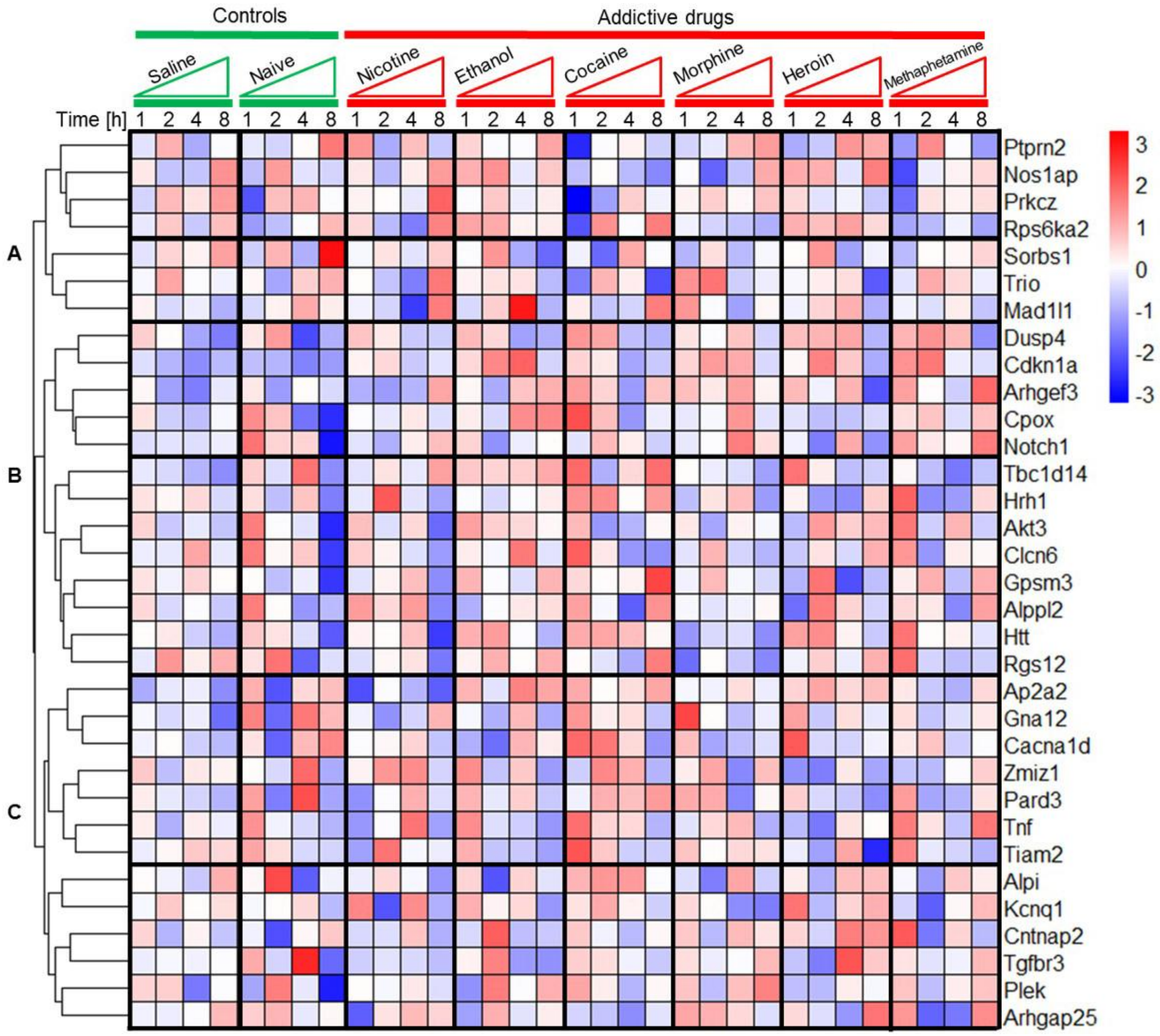

Supplementary Figure 32. Heatmap of the identified candidate genes treated by psychotropic drugs. Colored rectangles represent transcript abundance indicated above the gene labeled on the right. The intensity of the color is proportional to the standardized values between -3 and 3 for each gene, as indicated on the bar on the right of the heat map plot. Clustering was performed using Euclidean distance according to the scale on the left. Major gene transcription patterns are arbitrarily described as (**A**–**C**) Gene expression data from the GEO dataset (Accession No. GSE15774). Six of the most addictive and harmful drugs of abuse (morphine 20 mg/kg, heroin 10 mg/kg, ethanol 2 g/kg, nicotine 1 mg/kg, methamphetamine 2 mg/kg, or cocaine 25 mg/kg, i.p.) were selected for comparison. To analyze dynamics of early, intermediate, and relatively late changes of mRNA abundance, the experiment was performed at four time points (1, 2, 4, and 8h after drug administration). To exclude influence of drug injection and circadian rhythm on gene expression profile, control groups of saline-treated and naïve animals were prepared for each time point. Heatmap plots were generated using the *pheatmap* package in R.

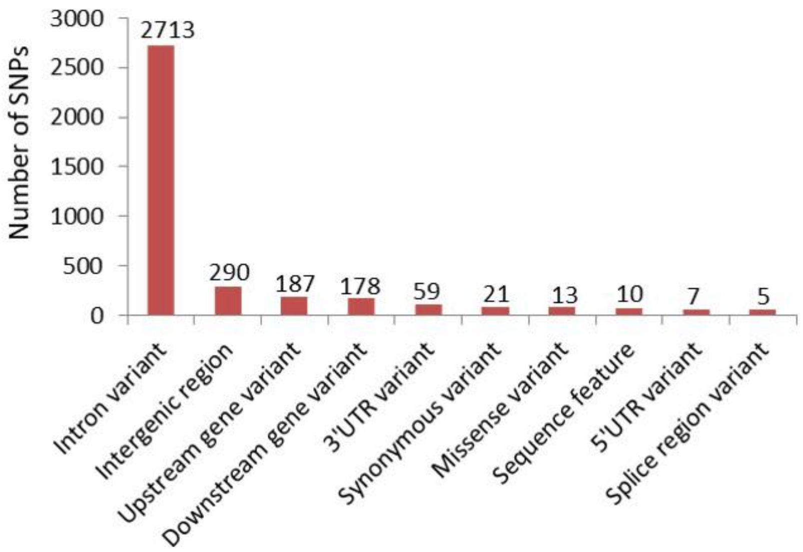

Supplementary Figure 33. Variants annotation of 3,483 SNPs shared by SCZ and at least one phenotype of smoking behavior. We used snpEff (http://snpeff.sourceforge.net/) to carry out the annotation analysis.

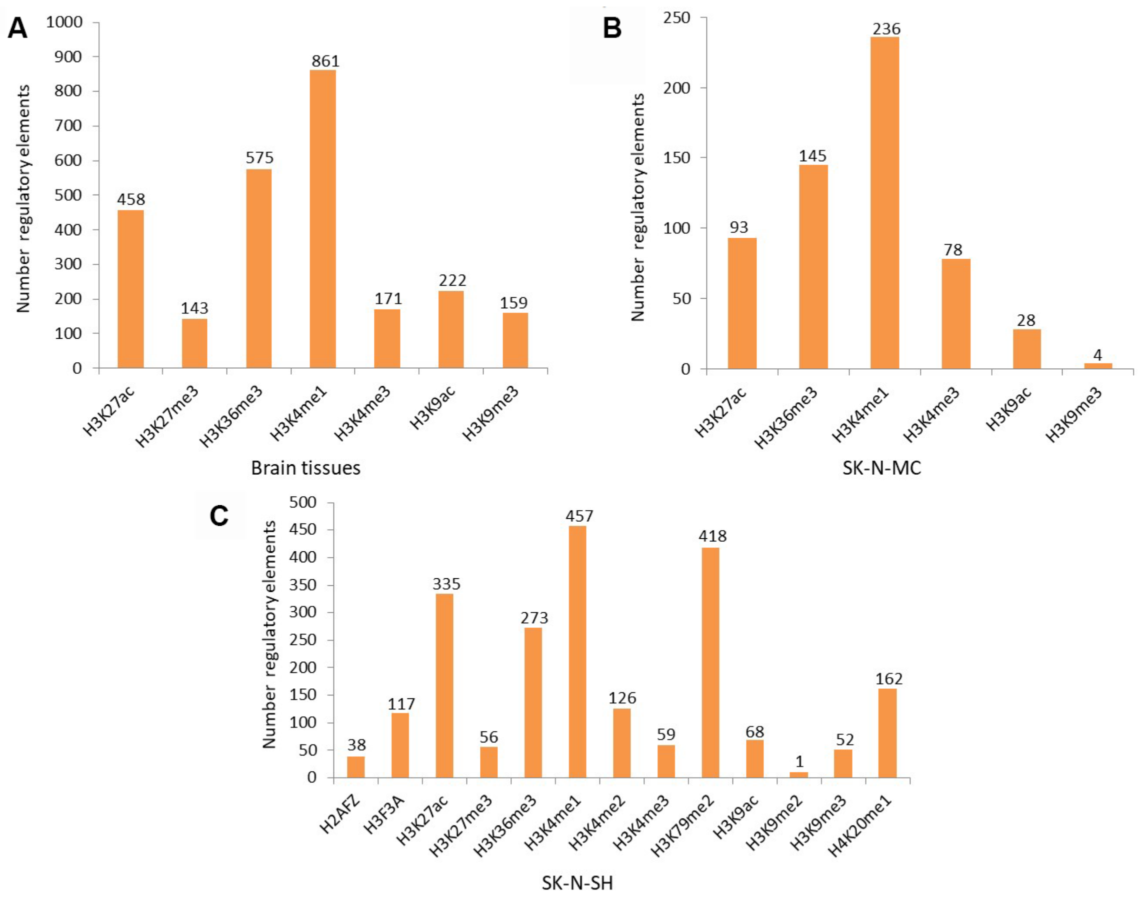

Supplementary Figure 34. 3843 SNPs shared by SCZ and smoking located within different types of regulatory elements in brain tissues and neuroblastoma cell lines. (**A**) For brain tissues. (**B**) for SK-N-MC cells. (**C**) for SK-N-SH. The data of different types of regulatory elements, including H2AFZ, H3F3A, H3K27ac, H3K27me3, H3K36me3, H3K4me1, H3K4me2, H3K4me3, H3K79me2, H3K9ac, H3K9me2, H3K9me3, and H4K20me1, were downloaded from ENCODE database (https://www.encodeproject.org/).

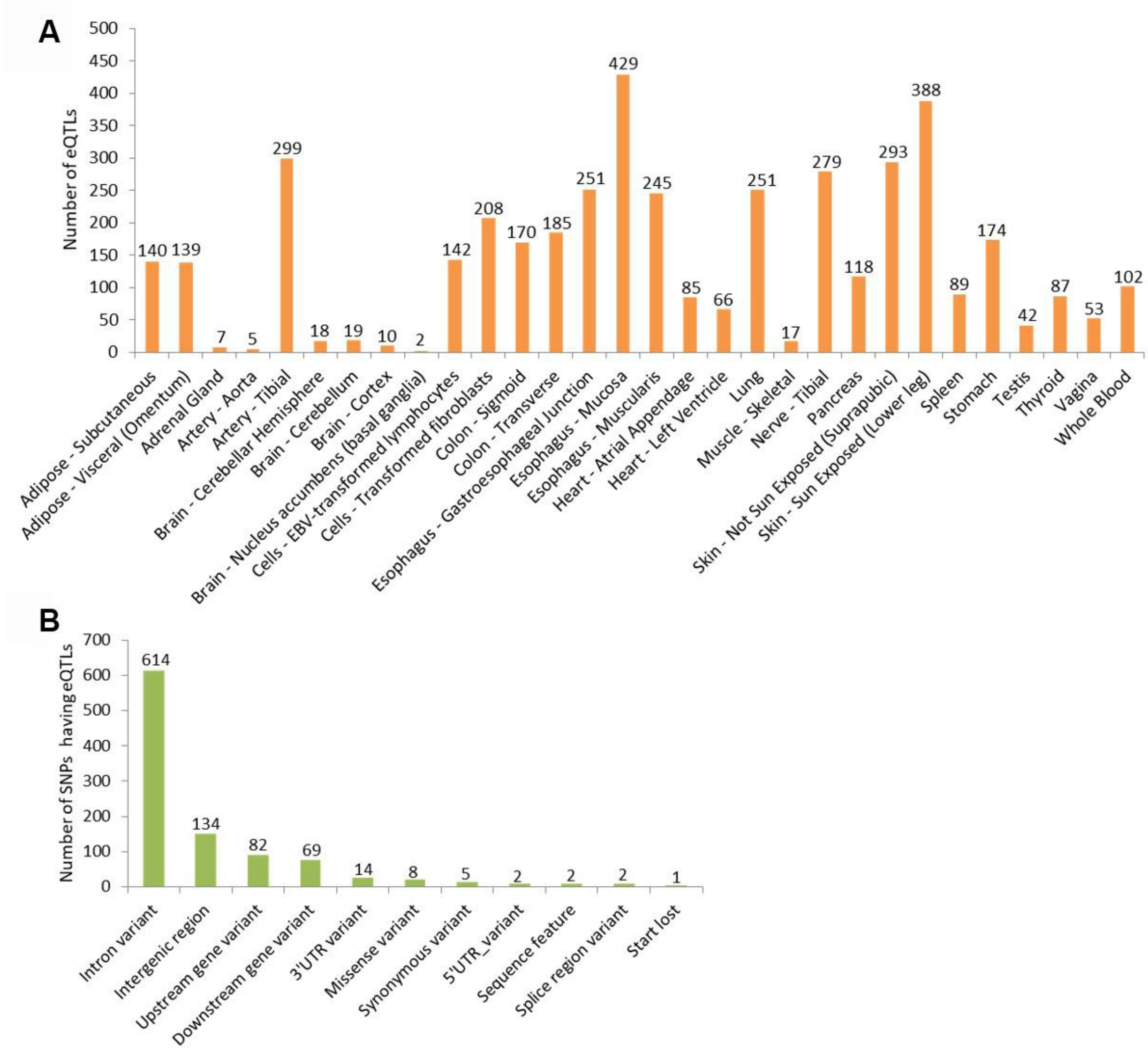

Supplementary Figure 35. 933 of 3843 SNPs shared by SCZ and smoking having 4313 eQTLs in 31 human tissues. (**A**) 4313 eQTLs distributed in 31 human tissues. (**B**) Variant annotation of 933 SNPs shared by SCZ and at least one phenotype of smoking behavior. We used the tool snpEff (http://snpeff.sourceforge.net/) to carry out the annotation analysis. Based on the tissue-specific eQTL association data from the GTEx database, 933 of 3,483 SNPs have *cis*-regulatory effects on gene expression with 4,313 SNP-eQTLs pairs in 31 human tissues.

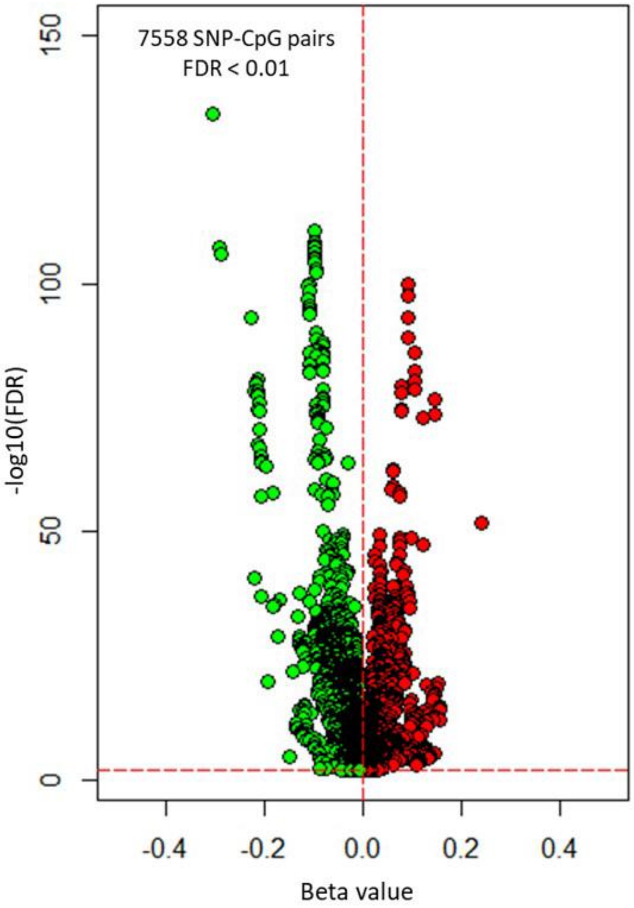

Supplementary Figure 36. Volcano plot for significant SNP–CpG methylation associations in brain samples. Green represents these significant SNP-CpG pairs with negative beta value. Red represents these significant SNP-CpG pairs with positive beta value.
