# Supplementary material for: Identification of 34 genes conferring genetic and pharmacological risk for the comorbidity of schizophrenia and smoking behaviors
Source: Aging (Albany NY). 2020 Feb 3;12(3):2169–225. doi: 10.18632/aging.102735 (PMC7041787; doi:10.18632/aging.102735)
Supplement: Supplementary Table 1 [file aging-12-102735-s001..docx]

**Supplementary Table 1. Multiple omics datasets used in current study downloaded from public databases.**

| **Dataset Name** | **Sample Size** | **Description** | **URLs** |
| --- | --- | --- | --- |
| GWAS data on SCZ | 79,845 | This dataset was obtained from a published GWAS of SCZ [1]. This study contains 49 ancestry matched, non-overlapping case-control samples (46 of European and 3 of East Asian samples, 34,241 cases and 45,604 controls) and 3 European family-based samples (1,235 parent affected-offspring trios). Written informed consent was provided by all participants. Due to these included samples obtained from various studies, both the criteria for Diagnostic and Statistical Manual of Mental Disorders (DSM) and International Classification of Diseases (ICD) were applied in the diagnosis of the patients. All the genotype data were processed by the Psychiatric Genomics Consortium (PGC) using unified quality control procedures. All genomic coordinates were defined with the use of the Human Genome references 19. | https://www.med.unc.edu/pgc/downloads |
| GWAS data on smoking | 74,053 | This dataset was a published meta-GWAS on smoking behaviors by the Tobacco and Genetics Consortium (TAG) [2]. This study obtained genotype data from 16 European-based participating cohorts with a sample size of 74,053. The TAG samples were surveyed mainly between 1970 and 2006. All the participants from all cohorts of the TAG consortium provided informed consent. Genome-wide genotype data on smoking status (ever/never smoked regularly), quantity smoked in ever regular smokers, smoking cessation, and age at initiation were available for N = 69,409 individuals, N = 38,181 individuals, N = 35,845 individuals, N = 22,438 individuals, respectively. The SNP positions from the Human Genome references 18 were lifted to the Human Genome references 19. | https://www.med.unc.edu/pgc/downloads |
| GWAS data on height | 183,727 | To confirm the identified enriched pathways were due to shared disorder biology, we repeated the GWAS enrichment analysis of a GWAS of human height (N = 183,727) [3] as a negative control. The phenotype of human height was not thought to share genetic aetiology with SCZ and smoking behaviors. | http://portals.broadinstitute.org/collaboration/giant/index.php/GIANT_consortium_data_files |
| GWAS data on null | 3,960 | To confirm the identified enriched pathways were due to shared disorder biology, we repeated the GWAS enrichment analysis of null GWAS as a negative control. This null GWAS based on randomly distributed phenotypes that we constructed from a real GWAS with a sample size of 3,960 [4] to make sure that the detected pathways were attributable to underlying genetic factors instead of artifacts of the analytical methods. Since power is not an issue for the defined null sample which contains no true effects, the relatively small sample size is not important. | http://www.ncbi.nlm.nih.gov/sites/enteez?Db=gap |
| Expression data on brain development and aging | 1,340 | The development of the human brain is a complex and precisely regulated process that unfolds over a protracted period of time. Human-specific features of this process, especially the ways in which highly complex neural circuits of the cerebral cortex form, are likely to be important factors in the evolution of human specializations. The BrainSpan exon array data related to brain development and aging were downloaded from the NCBI’s Gene Expression Omnibus (GEO; Accession No. GSE25219). The generation and analysis of genome-wide exon-level transcriptome data from 16 brain regions comprising the cerebellar cortex, mediodorsal nucleus of the thalamus, striatum, amygdala, hippocampus, and 11 areas of the neocortex. The dataset was generated from 1,340 samples collected from both hemispheres of 57 postmortem human brains, spanning from embryonic development to late adulthood and representing males and females of multiple ethnicities. | https://www.ncbi.nlm.nih.gov/geo/download/?acc=GSE25219 |
| Methylation data on smoking | 18,677 | This dataset was systematically collected in our previous reported study [5]. To identify all studies on the association of cigarette smoking with alterations in DNAm, a total of 1,447 studies published prior to Jun 13, 2015, were retrieved from the PubMed database. The key words used for the search were “smoking,” “smoke,” “tobacco,” “nicotine,” “cigarette,” and “methylation.” Following a systematic and strict procedure, a number of 28 studies were identified. Of them, 26 studies were from 17,675 blood samples and two studies were from 1,002 buccal samples. A total of 1,429 significantly smoking-associated methylation genes were identified. Among these genes, there were a number of 320 genes whose relevance is supported by at least two independent pieces of evidence. | https://www.nature.com/articles/s41598-017-01856-4 |
| Methylation data on SCZ based on blood samples | 847 | To examine whether these module genes (N = 149) related to smoking-associated methylation were significantly methylated in schizophrenia patients, we downloaded the methylation data [6] from the database of NCBI’s Gene Expression Omnibus (GEO; Accession number GSE84727). 847 whole blood derived DNA samples (414 schizophrenia cases and 433 controls) representing phase 2 of our meta-analysis. Bisulfite converted DNA was hybridized to the Illumina Infinium 450k Human Methylation Beadchip v1.0. A total of 8,236 CpG loci were annotated to these 149 smoking-associated methylated genes. | https://www.ncbi.nlm.nih.gov/geo/download/?acc=GSE84727 |
| Methylation data on SCZ based on brain samples | 258 | DNA methylation (DNAm) is important in brain development, and potentially in schizophrenia. To validate the *cis*-regulatory effects of SNPs in 34 candidate genes on DNA methylation in blood samples, we downloaded *cis*-meQTL data in human brain samples [7] from the database of NCBI’s Gene Expression Omnibus (GEO; Accession No. GSE74193). DNA methylation data from the dorsolateral prefrontal cortex from 335 controls and 191 patients with schizophrenia. Some samples were run multiple times. | https://www.ncbi.nlm.nih.gov/geo/download/?acc=GSE74193 |
| Expression data on SCZ and smoking based on olfactory epithelium tissues | 31 | Expression patterns of genes are vital in revealing any biological system. Several research groups have reported that olfactory epithelium containing olfactory receptor neurons represents a good surrogate for fetal and adult brain.[8-10]. Thus, we performed a differential gene expression analysis to explore the expression profiles of candidate genes in olfactory epithelium tissues among SCZ patients and controls grouped by smoking status (N = 31). The expression data [8] were downloaded from the GEO database (Accession No. GSE73129). Differences between SCZ patients and controls grouped by smoking status were compared for significance with ANOVA. The Turkey HSD test was used for multiple comparisons among different groups. A P value < 0.05 was considered statistically significant. | https://www.ncbi.nlm.nih.gov/geo/download/?acc=GSE73129 |
| Expression data on SCZ based on human induced pluripotent stem cells (hiPSCs) | 8 | Human induced pluripotent stem cells (hiPSCs) provide a novel strategy for defining characteristics of schizophrenic neurons. Analysis of induced pluripotent stem cells (iPSCs) generated from schizophrenic patients and differentiated to iPSC-derived neurons in vitro. Thus, we used the first cell-based human model of schizophrenia [11] by directly reprogramming fibroblasts from schizophrenic patients into hiPSCs and subsequently differentiating these disorder-specific hiPSCs into neurons *in vitro* to explore the different expression profiles of 34 candidate genes between control (N = 4) and schizophrenic patients (N = 4). Three biological replicates were undertaken for each control and schizophrenic patient. The gene expression data of schizophrenic hiPSC-derived neurons were downloaded from the GEO database (Accessions #: GSE25673). Student’s t-test was used for comparing the difference between patients and controls. | https://www.ncbi.nlm.nih.gov/geo/download/?acc=GDS3938 |
| Pharmacogenomics data on quetiapine-dosage treatment | 20 | To reveal the potential druggable genes that may initiate therapeutic effects, candidate genes expression profiling of quetiapine-dosage induced alterations in the mouse brain was undertaken. We downloaded the psychotropic drug-treated gene expression data from NCBI’s GEO with Accession NOs of GSE45229. Mice were given one of 3 treatments (vehicle, 100 mg/kg quetiapine, 10 mg/kg quetiapine) [12]. Pooled tissue samples were used for microarray analysis of gene expression in striatum. One-way ANOVA analysis was applied and Tukey-Kramer HSD test was used for multiple comparisons. | https://www.ncbi.nlm.nih.gov/geo/query/acc.cgi?acc=GSE45229 |
| Pharmacogenomics data on nicotine-dosage treatment | 25 | To reveal the potential druggable genes that may initiate therapeutic effects, candidate genes expression profiling of nicotine-dosage induced alterations in the mouse brain was undertaken. we downloaded gene expression data from the GEO dataset (Accession #: GES50254) to reveal the nicotine dosage-induced changes of candidate genes in rats exposed for 28 days to filtered air (sham), or to a low (8 ug nicotine/l), medium (15 ug nicotine/l), or high (23 ug nicotine/l) concentration of nicotine [13]. One-way ANOVA analysis was applied and Tukey-Kramer HSD test was used for multiple comparisons. | https://www.ncbi.nlm.nih.gov/geo/download/?acc=GDS5063 |
| Pharmacogenomics data on the time-course treatment of psychotropic drugs (#1) | 156 | To identify the molecular mechanisms that may initiate therapeutic effects, whole-genome expression profiling (Illumina Mouse WG-6 microarrays) of drug-induced alterations in the mouse brain was undertaken, with a focus on the time-course (1, 2, 4 and 8h) of gene expression changes produced by eighteen major psychotropic drugs. The microarray experiment was performed to analyze time-course of drug-induced transcriptional response in C57BL/6J mouse striatum. Three antidepressants (bupropion 20 mg/kg, tranylcypromine 20 mg/kg, mianserin 20 mg/kg, i.p.), three anxiolytics (diazepam 5 mg/kg, buspirone 10 mg/kg, hydroxyzine 10 mg/kg, i.p.), and three antipsychotics (clozapine 3 mg/kg, risperidone 0.5 mg/kg, haloperidol 1 mg/kg) were selected for the comparison. | https://www.ncbi.nlm.nih.gov/geo/download/?acc=GSE48954 |
| Pharmacogenomics data on the time-course treatment of psychotropic drugs (#2) | 108 | The microarray experiment was performed to analyze time-course of drug-induced transcriptional response in C57BL/6J mouse striatum. Six the most addictive and harming drugs of abuse (morphine 20 mg/kg, heroin 10 mg/kg, ethanol 2 g/kg, nicotine 1 mg/kg, methamphetamine 2 mg/kg or cocaine 25 mg/kg, i.p.) were selected for the comparison. | https://www.ncbi.nlm.nih.gov/geo/download/?acc=GSE15774 |
| Pharmacogenomics data on the time-course treatment of psychotropic drugs (#3) | 60 | To identify the molecular mechanisms that may initiate therapeutic effects, whole-genome expression profiling (Illumina Mouse WG-6 microarrays) of drug-induced alterations in the mouse brain was undertaken, with a focus on the time-course (1, 2, 4 and 8h) of gene expression changes produced by eighteen major psychotropic drugs. The microarray experiment was performed to analyze time-course of drug-induced transcriptional response in C57BL/6J mouse striatum. Three antidepressants (imipramine 10 mg/kg, fluoxetine 20 mg/kg and tianeptine 20 mg/kg, i.p.) were selected for the comparison. | https://www.ncbi.nlm.nih.gov/geo/download/?acc=GSE48951 |
