# Supplementary material for: Identification of 34 genes conferring genetic and pharmacological risk for the comorbidity of schizophrenia and smoking behaviors
Source: Aging (Albany NY). 2020 Feb 3;12(3):2169–225. doi: 10.18632/aging.102735 (PMC7041787; doi:10.18632/aging.102735)
Supplement: Supplementary Table 2 [file aging-12-102735-s004..docx]

**Supplementary Table 2. 84 significantly enriched pathways shared between schizophrenia and smoking behaviors.**

| **Pathway ID** | **Description** | **SCZ P-value** | **CPD P-value** | **Ever smoking P-value** | **Former smoking P-value** | **Age at smoking initiation P-value** |
| --- | --- | --- | --- | --- | --- | --- |
| GO:0014069 | Postsynaptic density | 0 | **1.43E-13** | **1.21E-09** | **1.50E-06** | **3.09E-06** |
| GO:0005891 | Voltage-gated calcium channel complex | 3.04E-14 | 0.1377 | **0.0091** | 0.0225 | **0.0027** |
| GO:0005245 | Voltage-gated calcium channel activity | 4.66E-13 | 0.0400 | **0.0006** | **0.0008** | **0.0004** |
| GO:0045211 | Postsynaptic membrane | 7.44E-13 | **4.97E-11** | **1.10E-10** | **5.92E-09** | **5.52E-06** |
| GO:0043025 | Neuronal cell body | 1.39E-11 | **1.67E-07** | **1.68E-05** | **0.0001** | 0.0083 |
| GO:0005244 | Voltage-gated ion channel activity | 1.21E-09 | 0.0094 | **4.73E-05** | **8.13E-07** | **7.19E-06** |
| GO:0045202 | Synapse | 3.01E-09 | **2.76E-13** | **6.88E-15** | **1.91E-09** | **4.88E-08** |
| GO:0030425 | Dendrite | 9.46E-09 | **1.34E-07** | **0.0043** | **0.0011** | **0.0001** |
| GO:0007268 | Synaptic transmission | 3.22E-08 | **8.03E-10** | **1.85E-11** | **3.22E-11** | **9.84E-06** |
| GO:0007411 | Axon guidance | 5.89E-08 | **8.28E-06** | **6.04E-12** | **8.10E-12** | **3.64E-09** |
| GO:0015464 | Acetylcholine receptor activity | 7.92E-08 | **0** | 0.3912 | 0.0131 | 0.0937 |
| hsa04720 | Long-term potentiation | 1.79E-07 | 0.0991 | **0.0003** | **0.0087** | **0.0002** |
| GO:0005001 | Transmembrane receptor protein tyrosine phosphatase activity | 7.21E-07 | **0.0010** | **5.50E-08** | **0.0086** | **7.84E-05** |
| GO:0006816 | Calcium ion transport | 7.31E-07 | 0.0133 | **1.75E-05** | **0.0001** | **2.01E-06** |
| GO:0042734 | Presynaptic membrane | 1.33E-06 | 0.0186 | **3.12E-06** | **0.0004** | **0.0003** |
| GO:0006112 | Energy reserve metabolic process | 1.33E-06 | 0.0333 | **3.53E-05** | 0.0684 | **4.26E-05** |
| hsa05414 | Dilated cardiomyopathy | 3.13E-06 | 0.0237 | **2.76E-05** | 0.0270 | **0.0025** |
| GO:0043197 | Dendritic spine | 4.34E-06 | **0.0009** | **0.0007** | **0.0011** | **0.0048** |
| hsa05410 | Hypertrophic cardiomyopathy (HCM) | 5.15E-06 | 0.0550 | **0.0026** | 0.0231 | 0.0163 |
| hsa04010 | MAPK signaling pathway | 8.24E-06 | 0.0081 | **0.0009** | **0.0076** | **0.0006** |
| hsa05412 | Arrhythmogenic right ventricular cardiomyopathy (ARVC) | 1.06E-05 | **0.0025** | **4.17E-06** | **1.36E-05** | **0.0008** |
| GO:0060079 | regulation of excitatory postsynaptic membrane potential | 1.13E-05 | **0** | 0.2769 | **5.17E-06** | 0.0407 |
| GO:0050796 | regulation of insulin secretion | 1.38E-05 | 0.0071 | **0.0031** | 0.1553 | **0.0003** |
| R-HSA-2467813 | Separation of Sister Chromatids | 2.18E-05 | **4.89E-06** | 0.1230 | 0.8301 | 0.6372 |
| GO:0048041 | focal adhesion assembly | 6.84E-05 | 0.0410 | 0.4262 | 0.1181 | **0.0052** |
| GO:0004889 | nicotinic acetylcholine-activated cation-selective channel activity | 6.89E-05 | **0** | 0.3010 | 0.0280 | 0.1518 |
| hsa04912 | GnRH signaling pathway | 7.76E-05 | **0.0056** | **8.06E-05** | **0.0042** | **0.0001** |
| GO:0005516 | calmodulin binding | 9.23E-05 | **0.0008** | **4.99E-06** | **1.23E-06** | 0.0122 |
| GO:0008066 | glutamate receptor activity | 9.41E-05 | **0.0040** | 0.0142 | **0.0007** | **0.0001** |
| GO:0030315 | T-tubule | 9.87E-05 | 0.1611 | **0.0055** | 0.1042 | **0.0024** |
| GO:0045296 | cadherin binding | 1.44E-04 | **0.0028** | **0.0008** | **0.0004** | **5.83E-05** |
| cxcr4Pathway | CXCR4 Signaling Pathway | 1.49E-04 | **0.0013** | 0.070 | 0.0196 | 0.2016 |
| GO:0005892 | nicotinic acetylcholine-gated receptor-channel complex | 2.03E-04 | **0** | 0.3836 | 0.0592 | 0.1342 |
| GO:0005096 | GTPase activator activity | 2.60E-04 | **0.0002** | **4.91E-05** | **0.0007** | 0.0069 |
| GO:0001764 | neuron migration | 2.78E-04 | 0.1975 | **0.0028** | 0.0115 | 0.0854 |
| GO:0048167 | regulation of synaptic plasticity | 3.55E-04 | **0.0034** | 0.0765 | **0.0020** | 0.0991 |
| GO:0046982 | protein heterodimerization activity | 3.99E-04 | **0.0011** | **0.0003** | **0.0047** | 0.0703 |
| R-HSA-5632684 | Hedgehog 'on' state | 4.04E-04 | **6.71E-12** | 0.8588 | 0.6504 | 0.5780 |
| GO:0004697 | protein kinase C activity | 4.76E-04 | 0.2579 | **0.0023** | 0.0110 | **6.67E-05** |
| GO:0001508 | regulation of action potential | 4.88E-04 | **0** | 0.0716 | 0.0262 | 0.1443 |
| GO:0007156 | homophilic cell adhesion | 4.91E-04 | **0.0011** | **1.48E-05** | **2.00E-07** | **1.50E-07** |
| GO:0005216 | ion channel activity | 5.09E-04 | **2.95E-13** | **0.0001** | **1.29E-05** | **0.0001** |
| GO:0051056 | regulation of small GTPase mediated signal transduction | 5.30E-04 | **0.0002** | **1.14E-06** | **4.88E-05** | **0.0002** |
| hsa04020 | Calcium signaling pathway | 5.96E-04 | **0.0056** | **2.74E-10** | **2.38E-06** | **2.99E-06** |
| GO:0015297 | antiporter activity | 6.05E-04 | 0.1069 | 0.1549 | **0.0016** | 0.0107 |
| GO:0006813 | potassium ion transport | 7.43E-04 | **0.0038** | **2.73E-06** | **0.0017** | **9.14E-05** |
| GO:0005234 | extracellular-glutamate-gated ion channel activity | 9.05E-04 | **0.0043** | 0.0588 | **0.0050** | **0.0037** |
| biopeptidesPathway | Bioactive Peptide Induced Signaling Pathway | 0.00105 | 0.2457 | 0.2987 | **0.0057** | 0.0545 |
| GO:0007413 | axonal fasciculation | 0.0011 | 0.1479 | 0.0300 | **0.0083** | 0.1062 |
| GO:0007420 | brain development | 0.0011 | 0.0204 | **0.0038** | **0.0040** | 0.0295 |
| GO:0007626 | locomotory behavior | 0.00115 | **9.36E-14** | **0.0032** | **3.10E-05** | **0.0030** |
| gpcrPathway | Signaling Pathway from G-Protein Families | 0.00131 | 0.0139 | 0.0399 | 0.2843 | **0.0036** |
| GO:0008013 | beta-catenin binding | 0.00151 | 0.0250 | **0.0015** | **0.0002** | 0.0082 |
| GO:0001701 | in utero embryonic development | 0.00157 | 0.0332 | **0.0005** | 0.0448 | 0.2770 |
| GO:0008076 | voltage-gated potassium channel complex | 0.00163 | 0.0209 | **0.0004** | **0.0036** | **0.0046** |
| GO:0016477 | cell migration | 0.002 | **0.0034** | 0.3172 | 0.2692 | 0.0396 |
| GO:0006812 | cation transport | 0.0020 | 0.3269 | 0.0701 | **0.0001** | **0.0018** |
| GO:0005085 | guanyl-nucleotide exchange factor activity | 0.00214 | 0.0070 | **3.88E-07** | **0.0002** | **3.62E-05** |
| GO:0030426 | growth cone | 0.00220 | 0.1866 | **0.0001** | 0.0142 | 0.0300 |
| GO:0048011 | nerve growth factor receptor signaling pathway | 0.00238 | 0.1766 | **0.0056** | **8.20E-05** | **7.36E-05** |
| GO:0046928 | regulation of neurotransmitter secretion | 0.00242 | **0** | 0.6366 | **0.0009** | 0.2304 |
| GO:0023034 | intracellular signaling pathway | 0.00253 | 0.0446 | **0.0002** | **1.39E-06** | **0.0001** |
| GO:0042493 | response to drug | 0.00321 | **4.14E-07** | 0.0360 | 0.0314 | 0.2751 |
| GO:0007205 | activation of protein kinase C activity by G-protein coupled receptor protein signaling pathway | 0.00327 | 0.5082 | **0.0064** | 0.2543 | 0.2388 |
| GO:0009986 | cell surface | 0.00339 | 0.0082 | **0.0007** | 0.0518 | 0.0075 |
| GO:0031225 | anchored to membrane | 0.00352 | 0.0965 | 0.1650 | **0.0025** | 0.0086 |
| GO:0030165 | PDZ domain binding | 0.00378 | 0.0238 | **0.0005** | 0.0208 | 0.0094 |
| GO:0019228 | regulation of action potential in neuron | 0.00411 | 0.0172 | 0.2668 | **0.0017** | **2.56E-05** |
| hsa04730 | Long-term depression | 0.00417 | **7.10E-05** | **0.0012** | **2.85E-06** | **0.0009** |
| agrPathway | Agrin in Postsynaptic Differentiation | 0.00427 | 0.0111 | 0.0213 | **0.0063** | **0.0004** |
| edg1Pathway | Phospholipids as signalling intermediaries | 0.00481 | 0.0505 | **0.0047** | 0.0236 | 0.0264 |
| calcineurinPathway | Effects of calcineurin in Keratinocyte Differentiation | 0.00499 | 0.0461 | 0.2350 | 0.2321 | **0.0017** |
| GO:0007409 | axonogenesis | 0.0050 | 0.0225 | **3.39E-05** | **0.0067** | **0.0027** |
| GO:0004629 | phospholipase C activity | 0.00506 | 0.2683 | 0.0704 | **0.0004** | 0.4306 |
| R-HSA-4641258 | Degradation of DVL | 0.00512 | **9.19E-14** | 0.8883 | 0.5507 | 0.2936 |
| fcer1Pathway | Fc Epsilon Receptor I Signaling in Mast Cells | 0.00513 | 0.1880 | 0.3738 | 0.2656 | **0.0002** |
| GO:0007264 | small GTPase mediated signal transduction | 0.00538 | 0.1142 | **0.0024** | 0.0510 | **0.0007** |
| GO:0051539 | 4 iron, 4 sulfur cluster binding | 0.00604 | **2.00E-14** | 0.3257 | 0.2802 | 0.7022 |
| sppaPathway | Aspirin Blocks Signaling Pathway Involved in Platelet Activation | 0.00623 | **0.0010** | **0.0047** | **0.0050** | 0.0179 |
| R-HSA-1234176 | Oxygen-dependent proline hydroxylation of Hypoxia-inducible Factor Alpha | 0.00627 | **7.39E-14** | 0.5995 | 0.6921 | 0.4570 |
| GO:0007194 | negative regulation of adenylate cyclase activity | 0.0064 | 0.1784 | **0.0044** | 0.0520 | 0.0978 |
| GO:0016529 | sarcoplasmic reticulum | 0.0065 | 0.0701 | **0.0008** | 0.2641 | 0.0639 |
| hsa04270 | Vascular smooth muscle contraction | 0.00656 | 0.0132 | **1.46E-05** | **3.24E-05** | **4.05E-06** |
| GO:0048511 | rhythmic process | 0.00661 | 0.2534 | **0.00083** | 0.0365 | **0.0023** |
