# Supplementary material for: Identification of 34 genes conferring genetic and pharmacological risk for the comorbidity of schizophrenia and smoking behaviors
Source: Aging (Albany NY). 2020 Feb 3;12(3):2169–225. doi: 10.18632/aging.102735 (PMC7041787; doi:10.18632/aging.102735)
Supplement: Supplementary Table 3 [file aging-12-102735-s003..docx]

**Supplementary Table 3. Shared genes between schizophrenia and smoking behaviors.**

| **Gene name** | **Chr** | **Number of SNPs** | **Z-score** | **SCZ P-value** | **CPD P-value** | **Ever smoking P-value** | **Former smoking P-value** | **Age at smoking initiation P-value** | **Located in previous identified 108 loci** |
| --- | --- | --- | --- | --- | --- | --- | --- | --- | --- |
| *MAS1L* | 6 | 30 | 10.605 | 1.42E-26 | 0.6331 | 0.6046 | **0.0157** | **0.0308** | No |
| *UBD* | 6 | 25 | 10.511 | 3.86E-26 | 0.6688 | **0.0387** | 0.1572 | 0.1194 | No |
| *OR2H1* | 6 | 21 | 9.7446 | 9.73E-23 | 0.7252 | 0.7495 | 0.3210 | **0.0115** | No |
| *HIST1H2AK* | 6 | 76 | 9.7397 | 1.02E-22 | 0.3726 | 0.5180 | **0.0202** | 0.1753 | No |
| *DPCR1* | 6 | 65 | 9.6674 | 2.07E-22 | 0.0741 | 0.1099 | **0.0220** | 0.6184 | No |
| *HIST1H2BM* | 6 | 88 | 9.6435 | 2.62E-22 | 0.6007 | 0.7593 | **0.0081** | 0.2323 | No |
| *HIST1H2AJ* | 6 | 88 | 9.6302 | 2.98E-22 | 0.6007 | 0.7593 | **0.0081** | 0.2323 | No |
| *HIST1H2BN* | 6 | 113 | 9.6241 | 3.16E-22 | 0.5418 | 0.6973 | **0.0134** | 0.1430 | No |
| *HIST1H4K* | 6 | 83 | 9.4741 | 1.35E-21 | 0.4945 | 0.5206 | **0.0219** | 0.1380 | No |
| *HIST1H4J* | 6 | 83 | 9.31 | 6.39E-21 | 0.4767 | 0.6659 | **0.0126** | 0.1392 | No |
| *HIST1H3H* | 6 | 90 | 9.2984 | 7.13E-21 | 0.6021 | 0.7786 | **0.0065** | 0.2525 | No |
| *HIST1H2AI* | 6 | 87 | 9.2646 | 9.79E-21 | 0.6185 | 0.7875 | **0.0061** | 0.3117 | No |
| *HIST1H2BL* | 6 | 89 | 9.1516 | 2.81E-20 | 0.6167 | 0.8008 | **0.0054** | 0.3344 | No |
| *ZSCAN31* | 6 | 215 | 8.9682 | 1.51E-19 | 0.8323 | 0.3326 | **0.0414** | 0.2337 | Yes |
| *VWA7* | 6 | 117 | 8.8527 | 4.27E-19 | 0.1379 | 0.1518 | **0.0027** | 0.3870 | No |
| *NOTCH4* | 6 | 323 | 8.8205 | 5.70E-19 | 0.8018 | 0.4215 | **0.0305** | 0.3826 | No |
| *OR5V1* | 6 | 34 | 8.8084 | 6.35E-19 | 0.4038 | **0.0042** | **0.0038** | 0.0559 | No |
| *VARS* | 6 | 148 | 8.802 | 6.73E-19 | 0.1924 | 0.1580 | **0.0132** | 0.4349 | No |
| *MSH5* | 6 | 136 | 8.6521 | 2.53E-18 | 0.1071 | 0.1875 | **0.0032** | 0.4823 | No |
| *HIST1H2AL* | 6 | 86 | 8.6298 | 3.07E-18 | 0.6282 | 0.6799 | **0.0154** | 0.1516 | No |
| *HIST1H1B* | 6 | 89 | 8.614 | 3.53E-18 | 0.6241 | 0.7234 | **0.0158** | 0.1466 | No |
| *AIF1* | 6 | 170 | 8.6131 | 3.56E-18 | 0.3966 | 0.7153 | **0.0095** | 0.5243 | No |
| *AGER* | 6 | 121 | 8.5656 | 5.38E-18 | 0.4915 | 0.2177 | **0.0382** | 0.5523 | No |
| *SAPCD1* | 6 | 97 | 8.5252 | 7.63E-18 | 0.1245 | 0.1058 | **0.0040** | 0.4434 | No |
| *AGPAT1* | 6 | 127 | 8.5014 | 9.36E-18 | 0.4676 | 0.2319 | **0.0443** | 0.6283 | No |
| *VARS2* | 6 | 36 | 8.4932 | 1.00E-17 | 0.1939 | **0.0314** | **0.0346** | 0.3516 | No |
| *HLA-B* | 6 | 1655 | 8.2895 | 5.69E-17 | **0.0108** | 0.2955 | **0.0009** | **0.0154** | No |
| *PGBD1* | 6 | 140 | 8.2838 | 5.97E-17 | 0.5432 | 0.1417 | **0.0165** | 0.6108 | No |
| *ZKSCAN4* | 6 | 118 | 8.2805 | 6.13E-17 | 0.8263 | 0.1017 | **0.0040** | 0.4964 | No |
| *AS3MT* | 10 | 218 | 8.2757 | 6.38E-17 | 0.3905 | **0.0005** | 0.9644 | 0.8576 | Yes |
| *GTF2H4* | 6 | 30 | 8.2752 | 6.41E-17 | 0.2193 | **0.0285** | **0.0300** | 0.3554 | No |
| *PBX2* | 6 | 137 | 8.2726 | 6.55E-17 | 0.5030 | 0.2184 | **0.0278** | 0.5774 | No |
| *GPSM3* | 6 | 138 | 8.2095 | 1.11E-16 | 0.5778 | 0.2398 | **0.0170** | 0.5271 | No |
| *PRRC2A* | 6 | 211 | 8.1769 | 1.46E-16 | 0.5658 | 0.7091 | **0.0078** | 0.6285 | No |
| *ZSCAN26* | 6 | 116 | 8.1483 | 1.85E-16 | 0.6166 | 0.1399 | **0.0072** | 0.6641 | No |
| *C10orf32* | 10 | 155 | 8.1015 | 2.71E-16 | 0.2722 | **0.0003** | 0.9286 | 0.7770 | Yes |
| *HIST1H4L* | 6 | 87 | 8.0529 | 4.04E-16 | 0.6271 | 0.4897 | **0.0227** | 0.1442 | No |
| *HIST1H3I* | 6 | 88 | 8.0503 | 4.13E-16 | 0.6271 | 0.4897 | **0.0227** | 0.1442 | No |
| *BAG6* | 6 | 190 | 7.9352 | 1.05E-15 | 0.5527 | 0.7017 | **0.0150** | 0.7763 | No |
| *NKAPL* | 6 | 85 | 7.8989 | 1.41E-15 | 0.7847 | 0.0898 | **0.0023** | 0.5308 | No |
| *C6orf10* | 6 | 474 | 7.7769 | 3.72E-15 | 0.4144 | 0.3814 | **0.0112** | 0.7839 | No |
| *NT5C2* | 10 | 372 | 7.6871 | 7.52E-15 | 0.2312 | **0.0013** | 0.8202 | 0.8099 | Yes |
| *DDR1* | 6 | 29 | 7.6306 | 1.17E-14 | 0.2776 | **0.0379** | **0.0386** | 0.1908 | No |
| *CNNM2* | 10 | 515 | 7.5838 | 1.68E-14 | 0.2181 | **0.0011** | 0.9003 | 0.8029 | Yes |
| *OR14J1* | 6 | 62 | 7.556 | 2.08E-14 | 0.3436 | 0.0698 | **0.0047** | **0.0464** | No |
| *C6orf48* | 6 | 141 | 7.5012 | 3.16E-14 | 0.0960 | 0.1005 | **0.0308** | 0.4516 | No |
| *OR12D3* | 6 | 76 | 7.4931 | 3.36E-14 | 0.3306 | 0.0606 | **0.0058** | 0.1959 | No |
| *LOC101929163* | 6 | 252 | 7.4745 | 3.87E-14 | 0.6178 | 0.4619 | **0.0120** | 0.8385 | No |
| *HIST1H3J* | 6 | 92 | 7.472 | 3.95E-14 | 0.5343 | 0.3126 | **0.0371** | 0.1600 | No |
| *APOM* | 6 | 150 | 7.4558 | 4.47E-14 | 0.5194 | 0.6871 | **0.0181** | 0.7700 | No |
| *BTN1A1* | 6 | 143 | 7.4317 | 5.36E-14 | 0.6993 | 0.1471 | 0.6591 | **0.0042** | No |
| *ZNRD1* | 6 | 4 | 7.4221 | 5.76E-14 | 0.0990 | 0.2897 | **0.0206** | 0.6668 | No |
| *HSPA1B* | 6 | 140 | 7.4069 | 6.46E-14 | 0.1034 | 0.1251 | **0.0449** | 0.4621 | No |
| *PPP1R11* | 6 | 5 | 7.3549 | 9.55E-14 | 0.0913 | 0.2264 | **0.0176** | 0.7033 | No |
| *RNF39* | 6 | 5 | 7.3549 | 9.55E-14 | 0.0957 | 0.2638 | **0.0225** | 0.6641 | No |
| *HIST1H1E* | 6 | 68 | 7.3366 | 1.10E-13 | 0.1163 | **0.0030** | 0.0735 | 0.3261 | No |
| *ABT1* | 6 | 138 | 7.3345 | 1.11E-13 | 0.6842 | 0.0670 | 0.3027 | **0.0083** | No |
| *CLIC1* | 6 | 99 | 7.3131 | 1.31E-13 | 0.0716 | 0.1440 | **0.0500** | 0.5277 | No |
| *TRIM31* | 6 | 5 | 7.3004 | 1.43E-13 | 0.2428 | 0.2695 | **0.0015** | 0.7596 | No |
| *IER3* | 6 | 14 | 7.2564 | 1.99E-13 | 0.2764 | 0.2630 | **0.0032** | 0.1427 | No |
| *CYP17A1* | 10 | 133 | 7.2326 | 2.37E-13 | 0.0911 | **0.00030** | 0.8081 | 0.7482 | Yes |
| *TRIM39* | 6 | 28 | 7.2226 | 2.55E-13 | **0.0069** | **0.00037** | **0.0160** | 0.3090 | No |
| *TRIM39-RPP21* | 6 | 29 | 7.2174 | 2.65E-13 | **0.0060** | **0.00080** | **0.0096** | 0.2913 | No |
| *FLOT1* | 6 | 15 | 7.1928 | 3.17E-13 | 0.3164 | 0.4145 | **0.0039** | 0.1551 | No |
| *RPP21* | 6 | 25 | 7.1409 | 4.64E-13 | **0.0075** | **0.0165** | **0.0021** | 0.2882 | No |
| *C6orf47* | 6 | 131 | 7.1286 | 5.07E-13 | 0.4030 | 0.6668 | **0.0256** | 0.7401 | No |
| *HLA-DRA* | 6 | 599 | 7.1117 | 5.73E-13 | 0.9164 | 0.6483 | **0.0006** | 0.6919 | No |
| *ABCF1* | 6 | 5 | 7.0992 | 6.27E-13 | 0.4410 | **0.0339** | 0.2359 | 0.5237 | No |
| *MDC1* | 6 | 7 | 7.0635 | 8.12E-13 | 0.6026 | 0.5519 | **0.0427** | 0.2161 | No |
| *BTNL2* | 6 | 337 | 7.06 | 8.32E-13 | 0.6057 | 0.4857 | **0.0118** | 0.8336 | No |
| *TUBB* | 6 | 8 | 7.0307 | 1.03E-12 | 0.4086 | 0.5202 | **0.0122** | 0.2450 | No |
| *VSIG2* | 11 | 67 | 7.0299 | 1.03E-12 | 0.8393 | **0.0081** | 0.6958 | 0.6697 | Yes |
| *GPANK1* | 6 | 124 | 7.0188 | 1.12E-12 | 0.3521 | 0.6575 | **0.0238** | 0.7172 | No |
| *NRGN* | 11 | 77 | 6.9627 | 1.67E-12 | 0.7552 | **0.0117** | 0.7264 | 0.6624 | Yes |
| *BAG5* | 14 | 107 | 6.9619 | 1.68E-12 | 0.4986 | 0.1561 | **0.0137** | 0.8009 | Yes |
| *ESAM* | 11 | 68 | 6.9521 | 1.80E-12 | 0.6029 | **0.0088** | 0.6295 | 0.8493 | Yes |
| *OR11A1* | 6 | 50 | 6.9483 | 1.85E-12 | 0.5356 | 0.6890 | 0.0899 | **0.0441** | No |
| *LOC554223* | 6 | 585 | 6.9399 | 1.96E-12 | 0.2814 | 0.5510 | **0.0113** | 0.7978 | No |
| *XRCC3* | 14 | 133 | 6.9183 | 2.29E-12 | 0.8195 | 0.2144 | **0.0469** | 0.6584 | Yes |
| *AMBRA1* | 11 | 206 | 6.8573 | 3.51E-12 | 0.6131 | 0.6268 | **0.0296** | 0.3541 | Yes |
| *CYP21A2* | 6 | 81 | 6.8339 | 4.13E-12 | 0.5958 | 0.8014 | **0.0128** | 0.1559 | No |
| *HIST1H2BK* | 6 | 84 | 6.8262 | 4.36E-12 | 0.3903 | 0.1282 | 0.2083 | **0.0404** | No |
| *BCL11B* | 14 | 343 | 6.8202 | 4.55E-12 | 0.7750 | 0.0590 | 0.1645 | **0.0207** | Yes |
| *HIST1H4I* | 6 | 72 | 6.8101 | 4.88E-12 | 0.3213 | **0.0349** | 0.1392 | 0.0897 | No |
| *HIST1H2AC* | 6 | 160 | 6.7994 | 5.25E-12 | 0.5805 | 0.0623 | 0.6835 | **0.0413** | No |
| *ZSWIM6* | 5 | 425 | 6.7763 | 6.17E-12 | **0.0057** | 0.1907 | 0.3428 | 0.8775 | Yes |
| *FXR1* | 3 | 207 | 6.7535 | 7.22E-12 | **0.0175** | 0.7744 | 0.4752 | 0.8937 | Yes |
| *CSNK2B* | 6 | 120 | 6.7155 | 9.37E-12 | 0.2488 | 0.7100 | **0.0499** | 0.6756 | No |
| *POU5F1* | 6 | 521 | 6.7085 | 9.83E-12 | 0.0519 | 0.2919 | **0.0090** | 0.5160 | No |
| *TNXB* | 6 | 233 | 6.6697 | 1.28E-11 | 0.5786 | 0.5092 | **0.0458** | 0.2686 | No |
| *GNL1* | 6 | 6 | 6.6683 | 1.29E-11 | 0.3889 | **0.0317** | 0.1861 | 0.5743 | No |
| *PRR3* | 6 | 5 | 6.663 | 1.34E-11 | 0.4129 | **0.0414** | 0.2084 | 0.5200 | No |
| *MAD1L1* | 7 | 2093 | 6.6302 | 1.68E-11 | 0.6030 | 0.0997 | **0.0389** | 0.6224 | Yes |
| *HIST1H1T* | 6 | 138 | 6.6114 | 1.90E-11 | 0.6825 | 0.1534 | 0.7795 | **0.0479** | No |
| *SMG6* | 17 | 741 | 6.5757 | 2.42E-11 | 0.1161 | 0.8139 | 0.8628 | **0.0319** | Yes |
| *DGKZ* | 11 | 97 | 6.565 | 2.60E-11 | 0.3933 | 0.6024 | **0.0147** | 0.3174 | Yes |
| *APOPT1* | 14 | 165 | 6.5635 | 2.63E-11 | 0.4372 | 0.2079 | **0.0462** | 0.7811 | Yes |
| *CHRNB4* | 15 | 155 | 6.5486 | 2.90E-11 | **8.92E-24** | 0.6176 | **1.15E-05** | 0.7256 | Yes |
| *HIST1H2BC* | 6 | 164 | 6.5458 | 2.96E-11 | 0.6573 | 0.1451 | 0.7127 | **0.0413** | No |
| *TCF19* | 6 | 501 | 6.5126 | 3.69E-11 | 0.0707 | 0.3073 | **0.0060** | 0.4349 | No |
| *OR2J2* | 6 | 108 | 6.472 | 4.84E-11 | 0.1316 | **0.0401** | 0.1144 | 0.5043 | No |
| *ATG13* | 11 | 85 | 6.4353 | 6.16E-11 | 0.5597 | 0.5675 | **0.0246** | 0.4042 | Yes |
| *HIST1H2AG* | 6 | 64 | 6.4148 | 7.05E-11 | 0.3309 | **0.0084** | 0.1032 | 0.1607 | No |
| *ZNF322* | 6 | 175 | 6.387 | 8.46E-11 | 0.6407 | **0.0290** | 0.5072 | **0.0109** | No |
| *HIST1H2BJ* | 6 | 75 | 6.3852 | 8.56E-11 | 0.4850 | **0.0208** | 0.1692 | **0.0497** | No |
| *CSMD1* | 8 | 17206 | 6.3146 | 1.35E-10 | 0.5993 | **0.0327** | 0.0889 | 0.8484 | Yes |
| *SNAP91* | 6 | 403 | 6.281 | 1.68E-10 | 0.3094 | **0.0467** | 0.0795 | 0.5529 | Yes |
| *KCTD13* | 16 | 76 | 6.2504 | 2.05E-10 | 0.1560 | 0.9346 | **0.0141** | 0.3455 | Yes |
| *TRIM38* | 6 | 163 | 6.2487 | 2.07E-10 | 0.2104 | 0.4185 | 0.4980 | **0.0170** | No |
| *CCHCR1* | 6 | 599 | 6.2365 | 2.24E-10 | **0.0356** | 0.2863 | **0.0076** | 0.2027 | No |
| *TSNARE1* | 8 | 945 | 6.2362 | 2.24E-10 | **0.0296** | 0.6077 | 0.1802 | 0.1232 | Yes |
| *ZNF365* | 10 | 1061 | 6.2029 | 2.77E-10 | 0.0695 | **0.0461** | 0.3054 | **0.0366** | No |
| *SFMBT1* | 3 | 422 | 6.1938 | 2.94E-10 | **0.0499** | 0.1841 | 0.5705 | 0.8945 | No |
| *C4B* | 6 | 89 | 6.1867 | 3.07E-10 | 0.6489 | 0.6315 | **0.0247** | 0.1676 | No |
| *ASPHD1* | 16 | 80 | 6.1693 | 3.43E-10 | 0.1560 | 0.9346 | **0.0141** | 0.3455 | Yes |
| *OR10C1* | 6 | 25 | 6.1616 | 3.60E-10 | 0.5473 | 0.7535 | 0.2267 | **0.0422** | No |
| *ZFP57* | 6 | 391 | 6.1547 | 3.76E-10 | 0.3676 | 0.1118 | **0.0433** | 0.8625 | No |
| *SRPK2* | 7 | 601 | 6.1373 | 4.20E-10 | 0.6393 | 0.9103 | **0.0080** | 0.7260 | Yes |
| *C2orf82* | 2 | 136 | 6.1309 | 4.37E-10 | 0.1335 | 0.1256 | 0.1919 | **0.0268** | Yes |
| *PCCB* | 3 | 211 | 6.1149 | 4.83E-10 | 0.8166 | 0.3705 | **0.0153** | 0.7330 | Yes |
| *TRMT61A* | 14 | 121 | 6.1147 | 4.84E-10 | 0.6657 | 0.2594 | **0.0237** | 0.6266 | Yes |
| *AKT3* | 1 | 565 | 6.1111 | 4.95E-10 | 0.3935 | **0.0044** | 0.5428 | 0.8105 | Yes |
| *MOG* | 6 | 392 | 6.0526 | 7.13E-10 | 0.3674 | 0.0931 | **0.0367** | 0.8152 | No |
| *SLC45A1* | 1 | 189 | 5.9956 | 1.01E-09 | 0.4407 | 0.9673 | **0.0034** | 0.0983 | Yes |
| *STAG1* | 3 | 750 | 5.9817 | 1.10E-09 | 0.7516 | 0.4490 | **0.0322** | 0.6703 | Yes |
| *MICB* | 6 | 645 | 5.9668 | 1.21E-09 | 0.1562 | 0.7222 | **0.0015** | **0.0039** | No |
| *TMEM110-MUSTN1* | 3 | 206 | 5.9643 | 1.23E-09 | **0.0334** | 0.2598 | 0.2759 | 0.6702 | Yes |
| *COQ10B* | 2 | 61 | 5.9581 | 1.28E-09 | 0.2255 | 0.4603 | **0.0443** | 0.6883 | Yes |
| *CHRNA3* | 15 | 177 | 5.9356 | 1.46E-09 | **0.0000** | 0.8691 | **0.0001** | 0.7434 | Yes |
| *TMEM110* | 3 | 197 | 5.9075 | 1.74E-09 | **0.0341** | 0.2529 | 0.2804 | 0.6473 | Yes |
| *RFTN2* | 2 | 208 | 5.816 | 3.01E-09 | 0.1752 | 0.0939 | **0.0093** | 0.6485 | Yes |
| *PSORS1C2* | 6 | 477 | 5.7621 | 4.15E-09 | 0.0518 | 0.3086 | **0.0104** | 0.0756 | No |
| *OR2H2* | 6 | 53 | 5.7189 | 5.36E-09 | 0.4098 | **0.0378** | 0.3253 | 0.5021 | No |
| *PLA2G15* | 16 | 89 | 5.6913 | 6.30E-09 | 0.3249 | 0.5381 | 0.5429 | **0.0405** | Yes |
| *PSORS1C1* | 6 | 821 | 5.6826 | 6.63E-09 | 0.1666 | 0.4730 | **0.0177** | 0.0645 | No |
| *HIST1H2BD* | 6 | 120 | 5.6692 | 7.17E-09 | 0.3011 | **0.0047** | 0.1052 | **0.0473** | No |
| *42981* | 22 | 160 | 5.6591 | 7.61E-09 | **0.0253** | 0.9343 | 0.8467 | 0.9615 | Yes |
| *WBP2NL* | 22 | 167 | 5.6501 | 8.02E-09 | **0.0360** | 0.9105 | 0.7974 | 0.9586 | Yes |
| *DST* | 6 | 1084 | 5.6492 | 8.06E-09 | 0.3608 | **0.0421** | 0.7913 | 0.2360 | No |
| *HLA-F* | 6 | 474 | 5.6372 | 8.64E-09 | 0.7959 | 0.6650 | **0.0141** | 0.5460 | No |
| *ATXN7* | 3 | 371 | 5.6369 | 8.66E-09 | **0.0160** | 0.3256 | 0.1506 | 0.6061 | Yes |
| *KCNB1* | 20 | 489 | 5.5945 | 1.11E-08 | 0.5277 | 0.8814 | **0.0175** | 0.4645 | Yes |
| *ZNF408* | 11 | 68 | 5.5759 | 1.23E-08 | 0.5735 | 0.8185 | **0.0442** | 0.5887 | Yes |
| *ITPR3* | 6 | 553 | 5.5546 | 1.39E-08 | 0.8690 | 0.8159 | **0.0072** | 0.1751 | No |
| *CKB* | 14 | 108 | 5.5 | 1.90E-08 | 0.6623 | 0.3303 | **0.0306** | 0.3494 | Yes |
| *IREB2* | 15 | 225 | 5.4811 | 2.11E-08 | **7.13E-19** | 0.6139 | **3.14E-06** | 0.5975 | Yes |
| *CHRNA5* | 15 | 158 | 5.4527 | 2.48E-08 | **5.63E-14** | 0.8671 | **0.0014** | 0.7668 | Yes |
| *TAP1* | 6 | 379 | 5.4241 | 2.91E-08 | **0.0189** | 0.3327 | 0.1103 | 0.6506 | No |
| *ESRP2* | 16 | 85 | 5.4174 | 3.02E-08 | 0.4818 | 0.4746 | 0.8198 | **0.0212** | Yes |
| *KCNJ13* | 2 | 117 | 5.4059 | 3.23E-08 | 0.0764 | 0.1345 | 0.1809 | **0.0383** | Yes |
| *MSANTD2* | 11 | 171 | 5.3944 | 3.44E-08 | 0.2186 | **0.0263** | 0.4964 | 0.7285 | Yes |
| *WBP1L* | 10 | 314 | 5.331 | 4.88E-08 | **0.0412** | **0.0060** | 0.8064 | 0.5326 | Yes |
| *FOXO3* | 6 | 280 | 5.3269 | 5.00E-08 | 0.4193 | **0.0416** | 0.7926 | 0.7174 | No |
| *ZNF804A* | 2 | 823 | 5.3043 | 5.66E-08 | **0.0040** | 0.0910 | 0.1726 | 0.6520 | Yes |
| *HMGN4* | 6 | 125 | 5.3031 | 5.69E-08 | 0.4326 | 0.1615 | 0.8288 | **0.0035** | No |
| *IP6K3* | 6 | 295 | 5.2811 | 6.42E-08 | 0.3386 | 0.7008 | **0.0013** | 0.1199 | No |
| *OR12D2* | 6 | 128 | 5.2163 | 9.13E-08 | 0.4982 | 0.3770 | **0.0121** | 0.4004 | No |
| *THOC7* | 3 | 124 | 5.2067 | 9.61E-08 | **0.0337** | 0.2091 | 0.0604 | 0.3923 | Yes |
| *ADAMTS2* | 5 | 962 | 5.1767 | 1.13E-07 | **0.0377** | 0.2160 | 0.5924 | 0.7013 | No |
| *PSMB9* | 6 | 350 | 5.1299 | 1.45E-07 | **0.0089** | 0.3268 | 0.1627 | 0.5600 | No |
| *TRAF3IP2* | 6 | 254 | 5.1235 | 1.50E-07 | 0.8909 | **0.0235** | 0.2517 | 0.5862 | No |
| *THRB* | 3 | 1258 | 5.0906 | 1.78E-07 | 0.7384 | **0.0034** | 0.1596 | 0.5359 | No |
| *PLCL1* | 2 | 701 | 5.0868 | 1.82E-07 | 0.2028 | 0.1070 | **0.0098** | 0.9012 | Yes |
| *RERE* | 1 | 806 | 5.0654 | 2.04E-07 | 0.1191 | 0.9095 | **0.0055** | 0.2331 | Yes |
| *TBC1D5* | 3 | 1454 | 5.0543 | 2.16E-07 | **0.0417** | 0.8829 | 0.2011 | 0.4676 | Yes |
| *ZDHHC2* | 8 | 322 | 5.0359 | 2.38E-07 | 0.6840 | 0.4507 | 0.9918 | **0.0052** | No |
| *SORCS3* | 10 | 1982 | 5.0029 | 2.82E-07 | 0.3604 | **0.0151** | **0.0169** | 0.6810 | No |
| *KDM4A* | 1 | 178 | 4.999 | 2.88E-07 | 0.9581 | **0.0051** | 0.0902 | 0.3298 | Yes |
| *ZBED4* | 22 | 269 | 4.9895 | 3.03E-07 | 0.0549 | 0.2682 | 0.2906 | **0.0182** | No |
| *PTPRF* | 1 | 342 | 4.9684 | 3.37E-07 | 0.8816 | **0.0087** | 0.0865 | 0.4770 | Yes |
| *MAN2A1* | 5 | 764 | 4.9597 | 3.53E-07 | 0.3757 | **0.0028** | 0.6622 | 0.4060 | Yes |
| *RPRD2* | 1 | 470 | 4.939 | 3.93E-07 | **0.0381** | 0.6542 | 0.9236 | 0.4704 | No |
| *APH1A* | 1 | 53 | 4.929 | 4.13E-07 | **0.0365** | 0.5280 | 0.7770 | 0.4764 | Yes |
| *BOLL* | 2 | 136 | 4.8739 | 5.47E-07 | 0.1258 | 0.0948 | **0.0074** | 0.7130 | Yes |
| *TCF20* | 22 | 364 | 4.8692 | 5.60E-07 | **0.0406** | 0.4198 | 0.6259 | 0.8814 | Yes |
| *CPNE7* | 16 | 238 | 4.864 | 5.75E-07 | 0.6747 | **0.0033** | **0.0277** | 0.4663 | No |
| *SLC16A10* | 6 | 327 | 4.8604 | 5.86E-07 | **0.0262** | 0.1521 | 0.2742 | 0.8248 | No |
| *FAM134A* | 2 | 100 | 4.8461 | 6.30E-07 | 0.6418 | 0.5023 | **0.0055** | 0.5437 | No |
| *DNAJC11* | 1 | 248 | 4.8087 | 7.60E-07 | 0.4195 | 0.1790 | 0.3914 | **0.0228** | No |
| *EP300* | 22 | 205 | 4.7979 | 8.02E-07 | **0.0392** | 0.2737 | 0.5964 | 0.3288 | Yes |
| *ANK3* | 10 | 2335 | 4.7979 | 8.02E-07 | 0.8187 | **0.0224** | 0.5988 | 0.0682 | No |
| *LCAT* | 16 | 40 | 4.7781 | 8.85E-07 | 0.7319 | 0.7817 | 0.4010 | **0.0056** | Yes |
| *CLU* | 8 | 162 | 4.7693 | 9.24E-07 | **0.0161** | 0.0601 | 0.5940 | 0.1558 | Yes |
| *GABBR2* | 9 | 1823 | 4.7629 | 9.54E-07 | 0.8359 | 0.6512 | 0.3259 | **0.0229** | No |
| *SLC12A4* | 16 | 69 | 4.7423 | 1.06E-06 | 0.7343 | 0.7670 | 0.3583 | **0.0053** | Yes |
| *NFATC3* | 16 | 295 | 4.7275 | 1.14E-06 | 0.3786 | 0.4573 | 0.8004 | **0.0386** | Yes |
| *CDSN* | 6 | 620 | 4.7223 | 1.17E-06 | 0.2405 | 0.5903 | 0.0572 | **0.0347** | No |
| *PSMB10* | 16 | 36 | 4.7219 | 1.17E-06 | 0.7936 | 0.8307 | 0.3371 | **0.0044** | Yes |
| *HYI* | 1 | 63 | 4.7208 | 1.17E-06 | 0.5529 | **0.0121** | 0.4927 | 0.2067 | No |
| *TARS2* | 1 | 179 | 4.718 | 1.19E-06 | **0.0339** | 0.6279 | 0.9022 | 0.6413 | No |
| *EYS* | 6 | 6248 | 4.6757 | 1.46E-06 | 0.5689 | 0.0631 | 0.1566 | **0.0044** | No |
| *DPEP3* | 16 | 59 | 4.669 | 1.51E-06 | 0.5429 | 0.6939 | 0.4344 | **0.0096** | Yes |
| *MLN* | 6 | 197 | 4.6672 | 1.53E-06 | 0.0721 | 0.3996 | **0.0016** | 0.2353 | No |
| *SEMA6D* | 15 | 1938 | 4.6565 | 1.61E-06 | 0.1764 | **0.0040** | **6.38E-07** | **0.0019** | No |
| *PHF2* | 9 | 604 | 4.6399 | 1.74E-06 | **0.0443** | 0.3068 | 0.4129 | **0.0383** | No |
| *TEK* | 9 | 902 | 4.635 | 1.79E-06 | 0.5393 | **0.0436** | 0.1345 | 0.3271 | No |
| *CXXC5* | 5 | 118 | 4.6321 | 1.81E-06 | 0.5349 | 0.2986 | 0.7309 | **0.0388** | No |
| *POMC* | 2 | 111 | 4.6246 | 1.88E-06 | 0.3487 | 0.2128 | 0.9742 | **0.0168** | No |
| *SZT2* | 1 | 163 | 4.6229 | 1.89E-06 | 0.5856 | **0.0148** | 0.4485 | 0.2125 | No |
| *CNPPD1* | 2 | 107 | 4.6098 | 2.02E-06 | 0.5870 | 0.5346 | **0.0072** | 0.3962 | No |
| *UQCC2* | 6 | 228 | 4.5984 | 2.13E-06 | 0.8406 | 0.8376 | **0.0057** | 0.0693 | No |
| *EIF5* | 14 | 171 | 4.5805 | 2.32E-06 | 0.8206 | **0.0057** | 0.6903 | 0.1367 | No |
| *MFHAS1* | 8 | 759 | 4.5782 | 2.35E-06 | 0.9261 | 0.2358 | **0.0325** | **0.0205** | No |
| *HIST1H4H* | 6 | 150 | 4.5762 | 2.37E-06 | 0.7816 | **0.0408** | **0.0351** | **0.0205** | No |
| *HLA-DPA1* | 6 | 924 | 4.5735 | 2.40E-06 | **5.95E-05** | 0.7403 | 0.2087 | 0.3141 | No |
| *DPEP2* | 16 | 73 | 4.5718 | 2.42E-06 | 0.4273 | 0.6775 | 0.4835 | **0.0259** | Yes |
| *HIST1H2BI* | 6 | 126 | 4.5713 | 2.42E-06 | 0.7970 | **0.0268** | **0.0309** | **0.0133** | No |
| *BRD1* | 22 | 275 | 4.5585 | 2.58E-06 | 0.1727 | 0.1850 | 0.3680 | **0.0146** | No |
