# Supplementary material for: Identification of 34 genes conferring genetic and pharmacological risk for the comorbidity of schizophrenia and smoking behaviors
Source: Aging (Albany NY). 2020 Feb 3;12(3):2169–225. doi: 10.18632/aging.102735 (PMC7041787; doi:10.18632/aging.102735)
Supplement: Supplementary Tables 4, 6, 7 [file aging-12-102735-s006..pdf]

SUPPLEMENTARY TABLES

Supplementary Table 4. Module-level statistics.

Statistics	Modules					
	Blue	Brown	Green	Red	Turquoise	Yellow
Orbital prefrontal (OFC)	4.93	8.42	8.96	7.57	7.89	8.27
Dorsolateral prefrontal (DFC)	4.95	8.39	8.95	7.64	7.90	8.23
Ventrolateral prefrontal (VFC)	4.93	8.40	8.97	7.62	7.90	8.10
Medial prefrontal (MFC)	4.94	8.36	8.92	7.61	7.88	8.25
Primary motor (M1C)	4.95	8.37	8.93	7.58	7.87	7.50
Primary somatosensory (S1C)	4.97	8.36	8.93	7.57	7.87	7.49
Inferior parietal (IPC)	4.97	8.37	8.93	7.56	7.86	7.49
Primary auditory (A1C)	4.96	8.37	8.94	7.55	7.86	7.49
Superior temporal (STC)	4.97	8.36	8.93	7.56	7.86	8.07
Inferior temporal (ITC)	4.97	8.37	8.91	7.57	7.85	8.07
Primary visual (V1C)	4.96	8.27	8.89	7.49	7.81	7.48
Hippocampus (HIP)	4.95	8.20	8.86	7.40	7.80	8.15
Amygdala (AMY)	4.96	8.23	8.88	7.45	7.80	8.17
Striatum (STR)	4.94	8.03	8.88	7.27	7.70	7.35
Thalamus (MD)	4.95	8.09	8.88	7.21	7.67	7.34
Cerebellum (CBC)	4.93	7.90	8.85	7.04	7.52	7.14
max(log2(expression))	4.97	8.42	8.97	7.64	7.90	8.27
min(log2(expression))	4.93	7.90	8.85	7.04	7.52	7.14
max(log2(FC))	0.05	0.52	0.12	0.60	0.38	1.12
8-10 weeks (stage2)	4.92	7.59	8.99	7.32	7.66	8.27
10-13 weeks (stage3)	5.01	8.12	9.16	8.18	7.78	8.08
13-16 weeks (stage4)	5.01	8.03	9.13	8.21	7.73	8.15
16-19 weeks (stage5)	4.97	8.22	9.03	8.27	7.77	8.06
19-24 weeks (stage6)	4.88	7.77	8.91	7.52	7.34	7.34
24-38 weeks (stage7)	5.13	8.41	9.17	8.56	7.98	8.05
0-0.5 years (stage8)	5.16	8.58	9.13	8.80	8.07	8.08
0.5-1 years (stage9)	5.25	8.74	8.97	8.72	7.85	7.78
1-6 years (stage10)	5.26	8.50	8.69	8.47	7.84	7.73
6-12 years (stage11)	5.04	8.72	8.98	8.52	7.87	7.70
12-20 years (stage12)	5.09	8.71	8.84	8.51	7.80	7.71
20-40 years (stage13)	5.08	8.73	8.94	8.43	7.83	7.67
40-60 years (stage14)	5.21	8.70	8.91	8.33	7.81	7.64
60+ years (stage15)	5.19	8.60	8.78	8.24	7.79	7.63
max($\log_2$(expression))	5.26	8.74	9.17	8.80	8.07	8.27
min($\log_2$(expression))	4.88	7.59	8.69	7.32	7.34	7.34
max($\log_2$(FC))	0.38	1.15	0.48	1.47	0.73	0.92

Note: For both regional expression patterns and temporal expression patterns, we used the method of mean $\log_2$(expression) of all genes in each module.

Supplementary Table 6. 16 of 34 smoking- and SCZ-associated methylation genes have been reported to be associated with SCZ in previous studies.

Gene Name	Description	References
AKT3	Genetic variation in the *AKT3* locus (chr1:243503719–244002945) is a top GWAS signal in schizophrenia and pathway analysis identified 50 single nucleotide polymorphisms (SNPs) within the *AKT3* gene that contribute to four of the top pathways associated with risk for schizophrenia and bipolar disorder. *AKT3* shows prenatal enrichment during human neocortical development and recurrent copy number variations involving the 1q43-44 locus are associated with cortical malformations and intellectual disability, implicating an essential role in early brain development.	[1, 14–17]
AP2A2	Schizophrenia-based genetic association study shows the involvement of the clathrin-mediated endocytosis (CME)-related protein enthoprotein encoded by the clathrin interactor 1 (*CLINT1*) gene. The expression of genes encoding adaptor and clathrin assembly proteins, *AP2A2, AP2B1, AP180, CLINT1, HIP1, ITSN2*, and *PICALM*, increased relative to the control in SH-SY5Y cells incubated with 5–10 µmol/l clozapine for 24–72 h.	[18]
CACNA1D	The animal model of neonatal lesion in ventral hippocampus (NLVH) is a recognized animal model for schizophrenia. NLVH influenced change expressions in various genes involved in Ca^{2+} homeostasis, including Cacna1d, Atp2a2, Adcy2, Ppp3cb, and Ptk2b.	[19–21]
CDKN1A	Quetiapine is an atypical neuroleptic with a pharmacological profile distinct from classic neuroleptics that function primarily via blockade of dopamine D2 receptors. In the United States, quetiapine is currently approved for treating patients with schizophrenia, major depression and bipolar I disorder. Despite its widespread use, its cellular effects remain elusive. To address possible mechanisms, we chronically treated mice with quetiapine, haloperidol or vehicle and examined quetiapine-specific gene expression change in the frontal cortex. Through microarray analysis, we observed that several groups of genes were differentially expressed upon exposure to quetiapine compared with haloperidol or vehicle; among them, Cdkn1a, the gene encoding p21, exhibited the greatest fold change relative to haloperidol. The quetiapine-induced downregulation of p21/Cdkn1a was confirmed by real-time polymerase chain reaction and in situ hybridization. Consistent with single gene-level analyses, functional group analyses also indicated that gene sets associated with cell cycle/fate were differentially regulated in the quetiapine-treated group. In cortical cell cultures treated with quetiapine, p21/Cdkn1a was significantly downregulated in oligodendrocyte precursor cells and neurons, but not in astrocytes. We propose that cell cycle-associated intervention by quetiapine in the frontal cortex may underlie a unique efficacy of quetiapine compared with typical neuroleptics.	[12]
CNTNAP2	Contactin associated protein-like 2 (CNTNAP2) has emerged as a prominent susceptibility gene implicated in multiple complex neurodevelopmental disorders, including autism spectrum disorders (ASD), intellectual disability (ID), and schizophrenia (SCZ). Based on genomic rearrangements and copy number variations, the contactin-associated protein-like 2 gene (CNTNAP2) has been implicated in neurodevelopmental disorders such as Gilles de la Tourette syndrome, intellectual disability, obsessive compulsive disorder, cortical dysplasia-focal epilepsy syndrome, autism, schizophrenia, Pitt-Hopkins syndrome, and attention deficit hyperactivity disorder.	[22–24]
GNA12	*GNA12* is significantly associated with schizophrenia based on network-assisted investigation of combined causal signals from GWAS studies in schizophrenia.	[25]
GPSM3	*GPSM3* is significantly associated with schizophrenia based on a multi-stage schizophrenia GWAS.	[1]
HRH1	*HRH1* is significantly associated with schizophrenia based on genetic association study.	[26, 27]
KCNQ1	Patients with schizophrenia show decreased processing speed on neuropsychological testing and decreased white matter integrity as measured by diffusion tensor imaging, two traits shown to be both heritable and genetically associated indicating that there may be genes that influence both traits as well as schizophrenia disease risk. The potassium channel gene family is a reasonable candidate to harbor such a gene given the prominent role potassium channels play in the central nervous system in signal transduction, particularly in myelinated axons. *KCNQ1* may contribute to the shared risk for diminished processing speed, diminished white mater integrity and increased risk of schizophrenia.	[28, 29]
MAD1L1	Multiple lines of evidence from genetic association studies indicate *MAD1L1* confer risk to schizophrenia.	[1, 30, 31]

NOS1AP	*NOS1AP* is a protein implicated in schizophrenia. Several independent studies reported linkage of schizophrenia to chromosome 1q21–22, containing *NOS1AP*. Many molecular functional-based and genetic-based studies have been identified *NOS1AP* gene as a schizophrenia susceptibility gene.	[32–38]
NOTCH1	The antipsychotic and myelin protective effects of quetiapine are mediated by Notch signaling in a mouse model of cuprizone-induced demyelination associated with schizophrenia-like behaviors. The Notch pathway might therefore be a novel target for the development of antipsychotic drugs.	[39]
PARD3	Based on a genetic-based association study, *PARD3* is associated with susceptibility to schizophrenia in a Korean population.	[40]
RGS12	*RGS12* is putative candidate genes for sporadic schizophrenia.	[41]
TNF	TNF-α is associated with the deficit syndrome and negative symptoms in patients with chronic schizophrenia.	[42, 43]
TRIO	Disrupted-in-Schizophrenia 1-mediated axon guidance involves TRIO-RAC-PAK small GTPase pathway signaling	[44]

Supplementary Table 7. Differential expressed genes in SCZ patients and controls divided by smoking status.

Gene Name	SCZ VS CTRL	Smoking VS Nonsmoking	Anova P value[a]
PTPRN2	0.0000518	0.316	0.00142
ARHGAP25	0.0001	0.0028	0.00026
ARHGEF3	0.000281	0.11	0.00723
AKT3	0.00111	0.381	0.0395
RGS12	0.00202	0.347	0.0058
SORBS1	0.0134	0.471	0.108
TIAM2	0.0161	0.954	0.062
CACNA1D	0.0162	0.403	0.052
HTT	0.0179	0.259	0.132
NOTCH1	0.0239	0.301	0.156
PLEK	0.0256	0.0383	0.043
RPS6KA2	0.0268	0.815	0.112
PARD3	0.0296	0.033	0.054
TRIO	0.0326	0.324	0.147
CNTNAP2	0.0344	0.062	0.087
GPSM3	0.0369	0.353	0.0014
ZMIZ1	0.0708	0.499	0.041
TBC1D14	0.845	0.0389	0.030

Note: *anova* statistical test was used for comparing the significant differences between SCZ patients and controls grouped by smoking status.
