# Supplementary material for: Identification of 34 genes conferring genetic and pharmacological risk for the comorbidity of schizophrenia and smoking behaviors
Source: Aging (Albany NY). 2020 Feb 3;12(3):2169–225. doi: 10.18632/aging.102735 (PMC7041787; doi:10.18632/aging.102735)
Supplement: Supplementary Table 5 [file aging-12-102735-s002..docx]

**Supplemental Table 5. 149 coexpression module genes involved in smoking- and SCZ-associated methylation**

| **Gene names** | **Smoking-associated methylation genes with one piece of evidence** | **Smoking-associated methylation genes with two pieces of evidence** | **SCZ-associated methylation genes** |
| --- | --- | --- | --- |
| *PRKCZ* | Yes | Yes | Yes |
| *ESPN* | Yes | No | Yes |
| *PER3* | Yes | No | Yes |
| *CLCN6* | Yes | Yes | Yes |
| *ARHGEF10L* | Yes | No | Yes |
| *RPS6KA1* | Yes | No | Yes |
| *HDAC1* | Yes | No | No |
| *CELSR2* | Yes | No | Yes |
| *PIP5K1A* | Yes | No | Yes |
| *SEMA4A* | Yes | No | Yes |
| *NOS1AP* | Yes | Yes | Yes |
| *ATP1B1* | Yes | No | Yes |
| *FASLG* | Yes | No | Yes |
| *ATP2B4* | Yes | No | Yes |
| *CLSTN1* | Yes | No | Yes |
| *DFFA* | Yes | No | No |
| *UBR4* | Yes | No | Yes |
| *EDN2* | Yes | Yes | No |
| *SLC2A1* | Yes | No | Yes |
| *TGFBR3* | Yes | Yes | Yes |
| *ARHGAP29* | Yes | No | Yes |
| *CNN3* | Yes | No | Yes |
| *SORT1* | Yes | No | Yes |
| *KCNA3* | Yes | No | Yes |
| *THEM4* | Yes | No | No |
| *LMX1A* | Yes | No | Yes |
| *SYT2* | Yes | No | Yes |
| *RABIF* | Yes | No | No |
| *DUSP10* | Yes | No | Yes |
| *ENAH* | Yes | No | Yes |
| *AKT3* | Yes | Yes | Yes |
| *PPP1CB* | Yes | No | Yes |
| *SOCS5* | Yes | No | No |
| *PLEK* | Yes | Yes | Yes |
| *ARHGAP25* | Yes | Yes | Yes |
| *MAP4K4* | Yes | No | Yes |
| *CXCR2* | Yes | No | Yes |
| *ALPPL2* | Yes | Yes | Yes |
| *ALPI* | Yes | Yes | Yes |
| *INPP5D* | Yes | No | Yes |
| *XPO1* | Yes | No | No |
| *TGFA* | Yes | No | Yes |
| *BIN1* | Yes | No | Yes |
| *ITGB6* | Yes | No | Yes |
| *NDUFS1* | Yes | No | No |
| *KLF7* | Yes | No | Yes |
| *BARD1* | Yes | No | No |
| *HTR2B* | Yes | No | No |
| *NMUR1* | Yes | No | Yes |
| *HRH1* | Yes | Yes | Yes |
| *FGD5* | Yes | No | Yes |
| *TGFBR2* | Yes | No | Yes |
| *CACNA1D* | Yes | Yes | Yes |
| *CACNA2D3* | Yes | No | Yes |
| *TIGIT* | Yes | Yes | Yes |
| *PLXNA1* | Yes | No | Yes |
| *NCK1* | Yes | No | Yes |
| *MRAS* | Yes | No | Yes |
| *RTP1* | Yes | No | Yes |
| *PAK2* | Yes | No | Yes |
| *MYL3* | Yes | No | No |
| *CISH* | Yes | No | No |
| *ARHGEF3* | Yes | Yes | Yes |
| *UBA3* | Yes | No | Yes |
| *FOXP1* | Yes | No | Yes |
| *CPOX* | Yes | Yes | Yes |
| *PLD1* | Yes | No | Yes |
| *HTT* | Yes | Yes | Yes |
| *RGS12* | Yes | Yes | Yes |
| *TBC1D14* | Yes | Yes | Yes |
| *CD38* | Yes | Yes | No |
| *SLIT2* | Yes | No | Yes |
| *GSX2* | Yes | No | No |
| *ATOH1* | Yes | No | Yes |
| *CPLX1* | Yes | No | Yes |
| *CLNK* | Yes | No | Yes |
| *UNC5C* | Yes | No | Yes |
| *LEF1* | Yes | No | Yes |
| *CAMK2D* | Yes | No | Yes |
| *TRIO* | Yes | Yes | Yes |
| *FER* | Yes | No | Yes |
| *PURA* | Yes | No | Yes |
| *DOCK2* | Yes | No | Yes |
| *NPM1* | Yes | No | No |
| *RGS14* | Yes | No | Yes |
| *SLC12A7* | Yes | No | Yes |
| *CTNND2* | Yes | No | Yes |
| *CDH12* | Yes | No | No |
| *MEF2C* | Yes | No | Yes |
| *PPP2CA* | Yes | No | No |
| *PCDH12* | Yes | No | Yes |
| *CAMK2A* | Yes | No | Yes |
| *TNF* | Yes | Yes | Yes |
| *CDKN1A* | Yes | Yes | Yes |
| *ENPP1* | Yes | No | No |
| *TIAM2* | Yes | Yes | Yes |
| *SYNJ2* | Yes | No | Yes |
| *GPSM3* | Yes | Yes | Yes |
| *TAP1* | Yes | No | Yes |
| *DAXX* | Yes | No | Yes |
| *HTR1B* | Yes | No | Yes |
| *NDUFAF4* | Yes | No | No |
| *MAP3K5* | Yes | No | Yes |
| *NUP43* | Yes | No | Yes |
| *RPS6KA2* | Yes | Yes | Yes |
| *RALA* | Yes | No | Yes |
| *TRIP6* | Yes | No | Yes |
| *SH2B2* | Yes | No | Yes |
| *CALD1* | Yes | No | Yes |
| *CNTNAP2* | Yes | Yes | Yes |
| *MAD1L1* | Yes | Yes | Yes |
| *GNA12* | Yes | Yes | Yes |
| *INHBA* | Yes | No | Yes |
| *STX1A* | Yes | No | Yes |
| *SEMA3C* | Yes | No | Yes |
| *PTPRN2* | Yes | Yes | Yes |
| *ARHGEF10* | Yes | No | Yes |
| *ZFPM2* | Yes | No | Yes |
| *PSD3* | Yes | No | Yes |
| *GNRH1* | Yes | No | No |
| *DUSP4* | Yes | Yes | Yes |
| *PLAT* | Yes | No | Yes |
| *SNTB1* | Yes | No | Yes |
| *KCNQ3* | Yes | No | Yes |
| *PTK2* | Yes | Yes | No |
| *DAPK1* | Yes | No | Yes |
| *RABGAP1* | Yes | No | Yes |
| *SLC24A2* | Yes | No | Yes |
| *CD72* | Yes | No | Yes |
| *RAPGEF1* | Yes | No | Yes |
| *RALGDS* | Yes | No | Yes |
| *NOTCH1* | Yes | Yes | Yes |
| *GAD2* | Yes | No | No |
| *RAB18* | Yes | No | No |
| *ZMIZ1* | Yes | Yes | Yes |
| *ADRA2A* | Yes | Yes | No |
| *TCF7L2* | Yes | No | Yes |
| *SLC18A2* | Yes | No | Yes |
| *CUBN* | Yes | No | Yes |
| *PARD3* | Yes | Yes | Yes |
| *ARHGAP22* | Yes | No | Yes |
| *SORBS1* | Yes | Yes | Yes |
| *FGF8* | Yes | No | No |
| *RGS10* | Yes | No | Yes |
| *CTBP2* | Yes | No | Yes |
| *AP2A2* | Yes | Yes | Yes |
| *KCNQ1* | Yes | Yes | Yes |
| *DGKZ* | Yes | No | Yes |
| *PTPRJ* | Yes | No | Yes |
